# Supplementary material for: Gendered lives, gendered Vulnerabilities: An intersectional gender analysis of exposure to and treatment of schistosomiasis in Pakwach district, Uganda
Source: PLoS Negl Trop Dis. 2023 Nov 10;17(11):e0010639. doi: 10.1371/journal.pntd.0010639 (PMC10684070; doi:10.1371/journal.pntd.0010639)
Supplement: S1 Data — (ZIP) [file pntd.0010639.s001.zip › FGD Schisto Interviews/FGD 18-45 FEMALE YRS PANYIMUR.docx]

**GENDER INTERSECTIONALITY**

**AND**

**SCHISTOSOMIASIS IN RURAL UGANDA**

**TRANSCRIPTION FOR FOCUSED GROUP DISCUSSION.**

# Abbreviations and acronyms

FGD – Focus Group Discussion

GP2 –Group Two.

F1-Facilitator 1

F2-Facilitator 2

Mod-Moderator

P1-Participant 1

P2-Participant 2

P3-Participant 3

P4-Participant 4

P5-Participant 5

**GP3.FEMALE (18-45) FGD**

**F2;** thank you for coming and I hope you understand alur language?

**All participants**; yes.

**F2;** you are group three age bracket of eighteen to forty five. I think we have all reached that age. We are going to introduce ourselves one by one. Mama the baby there…

**Mod;** am suggesting that you the baby, she may touch the electric cables.

**F2;** we are going to start from here.You will introduce yourself and the village you are coming from.

**P1;** am Awekonimungu Rovia singilla central

**P2**; Akumu Lillian singilla B

**P3;** Pimer pa Mungu Fortunate.

**P4;** Olul Florence

**F2;** Florence and the other name?

**P4;** Olul Florence Singilla B

**P5;** Ayiorwoth Sunday from singilla B

**F2;** ok, am name is Noah Okumu, Panyimur south (laughs …)

**F2;** she is call Salama, Peter and Oryema.

**F2;** we are going to ask some questions but first we have to fill the consent form before us to show that we have agreed to participate in this focus group discussion. You will sign at the last point there.

**F2;**The consent consist of the purpose of the study, the procedures, what we are going to consider and lastly were you will agree that you have read or you have been read to the condition of the study and anytime you want to withdraw, you can with withdraw without telling us why you are withdrawing. What comes from this study will not affect you in one way or the other and that is the paper you are holding now.

**F2;**Secondly as we earlier talked, we discuss something about bilharzia and some facts about bilharzia, to say we shall give them before we go on. About bilharzia for example,

**F2;** In the whole of Uganda it has been found out that three(3) out of Ten (10) do have bilharzia and when we are to look at our Pakwach ,let’s take Panyimur, it has been found out that five (5) out of ten meaning that minimum of 5 five people are having bilharzia.

**F2;** If am to take just last week children near Nyakagei primary school were tested up there, people from the singilla south could be knowing about this, Nyakagei 108 children were tested all of them had bilharzia, let’s take like that in kivujje 104 were tested, only 2 were not having the worms, but the rest of 102 had bilharzia, so ours here what they say 5/10 here, it is not there. It is 10/10 are having the bilharzia disease; you can imagine the magnitude of the problem.

**F2;**And out of these ten people if they are to be given praziquantel, only seven can get healed and the 3 three will not get cured and will continue infecting us.

So this day we have been giving the drugs to the children at school and the drug is not for selling, it is the government to supply this drugs because it is expensive to buy.so the children in primary and in secondary so it’s not easy to get this drug.so we are going to see between women and men, this drug how can we use it in order to prevent this disease.

**F2;** so we have questions and these questions when one person is answering, the others have to listen and secondly you will remove the mask a bit so that our voices can be heard. What has been put in the middle here is to record our voices but others are being written down.

**F2;** we have a total of twelve (12) of them exactly and all of us I think will answer one by one. Your thoughts/suggestion or you will give us what you see, how you see them or your experiences.

Your thoughts/suggestion or you will give us what you see, how you see them or your experiences.

**F2;** so what activities do you or your family or relatives perform that might lead to infection with schistosomiasis?

**F2;** so before we continue, which language should we use Olul,

**P4;** we use English and mix at some point

**F2;** your name again,

**P3;** Pimer Pa Mungu Fortunate

**F2;** so Fortunate, should we use English strictly?

**P3;** we can use English but for other people, we can mix.

**F2;** ok, we shall use English as per now and we shall be mixing and once you are not getting anything well just ask us we shall explain.

**F2;** **so what activities do you or your family or relatives perform that might lead to infection with schistosomiasis?**

**F2**; when you want to speak you can show hands

**F2;** yes, Fortunate,

**P3;**one is that the fishermen, at times they have gone to fish and most time they will be in water which may be having this worms and they end up getting infected.

**F2;** fishing activities

**F2**; yes

**P5;**as for me personally I do go there to fetch water from the lake of which that water might be infected with bilharzia and even my siblings do go there to fetch water and

**F2;** so fetching of water

**P2;**ok,for me what I can say about that are the farmers as they farm along the lakes or swamps without putting on boots they can get this bilharzia.

**P1;** some people do enter into the water to collect snails and through this bilharzia can enter into their body.

**F2;** snail mining

**P4;** for me what I can say is farming activity along the lakes by some farmers whereby at some times, snails tend to be blown to the gardens were they are digging without any protection and the bilharzia can enter their body from there.

**F2;** what other activities?

**F2**; Fortunate;

**P3;** swimming

**F2;** swimming.do you also swim?

**P3;** no.

**F2;** our second question is …

**F2; why are men more likely to be infected than women in some communities?**

**P5;** what I see here most men do bath from the river and most of the fishing activities are done by the men.

**F2;** Olul,

**P4;**what I wanted to say has been mentioned ,but additional point could be drugs, most time when drugs are being distributed men tend to fear to take the drugs this is what I should be very opened with than women.

**P2;** as for me, I have seen most men like swimming in the water that can bring infection to them that’s why their percentage is high.

**F2;** any other reasons or anything that make you think that men are more likely to be infected than women in some communities?

**F2;** ok, on the other hand,

**F2; why are women or their children more likely to be infected in some communities?**

**P3**; pardoned

**F2;** he repeats the question, “why are women or their children more likely to be infected in some communities?” you can tell us about children and then women.

**F2;** Fortunate,

**P3;** for children, for them they like playing in dirty waters and at times they swimming and open defecation and the places may be infected.

**F2;** yes, your name again?

**P1**; Rovia,

**F2;** yes, Rovia,

**P1;** women do get it from fetching of water as they go and stand in the water for some times as they fetch and also washing of clothes.

**F2;** so fetching of water and washing of clothes

**F2;** yes Olul,

**P4;** secondly, this water fetched by women are never left under sunshine or boiled. They use it direct for bathing children, cooking and drinking that leads to infection.

**F2;** women tend to use untreated water most.

**F1;**like this other point that, women after fetching water they just use the water for bathing themselves and children while the men bath boiled water.

**F1, F2, All participants ;(** laughs…)

**F2;** ok.

**F2; what changes in lifestyle can you or your family make to prevent you from getting schistosomiasis?**

**F2;** yes fortunate,

**P3;** use of treated water

**F2;** use of treated water

**F2**; Olul

**P4;** health education

**F2;** health education.

**P4;** sensitized

**F2;** sensitized not teaching.

**P2;** building nice pit latrines.

**F2;** yes, Sunday,

**P5;** preventing children from swimming

**P1;** educating community about open defecation

**F2;** educating about open defecation to continue or

**P1;** no to stop.

**F2;** any other lifestyles.

**P4;**in case there is this program of distribution of drugs ,people should take the medicine because some people fear and children above five years should also be given to prevent the level of infections.

**Mod;** change in the mind set as many people tend to fear.

**F2;** once they take the drug, the worm level reduces and the infection reduces in the community.

**F2;** yes

**P2;** boiling of drinking water and for bathing

**F2;** so treatment of water or boiling

**F2;** lets increase our voices a bit

**F2;** **what changes in your community or health systems or local government would help control or eradicate schistosomiasis from your community?**

**F2;** yes number 5.

**P2;** door to door distribution/giving of drugs

**F2;** who should give the drugs?

**P2;** the government should give drugs to the people.

**F2;** yes Sunday,

**P5;**for me suggesting that they should build pit latrine along the landing sites like what the fisheries did when people used to defecate anyhow and a pit latrine was built and helped people.

**F2;** who should construct these public toilet, community or government?

**P5;** the community should also bring support to help people because along the landing sites normally do open defecation.

**F2;** so construction of public toilet

**F1;** in danger spots

**F2;** and as for Panyimur I think it’s everywhere.

**Mod;** and as you build the toilet, you guys here you must use it and take responsibility of cleaning it otherwise you will find stuff from outside up to inside.

**F1;** I went to Mubogo landing site saw a line… (Laughing….)

**F2;** they start it from inside and come to finish it from outside.

**F2;** any other changes from the community or health systems or local government?

**F2;** yes Olul,

**P4;** the government should sensitized the community about the dangers of bilharzia so that they can change.

**F2;** continuous sensitization

**P3;** the government should provide community with water guide.

**F2;** water treatment

**F2;** any other changes that will help prevent or eradicate bilharzia from our communities

**F2;** yes Olul,

**P4;** I think the drugs should be in plenty because I have seen the medicines are being given after five or ten years because at times…

**F2;** it should be in plenty or frequently?

**P4;** that’s it may be after six months or one year like someone who has already bilharzia and is in bad state should be given medicines more frequently.

**F2;** so to get our opinion and the drug should be given more frequently especially to those who are already having bilharzia.

**F2; has your family ever discussed use of praziquantel or any ways to prevent schistosomiasis? If they have what are their opinions?**

**F2;** that is experience now each one of us can tell us.

**P4;** in family we have ever discussed about bilharzia. We have some children who like going to the lake side whenever we are not around. Some children are stubborn and they don’t listen as sometimes you can be at the garden or market on coming back you will find the child is at lake side or already swimming.

**F2;** ok, when you discussed about it how did they respond?

**P4;** the children responded positively.

**P2;**in our family, our mother discussed about bilharzia when we used to go to the lake at most time and by that time the disease was too much and she talked about the medicine and when it was brought I refused to take the medicine because “baya” praziquantel makes a lot of sounds in people.

**Mod;** so she had a negative respond

**F2;** why

**P2;** because it makes people to over frequent toilet.

**F2;** yes Sunday

**P5;** in our family we have discussed it, and the respond was positive. There was a time when they were distributing drugs my father collected our children to the Local council place for medication and they swallowed.

**F2;** on the day when they were not distributing the drugs, have you ever discussed?

**P5;** we discussed and he stopped those kids who used to go down there bathing and swimming, they stopped but these days my dad is never at home and our mum is the only at home and she does not talk, these kids escape to go swimming unless when am home I give them my command not to go anywhere and when they go I will beat them, they fear. But now as am here I will get them there swimming.

**F2;** so their opinion is like they listen today and the next day they are swimming

**P5;** yes.

**F2;** should we say they are adamant or stubborn,

**Mod;** but again these are children, they are easily taken up by peers, should we say that they are forgetful or what should we say … (laugh…)

**F2;** so you talk to them and they listen but because of that cohesion you find they go and do the same thing they have been stopped to do.

**F1;** let’s say they are forgetful, (laughing….)

**F2;** **who is most important is deciding if a family member comes in contact with schistosoma mansoni infected waters or receives praziquantel for treatment of schistosomiasis? Why do you think that person is important?**

**P5;** like for us in our family is our mother who takes that decision and she the one who takes care of us.

**F2;** why

**P5**; because she is the eldest in the family and any decision she is the one to take.

**P2;** at our home no one has ever discussed that.

**F2;** the mum that makes decision. For fortunate the mum that makes the decision on who to take and who should not the medicine.

**F2;** yes Sunday.

**P5**; my dad,

**F2;** why

**P5**; because he loves cleanliness and being healthy.

**Mod;** you said he does not stay around, does it means that he makes the decision from where he is?

**P5;** he used to stay around.

**F2;** so he is the one who makes the decision

**F2;** yes Olul,

**P4**; I said both parents

**F2**; why both parents.

**P4;** of course that’s their compound.

**F2;** ok, alright then.

**F2; who do you think should be given praziquantel?**

**P3;** according to me children from age of five and above.

**P2;** someone having bilharzia

**F2;** those who are infected with bilharzia

**F2;** fortunate you wanted to say something.

**P3;** the fishermen

**P2;** children age of 5 above

**F2;** children from...

**P2;** no I meant children ranging from age bracket from five and above

**F2;** all these one should be given

**F2;** Rovia, you wanted to say something.

**P1;** the snail miners

**F1, F2, All participants;** snail miners (laughing…)

**F2;** Olul,

**P4;** yes please, for me I would say children from five and above and they should do some examination for them.

**F2;** so you need examination also and all those who have been tested should be given.

**F2; so who should not be given praziquantel?**

**P2;** kids from four years below.

**F2;** kids four below.

**P4;** I think somebody who has been tested and found negative should not be given.

**F2;** those who have been tested and found negative and add children below five years should not be given.

**F2; Are there any reasons why a person should not take or not be given praziquantel?**

**P1;** there is no any reason, everyone has to take and the reason is to reduce the spread of the diseases.

**F2;** the reason is to prevent the spread.

**F1;** the prevalence rate

**P4;** once a person takes the medicine, the drug will stop the spread of the disease

**Mod;** now depends on the definitions of prevalence

**F2;** prevalence is more of eeh, it’s also still on the spread.

**F2;** prevalence is more of the new case being added on the odd cases.

**F1;** if you look at the population sample the number is added

**F2;** the prevalence is more of the new cases

**Mod;** so her reason is looking at the stop of the prevalence rate and at the same time the spread because if you take the medicine today you get protected and even if I pass stool here no one will get problem here.

**F1;** in terms of the infected people or the strength of the disease

**Mod;** so one can be stopping of the complications, when you take the medicine may be at the embryonic stage, it will kill them then your liver will not get affected and then when you talk about the spread it will be at the community.

**F2; so is there any other reason why your family should take “baya”, “Ambila” or praziquantel?**

**F2;** yes,

**P2;** mine is prevention is better than cure, so I should take before getting infected.

**F2;** infection is better than cure before getting infected.

**F2;** Are there any reasons why you or your family members or community should one take praziquantel?

**P3;** pregnant mothers should not take because the medicine may cause abortion or miscarriages.

**F2;** the pregnant women should not.

**F2;** yes Sunday

**P5;** children under five years should not take because I heard the drug is so strong for them.

**F2;** ok Olul,

**P4;** breast feeding mothers more especially when the child is still below six months

**F2;** ok, why?

**P4**; because of the reactions of the drugs to the mother and the baby. When the reaction start you will get disorganize because all the time you will be going to the latrine and the child will be crying and not being attended to may cause that disorganization.

**F2; Access to medications like antimalarial drugs and drugs like praziquantel might be a problem,**

**If it is a problem to you or your family, what are the reasons for this problem?**

**P3;** it is hard because you may go to the hospital and you will not get the medicine because its out of stock.

**P5;** I don’t think it’s a problem because if the medicine is not there, you can go and buy from the clinics.

I had my cousin sister, she was sick and taken to the health center, and there was no drug and later referred to Pakwach, and got drug from Dr.Ajal and she was treated from.

**F2;** what if you don’t have the money?

**P5;** that is when it will become a problem

**F2; Do you think being a man or a woman would make a difference in you or your family accessing praziquantel or using praziquantel?**

**F1;** is it easier for you to get this medicine for you being a lady?

**P3;** me being a lady?

**F2:** Yes, you being a lady does it make a difference in you or your family accessing praziquantel or “baya”?

**P3;** I don’t think it’s hard because they give medicine to LC and I will go and get the medicine from my Local Council (LC).

**F2;** going to LC, as a lady does it make any differences in getting the medicine.

**Mod;** may be let look at it this way, praziquantel is not given to the LC and you have to get it from the health center, we are looking at gender do you think you being a lady can make you get the drug easily than a man or there would be some segregation?

**F2;** anything that cannot make you get that medicine

**F2;** Rovia,

**P1;** for me it’s hard even if it has been put at every villages I will miss it because most time am here working and I don’t have time to move and will always hear that medicines has been given to people.

**F2;** because of you not being there at home, it makes access also hard.

**P1;** yes

**Mod;** is it because you are a woman or lady?

**P1**; a lady

**Mod;** that’s why you are not getting it or your work related issues?

**P1;** because of my work.

**Mod;** but otherwise you being a lady….

**F1;** but this information about these drugs are available over the radios and you as a woman you are in the kitchen, in the garden so cannot get the information.

**Mod;** so their work related issues make them miss out on some of these informations.

**F2;** miss out on some of these important informations but on the other hand it’s of an advantage to them and also disadvantages like for her she is specifically misses the information or get the information late because of her work and for the other women who remains who get the information.

**Mod;** so the work related issues and you as a woman do you have time to go and access these drugs?

**P2;** for my case normally get the information but I don’t go to get the medicine.

**F2;** why

**P2;** because I fear drugs.

**F2;** is it because you are a lady?

**P2;** may be the lady in me

**P2, F1, F2** ;( laughing…)

**P3;** there is some segregations in the health centers also as sometimes you would go for medications and the staff keep looking at you or may tell you am busy and yet she is not busy but when a male or man comes and tries to raise his voice this man will be given medicines

**F2;** so because you are a lady they just segregate you.

**P3;** because you may not have that voice that you may speak to.

**F1;** do you think it will be the same if it’s a man giving out drugs not a lady?

**P3;** but it depends on the person you get, because at times it’s the female doing this to the male so it makes it hard for you to access medicines.

**F2;** so you may reach and find a female who may not treat you a lady in the same way as she will treats the male.

**P3;** exactly.

**F2;** ok, Olul.

**P4;**of course this drugs are always sent to the community and the information is always passed and on hearing about the information ,I take my children or family and we get the medicine and I have never missed drugs.

Whenever I need drugs I just go to the health centers and they are given to me.

**F2;** when you go to the health centers, why do you think you are given always?

**P4;** because I explain myself why I have missed the medicines.

**F2;** and do you think if your husband is to go and get the medicine, he will be accorded the same treatment?

**P4;** yes.

**F2;** so I don’t have much to say now, we have finished our questions may be if you have some questions you are free to ask us.

**F2**; so you are the only group that I have managed to master all your names, Rovia,Lillian,Fortunate,Sunday and Florence Olul and don’t ask me why. (Laughing...)

**F2;** thank once again for your participation and your time have a good day.
