# Supplementary material for: Gendered lives, gendered Vulnerabilities: An intersectional gender analysis of exposure to and treatment of schistosomiasis in Pakwach district, Uganda
Source: PLoS Negl Trop Dis. 2023 Nov 10;17(11):e0010639. doi: 10.1371/journal.pntd.0010639 (PMC10684070; doi:10.1371/journal.pntd.0010639)
Supplement: S1 Data — (ZIP) [file pntd.0010639.s001.zip › FGD Schisto Interviews/FGD 46-65 FEMALE PANYIMUR.docx]

**GENDER INTERSECTIONALITY**

**AND**

**SCHISTOSOMIASIS IN RURAL UGANDA**

**TRANSCRIPTION FOR FOCUSED GROUP DISCUSSION.**

# Abbreviations and acronyms

**FGD** – Focus Group Discussion

**GP2** –Group Two.

**F1**-Facilitator 1

**F2**-Facilitator 2

**Mod**-Moderator

**P1**-Participant 1

**P2**-Participant 2

**P3**-Participant 3

**P4**-Participant 4

**P5**-Participant 5

**GP2. FEMALE (46-65) FGD**

**Introduction;**

**F2;** Good afternoon to you all and thanks for coming and you are group number one, though we had started with second group of the men but still you are group number one (1) of age bracket from 46-65 years. As we are going to continue I would like us to introduce ourselves, you will tell us your name, the village where you come from, so we shall start from here.

**F2;** we can introduce ourselves.

**P8;** my name is Betty

**F2;** you raise up your voice a bit.

**P8;** my name is Betty Candiru form mukindwa village

**F2;** Betty candiru from mukindwa village

**F2;** mama

**P2;** Jerech Nyapamba from Abiia village

**P3;** Oduru Grace from singilla central

**P4;** my name is sereptah opai from singilla A

**F2;** you remove the masks a bit

**F2**; yes.

**P5;** my name is atimango Alice from singilla central south

**P6;** my name is Magdalene pacutho from singilla B.

**F2;** among us there is a light form we had talk about it to allow us have this gathering that you have agreed to be asked some questions by us.

**F2;** so these papers are in both English and Alur languages. if you need the one in English you can make a mention of the form to be given to you and you will sign down here ,have we all agreed to participate in our discussion.

**F2;** which one do you want to raed,English or Alur language.so this forms are consent forms in clinic.

**P4;** give me the one for alur language.

**F2;**as we had said that we are going to answer different questions and what you are going to answer will help in our studies to see the issues of bilharzia in our communities which involves women and men ,how the medicines is being used or how we can controlled this Bilharzia in women and men.

Secondly when we are to look at present state of bilharzia here in Pakwach, bilharzia is a big problem and some people has called it “Awola” a local disease that affects the abdominal, causes distension/swelling and diarrhea.

In the whole of Uganda it has been found out that three(3) out of seven do have bilharzia and when we are to look at our Pakwach ,let’s take Panyimur, it has been found out that five (5) out of ten meaning that minimum of 5 five people are having bilharzia.

**F2;** If am to take just last week children near Nyakagei primary school were tested up there, people from the singilla south could be knowing about this, Nyakagei 108 children were tested all of them had bilharzia, let’s take like that in kivujje 104 were tested, only 2 were not having the worms, but the rest of 102 had bilharzia, so ours here what they say 5/10 here, it is not there. It is 10/10 are having the bilharzia disease; you can imagine the magnitude of the problem.

And out of these ten people if they are to be given praziquantel, only seven can get healed and the 3 three will not get cured.

So this day we have been giving the drugs to the children at school and the drug is not for selling, it is the government to supply this drugs because it is expensive to buy.so the children in primary and in secondary so it’s not easy to get this drug.so we are going to see between women and men, this drug how can we use it in order to prevent this disease.

**F2;**so we are going to give questions, and these questions when one person is answering ,the others have to listen and secondly you will remove the mask a bit so that our voices can be heard. What has been put in the middle here is to record our voices but others are being written down.

**F2;** so for me am Noah Okumu and the other person writing is Phillip, Ocama Peter and Nakiranda Salama are my colleagues in this meeting

We have a total of twelve (12) of them exactly and all of us I think will answer one by one. Your thoughts/suggestion or you will give us what you see, how you see them or your experiences.

**F2; so our first question is what activities do you or your family or relatives perform that might lead to infection with schistosomiasis?**

**F2;** yes mamma,

**P10;**bilharzia like us mothers with children and even us alone, those days they used to fetch river water and in that water they normally say when you enter in the water, it enters under the hair and you get the disease.

Coming to the children, me as the mother tested and they found out that I have bilharzia ,I tried to stopped him telling him he will get bilharzia as long as he continues to bathe in the river but he could not listen as child born next to the river, he would go there to bathe and these bilharzia very early morning they are down under water and once the sun rises they come up on top of the water and these children like bathing during the afternoon hours and late evenings and that’s the time bilharzia enters their bodies.

**F2;** so there are two points she talked about, first fetching of water and secondly bathing or we called it swimming in water by children.

**F2;** so what other reasons do we do at home that make us get bilharzia.

**P9;**we women as we go to fetch water sometimes end up bathing in the river during the day when the bilharzia is already on the surface of water can also make them to get bilharzia I have stopped there.

**F2;** yes, no.8

**P8;** I think about bilharzia, am going to talk about these people who go fishing at the lake, they can swim to get into the boat and after that they still drink this water. They said this bilharzia normally passes from those who do open defecation near the water bodies or in the water were it is washed away into the river by rain and gets into water snails and later enters the body of a person who enters this water.

**F2;** so we have fishing and the next thing is defecation in the lake.

**F2;** fishing and defecating in water, swimming and others...

**F2;** yes.

**P5;**my name is Atimango Alice from singilla south, bilharzia we get it through washing of clothes as well because you will be in dirty water for a long time and you can get it there.

**P5**; then half cooking of fish can also make us to get bilharzia.

**F2;** any other suggestions.

**F2; why are men more likely to be infected than women in some communities?**

**P5;** men take longer time at river bank than women; like fishing are done by men and digging near the lake are also done by them.

**F2;** so digging near the lake is also done by them,

**P5;** yes.

**F2;** what else

**P3;** am oduru grace, swimming in the water men like swimming in the water since the activity is done in the lake this exposes them more than women.

**F2;** swimming,

**F2;** what are things do you think makes men at high risk of getting this disease than women?

**P5;**am thinking of drinking of alcohol makes some men not to take care of themselves and more so when we are distributing drugs given by the government they don’t take because most time you find they are drunk.

**F2;** ok, another one.

**F2; why are women or their children more likely to be infected in some communities?**

**P5**; women uses river water more than men. They fetch the water; use it for washing of clothes and for cooking.

**F2;** Aaah, she said women uses river water more than men. Any other suggestion to add onto that?

**P8;** I think, if you see the family itself its most of the time women who stay with children, they are the one who produce the children, care for them as the women stay with the children at home you will find that the child will go around defecating and you as the mother if you are not careful the feaces can be washed to the river and you will go fetch the water for home use by the children themselves. I think that is another way of getting the disease.

**F2;** so women are more exposed to bilharzia because they are the one who normally fetch water from the river.

**P5;** yes, we are the one.

**F2;** any other activities that women normally do at the river banks leaving fetching water, washing clothes,

**P2;**sometimes back I used to scoop silver fish from the lake and would stay in the water for long time, would sometimes use bare hand to remove water snails away from the basen,such activities would lead to infection by these worms.

**F2;** Mamma, you want to say something?

**P4;**I want to say something about the nature how women cook where the food /fish is not ready and whole family will eat that half cook fish and they will all have the disease.

**F2;** yes,

**P5;**other point is this business of snail mining, women from here are involved in snail mining where they enter in deeper dirty water to scoop these snails with basin , this exposes them to get bilharzia.

**F2;** women do snails mining, ok.

**F2; what changes in lifestyle can you or your family make to prevent you from getting schistosomiasis?**

**P4;**the lifestyle changes that can make me or my family to prevent us from getting bilharzia is this medicines that are always brought to us to take, you will some people refuse to take these medicines because of ignorance.

Then you hear my son is urinating blood, my son is having abdominal distension and cannot breath well referring all these happenings to witch crafts, not knowing that when you bilharzia you can urinate blood, defecate blood, vomit blood and abdominal swelling as well all these because of lack of knowledge until when they are taken to the hospital like our big hospital is Angal from there they will test to find that they are having bilharzia disease not witch craft, they may get treated or die because of wasting time because of ignorance.

**F2;** she is talking about lack of knowledge.

**F1;** yes, not life style changes.

**F2;** so, what changes in lifestyle can you or your family make to prevent you from getting schistosomiasis?

**P4;** to change our lifestyles, first we should make sure that these medicines when given to us we must take it and swallow them as per instructions given to us.

**F2;** what other changes, I start from here now...

**P5;** secondly, am thinking that after fetching water we must boil the water for drinking and after taking treatment of bilharzia we must leave the activities that led us into getting the disease

**F2;** what are the activities you are talking about.

**P5;** the activities like prolong staying in water when washing clothes, snails mining and bathing in the river we must stop them.

**F2;** what else are you giving us?

**All participants, F1, F2 ;(** laughing…..)

**P8;** it’s ok, according to me, you know bilharzia is already there at a place so to change ourselves not to be in it again or complete it if at all people would follow the drugs which the government has always been giving and those who are affected could be taking the dose and those who cured could cure and after wards we stop may be, people could stop swimming, people could stop fishing and if at all like fishing is the nature of people in the lake sore and it is really their work and it cannot be changed and if the government could change a system where a fishermen can go and be protected from bilharzia at that very time and as well as coming back. **P8;** For those who are around streams, now it’s known that they are brought by the government, when I was small I used to know that Tap water are in Arua only but as I grew up found out that tap water and streams water were people could go, if only people can decides to only use tap water, if they are to use the lake they should boil it or leave the water in the sun for bathing and other home use.

**F2;**thank you she has said a lot of points, first is that bilharzia is among us ,secondly fishing you can say people should stop fishing because our lives depend on the river and if the government can do something that can protect these people going to fish would be good and she also said that we normally fetch water from the lake ,and if the government can bring enough tap water to people so that people don’t go the lake so much to fetch water and that would reduce the strength of getting this disease in our body.

**F2;** any other things.

**P8**; I was saying sun heating of water and boiling

**F2;**boiling of water in the community would be good and this answer this question number five also which talks about changes community or health system or local government would help to control bilharzia. So we shall go straight to the question and answer it from where she has stopped.

**F2; what changes in your community or health systems or local government would help control or eradicate schistosomiasis from your community?**

**P5;** am suggesting that the government should have routine screening of people for bilharzia.

**F2;** routine screening.

**F2;** yes,

**P8;** you know the screening goes with the medication if found positive.

**F2;** another one,

**P4;** the government should provide health education to the community through Village Health Teams, like Alice here with us.

**F2;** community sensitization

**F2;** what are you telling us...

**P2;** for me I don’t have much to say but these people we normally lined up for them, they go and don’t come back to check how we are doing is what pains me.

**F2;** yes mum, I understand your point but let us not mix the lining and the healthy living together otherwise we shall be confused.

**P2;** it’s fine; I told you that am old I just mix anything anyhow.

**All participants, F1, F2 ;(** laughing…)

**P4;** like these people who have been placed to visit homes, but first of all we the owners of the homes or head of families must ensure that there is a pit latrine at home. Secondly the compound should keep clean, rubbish pit must be there and these things will help to bring some cleanliness at home. And stopping children to go the lake.

**F2;** regulations

**P1;** I have the same suggestions.

**P5;** for me am suggesting that we should teach our children how to wash hands after visiting latrines.

**F2;** promoting sanitation and hand hygiene.

**P8;** that’s sanitation and hygiene most of the time within the family itself but also thinking that if the government could also control open defecation along the lake areas that is very important according to me

**F1;** how can they control that?

**P5;** by building**…**

**P8;** building of something, posters, to say pit latrines and fining of people caught doing open defecation but again it will be hard to get those doing it at night.

**F2;**so, are you listening she has been giving her opinion and saying that if the government could build pit latrines and put some posters that people should not defecate in the open and fining people caught defecating in the open but sometimes it hard like at night do know what some body is doing?

**All participants;** we do not know

**F2;** so those were are suggestions.

**F2;** yes, what else are you having...

**P2;** cracking out a joke...

**F2, P2, and some participants ;(** laughing…)

**F2;** ok, we are talking about our own family, our experience (expera)

**F2; has your family ever discussed use of praziquantel or any ways to prevent schistosomiasis? If they have what are their opinions?**

**P4;** for me I stay with my grandchildren, but our pit latrine was destroyed by water and I said for how long shall we be begging people’s pit latrine? So decided to put this boys down so that we can discuss way forward of building a new pit latrine, I told them we have that land up there which we bought some times back, you go and build a pit latrine for us, we shall be going there and built placed water there for washing hands. And now we are fine.

**F2; so why did you build the latrine there?**

**P4;** the reason why we built that latrine was that I thought of peoples latrines tend not to have cleanliness, as they defecate at the entrance, at the holes and some people put fecal matters on the wall and feel so bad about it that’s why I told them if you refused to build latrine I will go back to my people at our home and leave you people because I don’t want to beg for pit latrine. They built a latrine to avoid getting diseases.

**F2;**I wanted to listen to the main reasons as to why you built the latrine because some people don’t know why they have to build a latrine, or may build it and refuse to use it.

**P4;** they thanked me for telling them ….

**F2;** so, what are some of the disease

**P4;** I told them some of the worms that people get, like even HIV/AIDS, bilharzia as some body with bilharzia may defecate at the entrance and you can step on it and get the disease and more so we old people squatting is also a problem and you would wish to have a chair to sit on. You may even touch the one on the wall as well, even if you wash your hands, some may pass and you get the disease.

**All participants, Mod, F1, and F2;** (laughing….)

**P5;** with what I have seen how bilharzia disturb people made me to be having discussion about bilharzia, and it’s not once but many times. We don’t go to the river to fetch water, we use tap water and sometimes you may find the water has disappeared for some days so bought drums and keep filling whenever there is water to help us when the water has gone.

And once they are distributing drugs, I make sure people take the medicines, though some people may refuse to take but I forced them to take.

**F2;** so if it comes to taking of medicines, there’s mixed reactions, opinions.

**F2;** why do they refuse to take the medicine?

**P4**; they say there is no worms in them and how can we take medicines without testing us? I would tell them you take the medicine.

**F2;** that’s another reason

**F2;** yes Mamma.

**P4;**for me am here I have tried to raise my children and they are all big men and women with their families, where I don’t talk about their families but there is this one here ,my grandchildren loves me is always there, so I have some of them living with me. From the time borehole was brought next to my home, we stopped using the water from the lake.

And you know children, normally escape to go and swim with their friends and once they are back I would put them down and tell them never to go back again to the lake, they will get bilharzia and give them some strokes.

Secondly, at home the most important things as said before; rubbish pit, drying line, drying rakes, latrines and bathing shelters these are things used for cleanliness of the homes.

Sweeping of compound, removing of feaces after children have defecated in the compound and pouring them to pit latrines.

**P4;** they should wash their hands after visiting the latrines and even before Corona because the dirt in the latrines is totally different.

Another thing is telling these children to take medicines whenever they are given from schools. It’s very true many children don’t want to take medicines but you as a parent must make sure that they take the medicines.

For examples, I had some two children who disturbed me a lot, I used to get medicines from Charity, they had worms but now they have improved.

**F2;** thank you. Finally you and we go to the next question.

**P3;** as a family if you are staying with your children even if with your husband what you have to tell them is that there has to be a latrine for the family home affair first of all you have to show them that the latrine is there not to have open defecation, where ever they go they should be using the latrines so as we can be having that control of Bilharzia.

**F2;** thank you. Where we have not been talking, let us start talking. For they say home hygiene/cleanliness starts with you. Cleanliness is made at home but repaired in the hospitals.

**F2;** so the next question is saying;

**F2; who is the most important in deciding if a family member comes in contact with schistosoma mansoni infected waters or receives praziquantel for treatment of schistosomiasis? Why do you think that person is important?**

**F2;** that one is another experience; we shall start from Mummy here.

**All participants ;(** laughing…)

**P4;** Aaah, if you are all alive, a man is supposed to be the one to talk but if he is dead, a woman takes over and you on behave of the man and woman all. You have to give clear directives that can enter in these children.

Like for me I have been alone for 25 years in my family…

**F2;** why do you think that a man is the one to talk?

**P4**; why am saying that a man should be the one to talk is not that he will talk alone but the woman will also be around as you talk to your family and through that fear will enter into the children as well. They will see that our father and mother have all talked about the same thing ,I think this thing they are talking about is really bad.

**F2;** yes.

**P5;** for me in my family issue pertaining health, I talk more than my husband.

**F2 ;(** laughing…) why?

**P5;**because if the children are sick ,I will be the one to go and take care of them in the hospital ,he will be only be visiting us as for me I have to stay there.

**F2;** so you talk more than your husband is because if the children are sick you are the one to take care of them in the hospital as he only come to visit.

**P5;** yes.

**F2;** yes

**P6**;for me ,in a family the man talks more but the woman stay more at home with the children than the men so that gives you more opportunity to talk because most times he will not be there at home.

**F2;** to say taking up the decision is also you, like pertaining taking medicine for bilharzia?

**P5, p6;** yes, because he moves a lot.

**P5, p6, p8 ;(** laughing…)

**P6;** he does not even stay with the children.

**P8;** according to the nature, it used to be said that a man is the head of the home (laughing...)But now if you are to see the health, a woman can be the head of the home now according to the health. If not the woman or the man, any other child who knows more about the health can take over. Someone who knows more about health and the betterment of health can even be...

**F2;**But according to you who makes important decision, if a family member comes into contact with bilharzia or is to receive the treatment of bilharzia? Whose decision is more important?

**P8;** me the woman.

**F2;** you the woman...

**P8**; yes

**F2;** why

**P8;** because am to fetch water, boil and use it with whole family.

**F2;** Ok.so because you fetch, boil and use it with whole family you decide so and so are to take medicine, and who should not go to the water.

**P8;** yes.

**F2;** for you, who do you think should decide?

**P2;**for me I found out that in a family who suffers with children a lot when they are sick and are always loaded with a lot of work. The men are never home and the worst thing is they normally claim that children are theirs and I have found out that they are high jerkers.

**All participants** ;( laughing….)

**P2;** and if there is one child who is sick, he will not even know but he will be defending that the child is his. And sending you away, telling you to leave his child but for me I cannot leave my child as long as he has not broken my leg.

**F2;** he should not break your leg

**P2;** yes.

**F2;** so who takes the decision to prevent bilharzia in the family?

**P1;** at home, it’s the man head of the family.

**F2;** why do you say his voice is the most important?

**P1;** because he is the one who brought me (married me).

**All participants, F2 ;(** laughing…)

**F2;** so the next question number 8.

**F2; who should be given the praziquantel?**

**P8;** all of us .those who are affected and all of us who stay around lake areas or around the streams whereby can cause someone’s life.

**F2;** so who should take the medicine? Mama,

**P2;** she has answered it.

**F2, other participants ;(** laughing…)

**F2; so who should not take or not be given praziquantel?**

**P2;** drug for bilharzia?

**F2;** yes

**P2;** Aaah, there is a time I took it, I almost died.

**All participants ;(** laughing…)

**F2;** to say you are thinking of not taking?

**P2;** that drug for bilharzia it’s not easy.

**F2;** who do you think should not take?

**P4;** we must all take. The children and elders all must take to be healthy.

**P5;** for me I think that those who are pregnant should not take.

**F2**; those who are pregnant should not take, why?

**P5;** it may killed the unborn or may cause abortion and miscarriages.

**F2;** ok

**P5;** then children under five years should not take because they are still weak.

**F2;** okay other person who should not take.

**P2;** they should first test.

**F2;** so you have said those who are pregnant and young children under five years should not take.

**F1;** she has said some point there, those who tested positive.

Mod; those who are tested negative should not take.

**F2;** you are suggesting that those who should take have to be tested first, and if found positive should take and who are negative should not take.

**P2;** we can still take it,

**F2;** that was your thought

**P2;** yes, but am not above the law. You can give me now and I take it but the other time it almost killed.

**F2;** so you are suggesting that, they should have tested you first before being given the medicines.

**F2;** (laughing…)

**F1;** it’s not for bad. Don’t worry.

**F2; Are there any reasons why you or your family members or community should one take praziquantel?**

**F2;** yes

**P5;** for me, am thinking that they should take.

**P2;** because the areas we are living in is bad.

**F2;** ok, why is it bad?

**P5;** it is bad because we are near the lake sores, the behaviors of people.

**F2;** How do they behave?

**P5;** drinking of alcohol, open defecation and urinating near or in the lake and the children play and mix themselves anyhow those are some of the reasons to make us take the medicines.

**P2;** for alcohol, some people after taking it they become real ad.

**All participants;** (laughing……)

**F2;** ayah….any other reason to give?

**P8;** yes, because of the fishing

**F2;** ok, because of the fishing activities

**F2; Are there any reasons why a person should not take or not be given praziquantel?**

**F2**; yes mummy,

**P2;** that is now by gone and it has passed. For me, I was just whispering to myself, but I don’t what is wrong with my voice. I had said for the medicine almost killed me and for that I will never take it again.

**F2;** why

**P2;** because it wanted to kill me.

**F2**; yes.

**P8;** according to me…

**F2;** yes the side effects.

**F2;** so you are thinking that before testing any one…

**P2;** not somebody, but me myself should be tested first before giving me the medicine.

**F2;** no, you are giving an example which can help others as well.

**P2;** I have left it now...

**F2;** so you were saying before they test you…

**F2;** yes

**P8;** now when the drug is there, it brings side effects along, is it because of school children normally take it before eating. If it’s taken at school you find that reaching lunch hour, every child would rush home defecating diarrhea, diarrhea and even vomiting many times so community would begin complaining blaming the government so that one alone can bring the image of not being taken.

**F2;** so you think those who are hungry should not take.

**P8;** yes.

**F1;** a person who has not eaten should not take the medicine.

**F2;** yes

**P5;** am thinking of those who are severely ill should not take

**F2;** any other person whom you think should not take.

**P2**; why are you repeating my case.

**F2**; she said those who are hungry or have not eaten anything and those who ill should not take.

**P2;** that is why I almost died

**Participants** ;( laughing…)

**F2;** ok, we are remaining with two questions, one of it is saying….

**F2; Access to medications like antimalarial drugs and drugs like praziquantel might be a problem. If it is a problem to you or your family, what are the reasons for this problem?**

**P2;** the problem for me I see …

**F2;** you first show that you’re going to talk.

**P2** ;( she laughs…) for me I had started...

**F2;** yes

**P5;** for me I the problem is lack of money.

**F2;** lack of money, why lack of money?

**P5;** lack of money because sometimes you are sick giving example of malaria, you don’t sleep under mosquito net and don’t have money to buy the net. And there is no way to go to the government hospitals, you may go to the health centers and you will not get the medicines, there is no money to go and buy. So am suggesting that if the government can bring or supply enough drugs to these health centers it would be good.

**F2;** okay other suggestions

**P2;** taking this medicine needs you to be having something to eat, or should be having some money to buy what to eat and there is no money. Sometimes you can stay hungry for about three days without eating anything. I have eaten something now am feeling drowsy as if I have eaten poison.

**All participants;** (laughing…)

**F2;** first wait, we are now talking about the access or getting of the medicine but if the medicine is there, no problem but we are talking about getting the medicine just like she was saying that sometimes you may go to the health center and may fail to get the medicine, there is no money to buy the medicines such kind of things …

**P2;** ok

**F2;** yes mama,

**P4;** am just saying the same thing that lack of money is the main problem because when you are sick, you will go to the health centers reaching there, they will tell there is no medicine go and buy from the clinics and yet you don’t have money so will return back home to wait for your death. So the only way out is for the government to supply enough medicine for us, and that would be very good.

**P2;**about the medicine am still saying, when you go to get the medicines from the health centers they will tell you there is no medicine go and buy from the clinic with your own money and if you don’t have the money you will not take the medicine.

**F2;** increase in stock of medicines.

**F2;** yes, you have something to say.

**P8;** I was saying like in the hospital when people go, maybe there is big number of people there at the hospital and the number of the health workers are few so you will find another point reaching to a patient who is in bad condition, it will take time though the drugs may be there or they will say go and buy from outside you will find reaching the patient very quickly will be hard because of the numbers of the workers.

**F2;** what will happen when failed to reach the patient?

**P8;** death occurs if the condition is bad.

**F2**; how does it affects its access

**F1;** the access

**Mod;** the population makes some people to fail to access the drugs because of the long queue, makes them to give up and go home to meet their death.

**P8;** even the health workers takes long time to come and administer medicines to help the one who is ….

**F2, F1;** high work load on the side of the health workers

**Mod;** even if you try, you will find that you cannot meet the demands of every one

**P8;** some health workers have bad attitudes towards patients, they despises them.

**F1;** so then attitudes

**F2;** ok, the last questions is back to us.

**F2; Do you think being a man or a woman would make a difference in you or your family accessing praziquantel or using praziquantel?**

**F2;** I will start from here;

**P1;** if they have brought medicines, I have to give to my families.

**F2;** do you think there is advantages of being a woman?

**P1;** yes, there is advantages of being a woman, because women think a lot of those small, small things than men in the family.

**F2;** so women think so much of the integrities, they take charge of details.

**F2 ;(** laughs….), ok mama

**P8;** of course, if you are a woman you can advise your children and their father to take medicine for bilharzia and if you have taken medicine as a family you will become better person, healthy, strong to work and get money so can be free from bilharzia.

**F2;** so you have advantages because you can easily guide, advise and they can listen to you because of that.

**F2;** am trying to get what you were saying

**Mod**; your presence one.

**F2;** so, what of you…as a mother

**P4;**as a women, a woman can helps a lot more because you are the one to go and buy the medicines when he has gone to move and he is not around at home and it is you the pillar to that family and advises the children.

**F2;** ok, yes

**P5**; for me as a woman, I think I have a lot of time to advise these children because their father is never at home and I have a lot of time to teach them what can bring improvement in our lives because if there is no any sickness like bilharzia in a family there will be development at home.

**F2;** ok

**F2;** what are you thinking….

**P6;** advantages of a mother is there, the advantages are to both the children and their father as well because you may have a husband who does care about sickness or anything but you as a mother you will sit down and plan let’s do this like this, are you not seeing this sickness at home and if he can listen, you will collect everything and even help the children and even among yourselves

**F2;** thank you so much, we have finished our last question, if you have any question you are free to ask.

**F2;** yes

**P6;** I want to ask on this bilharzia, how many doses do you take to get cured?

**F2;**I had answered that question before, we were saying this medicine for bilharzia you can take it for the first time, second time depending on the level of worms infestation. This worm for bilharzia can stay in our bodies for fifteen years to say if you have taken this medicine, and you have taken again like Pakwach has taken this medicine for sixteen years starting from 2004 up to date 2020, it was launched at Omach playground. They had started taking this medicine since then, sometimes they take it twice a year why because those worms are still there and when you take the medicine, it reduces its strength and the level of the worms but at most times we do go back again to the water, to say if they are still testing and finding the worms there, like right now it was tested and found out that its spread has risen two times more than what it was before. And if people were following the regulations and laws made, it would have been easy to know that, I will take this medicine for two or three times and I will be cured.so right now we shall continue to take this medicine because our lifestyles has not changed.

**F1;** there is another hand

**F2;** have I answered you?

**P8;** yes. But now someone has got the bilharzia and here the government normally brings the medicines is it after every two years?

**F1;** one year

**P8;** and how could it be better for some already showing signs and symptoms of bilharzia to wait for another year again to come for the treatment, that one there I have failed to understand?

**F2;** you know these drugs are never supplied like in the circle of national medical stores and they are donations. And these donations the government just receives from outside. The amount of money spend on the drug for one person is always highland most times are given by World Health Organization and that is why sometimes they target only students or school children because of the cost of the drugs and very good people sometimes they say treat the whole community involved.

We are also advocating for the health facilities to be having some to treat people who are already having the disease not only to wait for donation to give to people.

Apart from that I love to say thank you for you participation and for the one hour you have given us. I have handed you over to this people; we have another group coming after you.
