# Supplementary material for: Gendered lives, gendered Vulnerabilities: An intersectional gender analysis of exposure to and treatment of schistosomiasis in Pakwach district, Uganda
Source: PLoS Negl Trop Dis. 2023 Nov 10;17(11):e0010639. doi: 10.1371/journal.pntd.0010639 (PMC10684070; doi:10.1371/journal.pntd.0010639)
Supplement: S1 Data — (ZIP) [file pntd.0010639.s001.zip › FGD Schisto Interviews/FGD FEMALE 18-45 PKH..docx]

**GENDER INTERSECTIONALITY**

**AND**

**SCHISTOSOMIASIS IN RURAL UGANDA**

**TRANSCRIPTION AND TRANSLATION FOR FOCUSED GROUP DISCUSSION.**

# Abbreviations and acronyms

FGD – Focus Group Discussion

GP2 –Group Three.

F1-Facilitator 1

F2-Facilitator 2

Mod-Moderator

P1-Participant 1

P2-Participant 2

P3-Participant 3

P4-Participant 4

P5-Participant 5

**GP3. FEMALE (18-45) FGD**

**Introduction:**

**F2;** you are most welcome to this place. Which language do you want to use, or any language that comes in your mouth?

If somebody wants to express herself in English is ok we shall translate in alur.so we may jump from one language to the other for shake of clarity.

**All participants;** hmm,yes.

**F2;** so, we finished most of the things, you have your consent signed and you know why we are here.

These are our questionnaire we have twelve questions in total .You are group three of ladies or girls age brackets of (18-45) years. Group number three, we are going to ask you some few questions and we are choosing to have for each question at least five minutes. For we have to spend about five minutes for each question and whatever response you’re going to give will be confidential and it’s important for the studies and also beneficial to our communities.so even if you share your personal experience ,we shall not tell anyone outside but it will be good for us.

I hope we are good to go.

My name is Noah Okumu and is a facilitator for our discussion today and my colleagues are Phillip, Ocama Peter our Moderator, Nakiranda Salama is our Administrator. And you will tell us your names so that we can know each other Anyutha Christine a participant, Ayomirwoth Flavia, Oyungrwoth Patricia, Amia Phiona and Pimer Joan thank you all.

So am going to start with the first question and if you feel like answering you can make a sign or you can raised up your hand and then when you are speaking, adjust your mask so that you are audible. And if you find English is having some issues for you can explain in Alur.

**F2; what activities do you or your family or relatives perform that might lead to infection with (bilharzia) schistosomiasis?**

**F2;** we can start here

**P1;** bathing in the river.

**P2;** fishing

**F2;** fishing

**P3;** opened defecation near the river and also defecation in the river.

**F2;** yes

**P4;** playing in dirty water

**F2;** playing in the dirty water contaminated water.

**F2**; any other

**P5;** already mentioned

**P1;** defecation in the bushes

**F2;** those are the opened defecation

**F2;** what other activities that they do are not there

**P1;** drinking unboiled water

**F2;** where do buy fish from?

**All Participants;** from river bank

**F2;** we don’t have those who sell onanang

**P4;** washing of clothes

**P3**; washing of clothes at Koppio River.

**Mod;** washing of clothes, how does washing of clothes make you get bilharzia?

**Mod;** so we are trying to look at question number one, if we try to pounds it more and try to throw more light so when you try to explain it to somebody ,he can understand better.

**Admin;** you see when you keep mentioning the activities like it would look like quantitative and yet this is qualitative where it needs more explanations.

**F2;** like what the elders were doing

**F2;** so we are starting from washing of clothes, and how does it lead to infections with bilharzia.

**P3;** carry your clothes to the river side and collect water to wash the clothes on the hilly side leaving the water away, but wanted to ask question, is like when you have collected water in the basin and you are washing from raised area do you get the disease?

**F2;** but you first enter into that water, you fetch and get out?

**P3;** yes

**F2;** when you had said that you fetch water, and how do fetch this water?

**P1;** we enter into the water**.**

**F2;** how deep do you enter into the water

**P1;** up to the knee level.

**F2;** up to the knee then you fetch?

**P2;** hmm.

**F2;** then you get out .and at what time of the day do you fetch this water?

**P1;** at any time. You may go in the morning, after lunch or in the evening.

**F2;** now when you washing clothes in Mubogo, do you pull the water with tap?

**F1, F2, P2, P3, P4, Mod ;(** laughers…..)

**P1;** you enter into the water; fetch and then you move out to wash from a dry place.

**F2;** where do you rinse from?

**P1;** you can rinse from outside where you fetch water and come out with it to rinse or you can rinse from the river direct.

**F2;** but you keep entering the water/

**P1;** yes.

**F2;** ok, we are asking the second question now,

**F2; why are men more likely to be infected than women in some communities?**

**P3;** for the men you will find that most of their time ,they spend fishing and sometimes they defecate in the water and later start using the same they have been defecating in.And that’s why they are more likely to get the disease than women.

**F2;** they are always in the water, more exposed in fishing and they are the same people who contaminate the water.

**P3;** Hmm.

**P1;** yeah.

**F2;** yes another one why do you think men more likely to be infected than women in some communities?

**P1;** am talking about the young boys...

**F1;** you said,

**F2;** they don’t have time to first come back, clean up and go back… (Laughs...)

**P1;**Ok am talking about the young boys ,like right now koppio river is full, and go spend more time swimming than women who just go fetch water and come out immediately, so on that ground they can get disease.

**F2;** so the male who like playing and swimming in water mostly the boys and the girls go to fetch water only.

**F2;** yes. On the other hand

**F2; why are women or their children more likely to be infected in some communities?**

**F2;** yes

**P3;** if we are to take communities with no big water bodies, they don’t do fishing ,its only seasonal rivers and its only women going to fetch water and men don’t go there. So women are more likely to get the disease than the men.

**F1;** what is she saying?

**F2;** ok,

**Mod;** she is talking about seasonal rivers.

**P1;** in communities where there are seasonal rivers, there are no fishing taking place and it’s mostly women going to fetch water and men don’t go there. So women are more likely to get the disease than the men.

**F1;** women go to fetch water at most time than men.

**F2;** yes.

**P3;** for the sake of girls, you will find these young girls following older women to the river without these women knowing and they go the river to play in the water and that’s how they exposes them to the diseases.

**Mod;** so we are looking at the children following their mothers to the river and end up playing in the water and the end results they all get infected.

**F2;** there the young girls, older women and then they get exposed.

**Mod;** they get exposed, yeah.

**F2;** ok, any other

**F2;** is your husband there?

**P3**; No

**F2;** you stay alone,

**P3;** Yes

**F2;** If he was there I was going to ask you a question.

**F2, other participants;** (laughs…)

**F2;** oh yes,

**P1;** hold on, is like for the head of the family (husband) does not do fishing activities, you will find that it’s the woman who will be in contact with water at most times, right from fetching it to her home for washing utensils, cooking and bathing, this makes men less exposed.

**F2;** is like women are always burdened with the domestic work fetching it to her home for washing, cooking and bathing.

**P1;** bathing children, hmm

**P3;** and sometimes you find these women are overwhelmed with work and would get the water direct from river and use it bathe children.

**P1;** sometimes you find the woman and her children are bathing using cold water but the husband is given warm water to bath.

**F2, F1, P3, P1, & others;** (laughing …)

**F2;** that point has come...

**F2;** you find that most of the time the big man is honored with warm water while the woman and children bathe cold water.

**P1;** and they bathe during the day.

**P1, F1, F2 ;(** laughing…)

**F1;** so women and children normally use raw water. (Laughs….)

**Mod;** and they bathe during the day.

**P1;** and for the children, most cases in the evening when the sun set.

**F2;** are there any other reasons why are women or their children more likely to be infected in some communities?

**F2;** we can be reflecting back and see ,ok I have not being keen with this one, but now I know bilharzia is transmitted in this manner, these are some of the things I see.

**F2;** so I would ask if there is no other,

**F2; what changes in lifestyle can you or your family make to prevent you from getting schistosomiasis?**

**P1**; always defecating in the latrine.

**F2**; what will defecating in the latrine do?

**P1;** it will prevent people from dropping feaces anyhow. Later if it rains, it carries the eggs to the water bodies and later goes fetch and drink in process of infection.

**F2;** have you read now,

**P1;** Hmm

**P1;** the family should have tip tap to remove the germs.

**F2;** hand washing facilities.

**P3;** strict rules should be put in place so that

**F2;** before you go to strict rules, these are more of life style changes that you and family can do, what changes in lifestyle can you do or your family make to prevent you from getting schistosomiasis?

**P3;** ok, using treated water.

**F2;** treated water, how?

**P3;** ok, through boiling

**F2;** which water, the one in the lake or river?

**P3,** any for drinking**.**

**F2;** ok, what do think are the changes in lifestyle you or your family can make to preventing you from getting schistosomiasis?

**P3;** treatment of water using water guard.

**F2;** treatment of water with water guard.

**F2;** ok, what else?

**F2;** so one person talk of use of pit latrine, boiling of water and use water guards for treatment of water

**F2;** yes

**P2;** sometimes fresh fish from the river are half way cooked and the warms which are in it are not dead, when you eat it you can also get infected.

**F2;** so you are thinking that the fish has to boil very well.

**F2;** ok, food hygiene.

**F2;** yes

**F2;** so dirty water.

**P1;** even in the river itself, preventing them from playing in it will help prevent the infection.

**F2**; hee, eeh; are you going to continue washing clothes in the river, the utensils etc. Not there

**P1;** boiling of water for washing clothes is expensive

**F1, F2, P1, P2, P3** ;( laughs…)

**F2**; ok

**F2;** what changes in your community or health systems or local government would help control or eradicate schistosomiasis from your community?

**P3;** strict laws should be put in place.

**F2;** strict laws should be put in place, like what?

**P3;**ok,like these young boys who go fishing , playing in water or along the water bodies end up defecating in the water or near water bodies and when it rains the feces are carried into the water.so strong regulations should be put in place to prevent children from playing in the river.

**F2;** so, regulate fishing to some age group like children of certain age group should not go fishing and this can help prevent the disease. Why particularly children?

**F1;** strict laws

**F2;** strict laws or bylaws also can work.

**F1;** the fishing communities

**F2;** hmm,

**F1;** prevent them from accessing water, bathing and playing in water

**F2;** even fishing, you will find that these children are sometimes sent by their parents to go and fish.

**F2;** so what other things can your community or health systems or local government would help control or eradicate bilharzia,(schistosomiasis) from your community?

**P3;** the government should supply drugs for treatment of the disease and also treatment of water like distributing water guard to people.

**F2**; ok, what else.

**P4;** monitoring of landing sites and clearing of bushes around the access point to the river to prevent people from having open defecation.

**F2;** monitoring of these landing sites, no open defecation along the sites.

**Mod;** and clearing of those bushes so that there is no hiding places for people to defecate.

**F2;**actually along these landing sites ,there’s always a spot left with tall water reeds to help in hiding to do such things ..

**F2, F1, Mod, &other participants ;**( join in laughing….)

**F2;** yes, any other,

**P5;** water for washing hands at every pit latrines

**F2;** how is having water at every pit latrines helpful in preventing bilharzia disease?

**P5;** like after using toilet, you come and wash your hand with soap to remove and kill the germs in order to prevent the disease.

**F2;** are we seeing that chart over there? .number 3.

**All participants;** yes

**F2;** that shows how we can get bilharzia. So in our examples when you get into water that’s when you can have high percentage of getting bilharzia or when you go and fetch the water from the river to your home as you have been saying. Using toilet is to prevent the feaces from being washed by rain into the river or water bodies.

**F2;** so, what other things can your community or health systems or local government would help control or eradicate bilharzia (schistosomiasis) from your community?

You said using medicines for treating water; the government should supply people with water guards to treat water.

**P3;** the government should sensitized the community about the merits and demerit of bilharzia.

**F2;** ok,

**F2;** so what are some of the merits of bilharzia?

**F1, F2, Mod, P1, P2, &P3 ;**( laughing…)

**P3;** for study purposes.

**F1, F2, Mod, P1, P2, &P3 ;**( laughing…)

**F2;** then the demerits

**P3;** demerit is it causes disease.

**F2;** ok,

**F2;** what about the health systems? Anything about the health system that can be done?

**F2;** okay.

**F2; has your family ever discussed use of praziquantel (baya) or any ways to prevent schistosomiasis? If they have what are their opinions?**

**P1;** yes.

**Mod;** praziquantel is the drug used to treat bilharzia (schistosomiasis).

**F2**; that’s the drug used…

**F1, F2;** it’s called “baya” the nickname**.**

**F2;** so praziquantel is a drug in case you don’t know.

**P1;** it’s a medical term.

**F2;** yes, have you ever taken a medicine for bilharzia?

**P3;** yes, at home.

**F2;** what were they calling it?

**P5;”**Ambila”**.**

**F2;** Aaah, “Ambila” is another name, there is “baya”

**Mod;** during the time of ours, it was called “bitroocide”**.**

**F2;** have you ever heard about bitroocide?

**P2;** yes

**F2;** have you ever discuss any issues about bitroocide, baya, Ambila praziquantel in our families or anything to do with prevention of bilharzia?

**P3;** we have ever, but how to explain it now..

**F2;** what did you discuss?

**P3;** they were talking about bilharzia, how it spread and how to prevent it

**F2;** was it at home or at community level?

**P3;** at community level, were gathered together and someone was discussing about the spread of the disease

**F2;** hmm.

**F2;** have you ever discussed it at home?

P1; yes

**F2;** what were people’s opinions about it or what was your take?

**P3;** actually I took it serious.

**F1;** what was your peer reactions about the taking of the drugs, this drug is good or not good. What were you discussing among yourselves there after the discussion?

**F2;** or after discussing with your parents at home about the drug

**P3;** yeah, they were saying the drug so strong, it brings severe abdominal pain, diarrhea and so on.

**F2;** so you were instilling in fear of the medicine

**P1;** fear of the side effects

**F2;** your fellow girls or ladies what do they say about it.or imagine like right now we don’t have the drug here but when they talk about the medicine what come into your mind, or your opinion about the drug?

**Mod;** is question too hard? Or not getting it right? Or you have never discussed about it or you don’t stay home? (Laughing….)

**Mod;** ok,

**F1;** I think we can move to the next.

**F2; who is the most important in deciding if a family member comes in contact with schistosoma mansoni infected waters or receives praziquantel for treatment of schistosomiasis? Why do you think that person is important?**

**P3;** the infected person because he or she is to decide whether to take the medicine or die.

**F2, Mod ;(** laughs...)

**F2;** we are talking about the family, who takes decision?

**Mod;** for instance at home you have your father, mother, yourself and your brothers who takes the decisions on the treatment of bilharzia?

**P1;** is the mother, when the father is not around.

**P3;** for me it’s the infected person.

**F2**; what if the infected person is young?

**P3;** if he is young, then it’s the mother because the father they don’t care about the children. But the mother will be there with the child to decide what to do.

**F2;** one said it’s the dad, the other said it’s the mum, because she is the one who cares about the health of their children. Men don’t care.

**P3**; she is the one who suffers,

**F2;** she is the one who suffers with children. Then others said the mum, when the dad is not there.

**Mod;** why do you say so?

**P1;** I said the Dad because he is responsible for everything in the home.

**F2;** yes,

**P3;** I said the mum, because the dad is always not there at home most time. For example if he is a fisherman he will be always at river fishing making sure the get what to eat and when is infected, it’s the mum to take him to the hospital.

**F2;** men are never at home.

**P3;**they are always up and down, minding of feeding the family on and that’s why my point still stand that it’s the women to make decision at home.

**F1, F2, Mod, P3, P1;** (laughing...)

**F2;** so what are others saying?

F2; yes, ok.

**F2; who should be given the praziquantel?**

**P1;** everyone.

**P2;** infected person, the victim.

**F2;** she said everyone, infected person.

**P3, P4, &P5;** all said everyone because others might not be showing signs of the disease and they are having it so it is better to give everybody.

**P5;**just like other time ,they gave everybody whether you have the disease or not ,this help to protect the body in case it comes ,the worms will find the drug already there.

**F1;** everybody,

**F2;** yes, she also said everybody even if there are no signs, the medicines will treat you and you will be fine.

**F2; so, who do think should not be given?**

**P5;** pregnant mothers.

**F2;** why not?

**P5;** I hear they say when they are given the medicine can cause abortion and miscarriages.

**F2;** the pregnant mothers should not be given medicine because it can cause abortion.

**F2;** any other?

**F2;** yes

**P3;** I was also saying the pregnant mothers.

**F2** ;( laughs...)

**P4;** the young children who are still under aged.

**F2;** so the very young children should not be given the medication. And why do they say the children should not be given medications? In your own reasoning.

**P3;** in my own opinion because they are still young.

**F2, P1; (**laughs….)

**P3;** when they are still young, before starting to crawl and that size shows that they cannot swallow the medicines.

**F2;** are they the only one?

**P1;** yes.

**F2;** to say you people are not in these two categories

**P1, P3;** yes

**F2;** so you can swallow?

**F1, F2, Mod, & Other participants ;(** all laughing…)

**F2; Are there any reasons why you or your family members or community should one take praziquantel?**

**P4;** according to me, there is no any reason why I should not take**.**

**P3**; for my case, there is no reason except when my sisters or my mum are pregnant then they will not take the medicine**.**

**P1;** there is no reason to prevent any person not to take or not be given “baya” (praziquantel).

**F2;** there is no reason. You people are behaving as if it’s this marriage things were they ask is there any reason why this lady should not marry this man?

**F1, F2, Mod, & Other participants ;(** all laughing…)

**F2;** the answer is no reason. (Laughing…)

**Mod;** what if you are sick?

**P3;** when am sick, I will still take.

**F2;** for you or your family members or community there are no reasons why one should not take praziquantel or baya.

**F1;** what if your boyfriend, husband tells not to take the medicine? (Laughing...)

**P3;** unless am told not to take with clear reasons.

**F1;** ok.

**F2;** alright.

**F2; Are there any reasons why a person should not take or not be given praziquantel?**

**Mod;** just as you had said that your mum or your sisters would not take the medicine when they are pregnant.so what is the reason as to why they should not take?

**P3**; for me I have heard that when you take tis medicine, it can cause abortion or miscarriages.so basing on that, I would advise them not to take the medicine.

**Mod;** then somebody had talked about young children, why would you not give them?

**P3;** sometimes it brings severe vomiting and diarrhea which may weakened the child leading to admission and intravenous fluids /rehydration because the medicine is too strong. She (laughs…)

**F2;** because these children are fragile and weak.

**F2;** yes, any other reasons?

**P2;**it’s the same reason because whenever this people who normally give drugs in the community comes to a home with very young children ,they normal tell us that the child is still young and weak to handle the strength of the drug.

**F2;** we are not going to put any thoughts in your mouth. He (Laughs…)

**F2;** Aaah, we have found out that the pregnant women and very young children you would not allow them to take because they are young, this is their reason.

**F2; Access to medications like antimalarial drugs and drugs like praziquantel might be a problem. If it is a problem to you or your family, what are the reasons for this problem?**

**F2;** Let me start with you**.**

**P3;** at times you find some families don’t have money and the prices of the medicines is expensive to afford, this becomes a problem to afford the medicine.

**F2;** affordability, family cannot make it.

**F2;** any other reasons why you think this medicine is a problem to get?

**F2;** yes, you are raising your hands

**P4;** no, I did not raise my hand.

**F2,** ok

**P3;** there are some people who ignore sicknesses; they think the disease cannot kill them.

**F2;** ignoring the sickness and the problem of the medicine, how does it rhyme?

**F2;** but the medicine is there,

**P1;** yes, but they don’t think of buying the medicine that the sickness will heal by itself.

Like when a child is having rashes,(she laughs..)

**Mod;** ”nianga gira” (laughs…) self-enjoyment nick name of a new form of body rashes in Pakwach District.

**F2;** she is talking about people’s attitude towards medications generally. They under look disease saying it will get cured by itself. I don’t know how this will come into access.

**Mod;** we are now looking at access to medications like antimalarial drugs and drugs like praziquantel might be a problem. If it is a problem to you or your family, what are the reasons for this problem?

**Mod;** so we are looking at accessibility or availability of the medications, are they really there? If we are to go to this other point, it speaks directly to you. How my perception specifically look at that disease and its access.

**F2;** it’s more of a stumped. You really want the medicine but how to get it, is the problem.

**Mod;** we are seeing that you really want to take the medication but either the medicine is not there or getting it is very hard. Like for malaria, you may go to the health center and fail to get the medicine because it’s not there or if the medicine is there, do you get it easily? In some places, the health centers are far away and getting there may become a problem to some people or may reach there but the medicine is not there.

**F1;** stock out

**Mod;** yes.

**P3;** there is also corruption.

**F2;** what has corruption do?

**P3;** you will find the government has sent drugs, recently I was admitted in the hospital suffering from typhoid and I was to get injection but was told to go and buy the medicine, that the medicine is not there and yet the medicine was there. My friend who is a nurse and a nearby neighbor also there came from nowhere as if sent by God came and got for me the medicines from the box when am seeing with my two naked eyes, and I was injected, so most of the workers are prompted and they want you to buy and when you don’t have the money it becomes a big problem.

**F2;** ok, do they sell them to you?

**P3;** ok, you buy them from the clinic.

And when you don’t have money, it becomes something very big. When you are seeing the thing, they tell you to go and buy or you give me the money I bring it for you.

**Mod;** so it’s the health workers attitude, having segregations or discrimination, unethical behaviors.

**F1;** segregation of patients

**F2;** monetarisation of free government services

**Mod;** so they want to sell to people, it happens.

**F2;** one more thing that persistent was the funds; they don’t have the money to buy the medicines from the clinics and pharmacies.

**Mod;** other thing under funds, it’s the cost of the medicines because sometimes the government takes long time to supply medicines and the patient may be sent to go and buy from these that pharmacies we have, the Med point, yat keyo, Barry’s pharmacy etc. so if you go to those pharmacies you will find the cost of the medicine may cost thirty thousands, fifty thousand shillings like that making it difficult to access the medicine.

**F2;** yes, any other reasons

**F2;** ok, this is our application.

**F2; Do you think being a man or a woman would make a difference in you or your family accessing praziquantel or using praziquantel?**

**F2;** am going to start from here.

**F1;** in your position as a lady or a woman does it change or affect the use or accessing this drugs.

**F1;** there is something that makes you or hinders you from getting this drug, whether good or bad to get the drugs.

**F2**; your consulting; for the access or for the use.

**F2;** yes

**P3;** I beg your pardon

**F1, F2, P3;** (laughs...)

**Mod;** try to look at it this way, is it easy for you to access this drug in your family

**F2;** or getting it is hard.

**Mod;** like in your own family, is it easy for you being a woman to access the medicines and use it? Do you think you can make a difference?

**Mod;** because the answer would be either yes or no and you give a reason

**P3;** ok, this answer is yes. Reason being in case they are giving medicines in the community, and you got the medicine and took it irrespective of whether you have the bilharzia or not without any sign and symptoms, I believe you will get some changes in you because you have completed the given dose, I know there would be changes.

**F2;** now you being a lady, what changes have you got from getting or taking the medicines you being a lady what changes would you bring, not which changes of the body one gets after medications.

**F2;** am going to give you a simple example and very interesting one, you will find that you are in line for food and the person supervising is a man and he comes to pick the lady to serve first from any other person from the line saying ladies first.so if you are to take such example, it means the access to the medicine has changed simply because you are lady, with such example do you think it’s there? I have given a social example.

**F1;** in some cultures they will always give opportunity to boys or men because the ladies will not be there with us, let us not mind them. So they will say don’t give them.

**F2;** another example they will say, maybe ladies first in every things even in education, just like those days ladies were not allowed to study because they will get married, they were dis advantage to education while the boys got advantage to go to school so with medication access to medicine do you think you being a lady has such advantage or you can make a difference just because you are a lady.

**F2;** hope there is no problem, everything is ok.

**Mod ;**let’s look at this example ;imagine you are at home with children and they are giving medicines, you got initiative to go and get the medicine for your family and made sure everybody took the medicine. Just look at that role you played as a woman because you said that we men are never at home.do you think you played that role simply because you a woman?

**P3;** that is my role to take care of the home, since my husband was away and if he was around he would have taken the children and get the medicines. But since he was not there and I heard that they were giving drugs for bilharzia I had to go and get the medicine to prevent my family from getting the disease.

**Mod;** in summary you as woman and a mother in a family play a big role in accessing and use of praziquantel in a family. Do we all agree with that, or you have something different to say.

**Mod;** Do you think being a man or a woman would make a difference in you or your family accessing praziquantel or using praziquantel?

**F2;** being a woman do you think it’s of advantage, back to the question.

**F1;**for us in our culture you know women they have that empathy ,anybody falling sick, you will see women around so that care they always give makes them actually be supportive to the family of the sick, help or remind them to take their drugs at the right time or even at times going to pick for them these drugs because of that empathy so that feeling in you of always supporting others ,men don’t care they just go walk, bathe and d such kind of things can be an advantage to a woman in accessing or in helping the family to use those drugs praziquantel.

**F1;** are we together?

**P1;** yes.

**F2;** does it apply to you?

**P5;** it does.

**F1; so** which one do we take?

**P3;** put woman’s role.

**F2;** we are giving clues so that you can look at yourselves what advantages that you have.

**F2;** what do you think?

**Mod;** Do you think being a man or a woman would make a difference in you or your family accessing praziquantel or using praziquantel?

**P3;** it would not make a difference.

**F2;** why

**P3;** why, you can’t tell me that it will make a difference in me or family because of the following reasons you will find that you have gone to the hospital and got medications for your family and they took them, so nothing will happen ,it will not change you, so eeh**.**

**F2;**so what makes you go and pick medicines for the whole family ,ok what makes it easy for you to go and get medicine for the whole family?

**F1;** why is it that not a man or a boy should have gone to pick this drug but it is you a woman?

**P3;** first of all it’s my role to make sure that my family are healthy. Secondly as a woman i need to make sure my family is healthy, eeh, how to frame this point is hard.

**Mod;** use alur language, if it’s hard to frame it in English.

**P3;** its ok I can use English, it’s not refusing but...

**F2;** bring it forward so that we can help you up with it.

**P3;** first of all it’s my role as a woman to make sure that my family is healthy, now like when they have announced that they are giving out drugs for bilharzia ,she is to organize her family to go and get the drugs.

**F2;** so there the women are more responsive to some of these programs than men.

**P3;** yes.

**P3;**so she will go and get this medicines for the family to take, so when they have taken this medicine I don’t think it can change them they will still remain the same

**F2;** we are looking at the accessibility of the medicines and the useage

**Mod;** so you as well groomed woman who would organize her family to go and get medicines for her family and makes sure they take the medicine to be healthy and well, those are some of the things we are looking at.

**P3;** okay,

**F2;** to say if you are not there, what happened

**P3;** okay, and the men don’t care so if am not there they will miss the drugs and incase its severe they will get the disease because I was not there and no one bothered to get the drugs for them during distribution.

**Mod; so** to say for her as being a woman it’s always a great deal that can make a difference in her accessing the medication and also for her family because she does not want her family to suffer from schistosomiasis so she has to get some medicines for the family as well rather than the man being there and who may not make up a decision to take medication.

**P1;** so the responsibility is for the woman.

**F2**; apart from that any other suggestions.

**Mod;** any questions?

**P3;** okay

**F2;** you said ok or not there.

**P3;** I wanted to know the signs and symptoms of this disease because we normally fetch water from the river and you may be there with the disease unknowingly.

**F2;** read here, aloud for your friends also.

**P3;** she reads, what are the signs and symptoms of bilharzia.

**The guiding questions:**

Qns. What do you see in the picture?

-I can see a boy with swollen stomach.

Qns. Have you seen anyone looking this in your community?

-They are there.

Qns: What could this person be suffering from?

-I guess its bilharzia. Once you have swollen abdomen, you just get the answer by yourself.

Qns: What could be the ways of getting this disease?

-Playing in dirty water etc.

Qns: How can this disease be prevented?

-Avoid defecating in water

**Then the key point:**

Bilharzia is the disease cause by worms that lives in the blood vessels of the intestines or bladder.

The disease spread through getting into contact with contaminated water, and the worm enters through the skin.

**F2;** ok, thank you for reading and I hope you have got it correctly now.

Thank you again for your time and may keep ourselves safe from diseases.
