# Supplementary material for: Gendered lives, gendered Vulnerabilities: An intersectional gender analysis of exposure to and treatment of schistosomiasis in Pakwach district, Uganda
Source: PLoS Negl Trop Dis. 2023 Nov 10;17(11):e0010639. doi: 10.1371/journal.pntd.0010639 (PMC10684070; doi:10.1371/journal.pntd.0010639)
Supplement: S1 Data — (ZIP) [file pntd.0010639.s001.zip › FGD Schisto Interviews/FGD FEMALE 46-65 YEARS PKH.docx]

**GENDER INTERSECTIONALITY**

**AND**

**SCHISTOSOMIASIS IN RURAL UGANDA**

**TRANSCRIPTIONS AND TRANSLATIONS FOR FOCUSED GROUP DISCUSSION**

**PAKWACH TOWN COUNCIL, KALALO COTTAGES INN**

**FIRST GROUP ;( GP1)-FEMALE AGE BRACKET FROM 46-65 YEARS.**

**F1-Facilitator 1**

**F2-Facilitator 2**

**Mod-Moderator.**

**Gp-Group**

**P1-Participant 1**

**P2-Participant 2**

**P3-Participant 3**

**P4-Participant 4**

**P5-Participant 5**

**Introduction of the topic:**

**F1**; you are all most welcome our mothers; we are going to introduced ourselves briefly so that we can get to know each other. I will start by introducing myself first, am called Phillip and am a facilitator and colleagues are Noah Okumu a facilitator, we have Ocama Peter our moderator, Nakiranda Salama is our Administrator, so we can continue with the introduction Oyungrwoth Nelfa from Mubogo central, Janet Obbo from Mubogo south, Olul Janety from Mubogo central, Nyatho Grace from Mubogo south and lastly we have Owoda Alice from Mubogo south thank you all for coming and for the brief introduction.

**F1:**There is a big differences in our families at all times we have this disease and to say out of 10 people at least 5 people can have this disease have you seen ,at least 5 people can have this disease have you seen and in reality, it’s even more than Corona.

So i think within this short time we are going to have, we are going to listen this disease in reality its strength, teachings and how are we going to prevent or stop it Heee, the way they thought it and found it, how shall we prevent it, so the big question will be led by this big man here Eeheeh, so let say them and don’t mind, what you understand, we shall do and we shall mix up, Heem.

**F2:**Aah ,we are going to start, we all know that there is a problem of bilharzia with us, and we have questions and they are how many, yeah they are 12 questions exactly and all of us will answer one by one respectively.

**F2;** **what activities do you or your family or relatives perform that might lead to infection with schistosomiasis?**

**P1;** I would like to thank this program, because as human, work we do at the river banks we go buy ‘onang” (small fish) and remove the scales and clean them from the water and this can make us get bilharzia.

**F2;** they buy the fish, process it, then clean it and pour in the water.

**P2;**As for me I normally see after cooking and have keep it not well and may be this children who have open defecation, the flies can come on this food and this can lead to bilharzia infection, to me and my family.

**F1;** open defecation of children in the compound

**F2;** open defecation of children in the compound

**F1;** open defecation of children in the compound, Heeh like that.

**P2;** yes if I leave children to pass stool any how in the open behind the house.

**F2;** Heem, open defecation.

**F2;** yes,

**P3;**I would like to add on the water ,like the family members ,the men who normally go fishing and remain with very small shorts sometimes they deep dive into the water ,and this is also one way of getting this bilharzia.

**F2;** the fisher, the fishing force, eeh go fishing and a times dive into the water to do other things.

**F2;** yes.

**P4;**and some are like this, due to lack of latrines ,people end up having open defecation in the garden, and when you go farming /digging unknowing with the dews, the bilharzia/ova end up getting attached to the grass ,hence entering your leg and end up having the disease.

**F2;** open defecation and lack protective gear, putting on protective gear

**F2;** Yes Mama

**P5;** I want to add again on this bilharzia ,as we enter this water ,we also sometimes get the river sand to brush our teeth with it and this is also how we can get this bilharzia .

**F2;** Fetching of water and directly using it to wash their faces.

**Mod;** and some people use the, soil aah the river sand to brush their teeth and the same time

**F2;** but still use the same water to rinse,

**Mod:** yes, so you use to rinse and spit it in water again (laughs...).

**F2;** so we think…is there anything else?

**F2;** so, aah secondly we are asking you Why are men more likely to be infected faster than women in some communities?

**F2;** start.

**P4;** for me am thinking this way ,men can easily get the disease than women because their livelihood is in fishing in the water and some time you find others fishing naked and get their body parts open, and they enter.

**F2:** so is like the men, most of them go fishing and most of the time they are like relatively naked and their body is more exposed than the women.

**F2;** eem, yes

**P1;** like the young boys some time they go swimming in the river and this can make them to get the disease.

**F2;** we are still on adult male, male... (Laughs...)

**P1;** they are also male.

**F2;** so swimming activities of the men, they go swimming and obviously when you are swimming you are naked and get exposed.

**F2;** yes;

**P3;**I would like to add about these men who go to relax and end up getting drunk and fall on the swampy areas rolling in the mud and end up getting the disease.

**F2;** so blame it on the drinking of the alcohol? (Laughs...)

**P3;** yes, it’s the drinking of the alcohol.

**F1;** how could we rephrase this,

**F1;** is it the intoxication that make them restless

**F2;** we could rephrase this, more of the alcohol, eem yes more of the intoxication

**Mod;** Alcohol intoxication and end up falling down in the swamp/mud

**F1;** yeah

**Mod:** because of their drinking habit

**F2:** yes, you were having other suggestions?

**P2;**yes,I like to talk to the adult men the way some time they get it by deep diving in water to get “koppa”( big snail, use as fishing bathe)

**F2;** what do they get?

**P2;**”kopa’’

**F2;**’kopa’’eeh, eem

**P3, p4;**the snails.

**P2;** and so this snails could be harboring this bilharzia and cutting it to use,this worms can enter the body through this way.

**F2;** deep divers’ eeh, this deep diver’s eem

**P2;** so they can enter through this way.

**F1;** But also this act of catching these snails there….

**F2;** so that deep diving.

**Mod;** so deep divers go looking for this snails being used as a fish bathe

**F2;** called ‘’koppa’, eeh, so that koppa is used for fishing?

**P1, P2, P3;** yes

**P2;** so they cut it open to remove the soft part for fishing.

**Mod;** you see the snails itself there is a soft part/tissue inside is removed and placed on a hook for fishing and the fish would come to eat them and get caught.

**F2;** eem, you had any other suggestion?

**P5 ;(** laughs…) the suggestion you have mentioned it up.

**F2; aah on the other side, why are women or their children more likely to be infected in some communities?**

**F2;** Let me start with you today.

**P2;** I have found out that women can also get bilharzia, because they go fetching water in the river, they enter into the water unknowingly, the worm can enter in your body through your legs.

**F2;** fetching water, women are involved in fetching water

**F1;** and they are always good doing it.

**F2;** yes,

**P5;** yeah, we women can get it in many ways, more than men because of the work we do using our hands are many like going to the farm to weed and may find somebody had opened defecation and stool could be having bilharzia, you step on it, or handle it unknowingly and get it and most men get it from the river. We women have a lot of work that we do with our hands.

**F2;** Aah can we make some clarity here a bit, because we need some help .you know were giving teaching at first about this disease, this disease at most time we get it from water/ river so the work we do from the river, to say as long as the stool /fecal is still in the community, and you get it, you don’t get the disease until it gets in water snails and work or enter the water then you are able to get the disease.

The activity like open defecation in the community when washed away by rain and enters into the water bodies/ swamps that’s when the water gets contaminated and when you get into it you can get the disease.

**F2;** so for the women,

**P4;** am thinking the women, the bilharzia can easily get them easily as they take bath in the river as they go to fetch water and decide to reduce the heat from their body and bilharzia can get them.

**F2;** taking bath,

**F1;** eeh bathing

**F2;** as they go to fetch water, they take a bath there.

**F2;** yes Mummy,

**P3;** for women getting bilharzia is easy for us, because we go to the same river to fetch the water, we pull our clothes up and enter in the middle, also decide to wash our face with it and some of water end up being swallowed.

**F1;** entering in water.

**F2;** entering into the water, you have to fetch and at times washing their faces, you know.

**Mod;** so we can write as, the direct taking of the water right from the source of course now, the entering we look at the fetching the water and another activity is the act is the setting of the water for washing face and drinking it direct and wash your face with it.

**F2;** Eem, eem

**F2;** Mummy,

**P4;** am adding on that a bit, as she talked about pull up clothes, for example like in in Mubogo landing site, you may find out that there is a lot of water weeds and you may be required to move further in the middle and end up pulling your clothes above the waist and leaving way open for the bilharzia to enter freely.

**F1, F2, P1&P2...** (Laughers…)

**F1;** so they like sweeping to get clean water in the middle.

**F2;** any other? Or let me ask, are there some women who wash ditches/utensils at the river side?

**P5;** they are there.

**F2;** what of clothes?

**P5;** they also do wash.

**F2;** try to look at the activities women normally do at the landing sites ,just look at Mubogo, and the landing sites in town, try to imagine the activities that take place along the river side.

**P4;** I have seen it this way, like during the dry season, the ground is always hard ,people tend to plant greens vegetables near the water bodies to get some wet ground for the green vegetables and also fetch water from the river to pour on theses vegetables ,and through this one can also get bilharzia.

**F1;** Pumpkins...

**F2;** on vegetables especially, eem.

**P2:** I was saying like for the women who do business of fish are also there at the water bank, they buy and wash and clean the fish in the river and they will take some time to clean the fish and this can make the worms in their body.

**F1;** Washing the fish in water

**F2;** you mentioned that somewhere, you washed the fish and processed the fish in the water.

**Mod; the** fish mongers that they clean their fish in water, remove the scale and the intestines in water and this can make them get the disease.

**P4;** and another things is that carry clothes to wash at the river and I collect water to wash clothes and carry them to rinse them in the water.

**F2;** ok, washing of domestics.

**F2;** Ok, are others there?

**F2;** what changes in lifestyle can you or your family make to prevent you from getting schistosomiasis

**P5;** am suggesting that one way to prevent getting bilharzia/ schistosomiasis whenever we have collected water from the river we must first boil it , allowed to cool and filtered before drinking.

**F1;** ok.

**F2;** she said that when you fetch water you need to come and process it at home boiled it, let it cool and then you use.

**F1;** yeah

**F2;** ok, Secondly.

**P4;** another one to prevent entry of bilharzia in my family is by stopping this bathing in the river. We should take bath in the bathing shelter. Secondly, we must dig pit latrine at home and use it.

**F1;** ok.

**F2;** two things, ensuring we avoid bathing directly in the river but bathe at home, using bathing shelter.

**F2;** and secondly, we have to use the pit latrine. Waste management, we should use the pit latrine.

**F2;** what else?

**P1;**as for me ,to prevent bilharzia from my family, I should make sure that the utensils for drinking water must be clean and the food am preparing at home should be well covered to avoid flies carrying fecal matter from the neighborhood families without pit latrine.

**P1;** and we must eat hot food, not cold food which has been contaminated by flies, through this we can prevent bilharzia from our families.

**F2;** what else?

**P5;** after fetching water from the river, we used it to wash our utensils, thus we must allow the utensils to dry properly before putting food on them because the water may have bilharzia and you may eat the food which is contaminated and get infected.

**F1;** ok

**F2;** ok, it’s still related to food hygiene.

**F2;** yes, any other?

**P2;** for me am suggesting that whenever we have fetch water from the river, we must boil and filter it before putting it in the pot.

**F2;** filtering of the water from the river. eem.

**F2;** yes,

**P3:** nothing much but am talking about the general cleanliness of the home, the cleanliness must be there at home to prevent disease.

**F2;** improving on sanitation at home

**F2;** when you talk about cleanliness, what do we look at..?

**P3;** am talking about pit latrine must be there, rubbish pit, drying rake, etc.

**F2;** general improvement of sanitation.ok

**F2;** Aaah,

**F2; what changes in your community or health systems or local government would help control or eradicate schistosomiasis from your community?**

**P4;** am suggesting that, the government must supply us with drug “Baya” (praziquantel) to my nearest health unit.

**P4;**Testing of stool, so that in case they found out that am having the disease, I can be given the medication since its available in the health centers.

**F2;** government should improve on the diagnosis and treatment, the government has to improve on the area of diagnosis and treatment.

**F2;** yes.

**P5;** it’s also the same, the government should remember us and give us medication to take, not waiting until we are infected then we are given medication. Just like what they say that prevention is better than cure, so that once we get the disease we are protected already.

**F2;** she is thinking of more of prophylaxis, yeah prevention she said is better than cure.

**F2;** what other suggestion?

**F1;** your community there.

**F2;** your people in your area, what must they do? Just like we are talking about Mubogo east,

**F2;** what is the name of your village?

**P3;** Mubogo South

**F2;** yes.

**P1;**Mubogo central.

**F1;**those people in the central..

**F2;** the people in the central what can they do?

**P1;** in my own opinion the people in the central, should use the knowledge we have acquired to make sure that good sanitation is there, like pit latrine must be there, bathing shelter, drying rake, rubbish pit and drying line must be there so that you have a clean home and you can prevent any diseases whether bilharzia or any other disease because prevention is better than cure.

**F2;** is that like the whole community?

**P1;** yes.

**F2;** the community should ensure there is sanitation

**F2;** alright, (laughs...)

**Mod;** other things we should not forget is washing our hands after visiting pit latrines, before and after eating or whenever the hands are dirty.

**F2;** so,we have suggested that the government should look into diagnosis/testing and drugs. And the community must make sure home cleanliness/good sanitation must be there.

**F2;** Aah,

**F2; has your family ever discussed use of praziquantel or any ways to prevent schistosomiasis? If they have what are their opinions?**

**P4;** like for me, we have discussed like the government programs of giving out medications for bilharzia, I have told them to take the medicine to prevent bilharzia.

**F2;**now like before the program of giving drugs, have you ever discussed as a family ,putting into consideration that drug and testing kits are not readily available ,so for us as family we are going to do the following?

**P4;** yes. we have discussed and agreed to avoid bathing in the river, and also boiling of water for drinking. And this tap water still comes from the river and the chemicals that they put in it may fail to treat these worms.

Thirdly, the too much washing of clothes in the river, we should fetch water and wash clothes at home in order to prevent the spread of bilharzia.

**F2;** yes.

**F1;** yes they have ever discussed.

**F2 ;(** laughs...) but have you given some lesson on the ways to prevent the disease but you have not sat as a family to agree on what to do.

Or what have you agreed to do?

**F2 ;(** laughs...)

**P5;** May I say out something also?

**F2:** yes can talk.

**P5;**am talking about food, we should eat food which is well prepared and preserved very well and sometimes you may find out that children tend to pick eat any dirty /contaminated food they find on the ground and this may cause disease.

**F2;** so she has talked of food hygiene at house hold level and avoiding water, washing clothes at the river.

**F2;** so, to question no.6, have you picked anything, Ocama?

**Mod;** Ocama reads the questions,” **has your family ever discussed use of praziquantel or any ways to prevent schistosomiasis? If they have what are their opinions?”**

**Mod;** so the first answer is yes, they have discussed. This is related more to the preventive and people’s opinion on the agreed ways of preventive measures like avoiding bathing and washing clothes in the river and their individual reactions whether positive or negative.

To say have they accepted the suggestion and are they following the measures or not?

Like boiling water, are they boiling water,

**F2;** bathing in the river

**F1;** is there some changes?

**P2;** they accepted and have been following it but sometimes we fail to boil the water.

**F2;** so they have discussed.

**Mod;** so you can say that they have discussed and agreed though there could be negative and positive reactions.

**P2;**we have been using solar water dis infection ,using some bottles until when it was broken that’s when we got some problem with boiling water which is hard.

**F2;**eem ,solar water disinfection.

**P2;** yes, though sometimes we get busy and get water direct from the tap and pour it to the pot for drinking.

**F2;** any other person with different suggestion, or what you have been doing?

**P5;**boiling water, we have been boiling water that’s why for me I don’t take water any how when am not at my home ,because some people don’t boil water.

**P5;** and sometimes we use water from the tap and use treatment like water guide, acqua sipi,

Mod; what is the name of the treatment tablets, you have been using?

**P2;** acqua sipi

**F2;** acqua sipi, or acqua safe?

P5; sorry I was forgetting the name,

**P5;** It’s acqua safe.

**F2**, F1 ;( laughs...)

**F2;** do we still have water guide at home?

**P5**; yes.

**Mod ;**of course we can now say that as one of the preventive measures like boiling water, use of water guide, water safe and then solar, solar..

**F2;** solar water ,disinfection.

**Mod;** yeah solar water, disinfection

**F2;** solar water disinfection

**F2;** aah,

**F2;** so we want to know about this,

**F2; who is the most important is deciding if a family member comes in contact with schistosoma mansoni infected waters or receives praziquantel for treatment of schistosomiasis? Why do you think that person is important?**

**P5;** from my place it’s the parents are the one to talk about the taking of the medicine.

**F2;** what of the adults?

**P5;** even if adults you have to help them, tell them to take the medicine for bilharzia and may not be having the knowledge so that they can get improvement just as my colleague had said they should bring for us medicine nearer so that we can start taking the medicine early, like for us Jonam people here we are always exposed, and can easily get it.

**F2;** eem, so the most important people to talk about bilharzia are the parents

**F2;** and why do you think it’s the parents to talk?

**P5;**it’s the parents because they have more knowledge than the children and even some people you would see they have less knowledge, then you have to advise them.

**F2;** so, because the parents are more knowledgeable. Eeheeh.

**F2;** so between you two parents, who normally talks?

**P5**; like for me am alone, and it’s me talk.

**F2;** and those who are two, two together.

**P2, P3, P4, and P5 ; (** laughers.) we all single parents (widows) except one person.

**F2**; eeh. who talks? the family head.

**F2**;I need an example where there is a man and a woman staying together, and who is the last to talk?

**P1&P2;** the husband

**P1;** because when a child is sick,he is the one to take care and for me I will help.

**F2;** what if he is the one?

**P1;**I will help him.

**F2;**if it’s now you?

**P1;** he has to help me.

**F2;(**laughs..)

**F2;**so it’s the head of the house hold because ,one they are the one with the knowledge, and two because they are the bread winners ,support the family more so financially.

**F2;** So, who should be given the “baya”or praziquantel?

**P3;**A person with bilharzia disease.

**F2;**and the bilharzia disease can get from children,youths,mothers,fathers,fishermen,and farmers.

**F2;**so,the first are the infected people. Secondly

**P2;**for me am suggesting that everyone in Jonam.

**F2;**So who are the people of Jonam?

**P2;**the children and the adults all.

**F2;**So everyone.

**P5;** Yes. Because we are at risk. So that we can have the drug ready in our bodies.

**F2;**everyone without any segregation.

**P1,P2,P3;** yes everyone.

**F2;**So you are suggesting everybody, or maybe they can start with some group of people.

**F1**; ok.

**P2;** my suggestion is that they should first start with the fishermen.

**F2;** so, you are suggesting to start with the fishermen?

**P2;** yes, because they normally go fishing every day and most especially the one who normally pull the nets ‘korokota” and followed by the deep divers who go looking for big water snails.

**F2;**ok,are you together?.

**F1;**yes.

**F1;**the first one was everybody

**F2;** they said those who are infected, they said everybody, they also said on the other side the priority would be the fishermen.

**F1;**ok

**F2;**so you are thinking of the fishermen, what of the children, youths, married people, mothers, fathers and uncles?

**P3;** those are all in the group of everybody.

**F2;(**laughs..),may be theirs is separate…

**P5;**you see, even if you start with these fishermen, they will still go back.so they should start with the children.

**P4;**fishermen normally ease themselves in the river and that’s why the spread is high even.

**F2;**So, are there any reasons why you or your family members or community should not take” baya” praziquantel?

**P2;**these young children(Babies)

**F2;**Babies from what age?

**P2;** Children one to four years.

**F2;**Why..

**F2;** She said Children/babies from zero to four years.

**F2;** and why should you not give them?

**P2;** because they are still young and the drug is very strong and can harm them.

**F2;** but these children, do they really get this bilharzia?

**P2;** they can get, as they play in this collected water around homes.

**P2 ;**But not so much, because a lot of bilharzia are often got from the river.

**F2;**and this river, are there some mothers who go with their children there?

**P2;(**laughs..)

**P2;**but these days they are not there, especially these fish mongers/those who buy fish for selling and also some mothers who would carry their babies at their back to go fetch water.

**F2;**two weeks back, one of the in charges called me and said they have got a 2 years child…(.laughs)

**P2;**the child was going to the river?

**F2;**No.the child had bilharzia.

**P5;**yes,some mothers go with their children to fetch water and you find them bathing their children in the river.

**P4;**through that these children can get the disease.

**F2;**yes! That’s what we want to hear.

**F1;(**laughing….)

**F2;**because if we found a child of 2 years with bilharzia, how did this child get the disease?.it a parent who took her they get water in the basin and bath them when its hot.

**F2;**so when you say the children should not take the medicine, you would have killed them.

**F2;**yes,so

**F2;**is there any other person that should not take medicine?

**P4;**no one.

**F2;**not there?

**F2;**yes

**P2;**am thinking of the pregnant women.

**F2;**the pregnant women.

**F1;**yes,

**F2;**any other

**P4;** those who are very sick and admitted in the hospital.

**F2;**yeah,those who are ill, very sick.

**F2;**any other suggestion

**F2;**not there? everyone can get now. What of a person who has vomited blood?

**F2;**ok, lets go to the ninth Question

**P4;**first help us explain about those who have vomited blood..

**F2;**no, I was asking from you people.

**P3,P4;**We were looking at the sick ones in general.

**F2;**ok.

**P2:**May be when he has just vomited the blood, he cannot take. But after getting some strength.

**F2;So, are there any reasons why you or your family members or community should not take” baya” praziquantel?**

**P5;**for making them take the medicine..

**F2;**yes,is it there?

**F2;** yes you talk.

**P2;**Am suggesting that ,any person who has tested positive for bilharzia, then he can be given the medicine.

**F2;**so you are suggesting that they should first test them, and why should they be tested first?

**P2;** because it’s better to be tested first so that they can get the right medicine.

**F1;** number nine;

**F2;** number nine, should not take….Eeheeh

**F1;** yes

**F2; so, are there any reasons why you or your family members or community should not take” baya” praziquantel?**

**P5;** there is no reason for not taking medicine, but there are some people who are stubborn not to take the medicine.

**F2;** Ok, let’s leave stubbornness…but let think of a reason that would make your family or some community not to take the medicine, or there’s any reason.

**P2;** some people say when they take this medicine for bilharzia; it makes them become sick yet they were normal and ok. The sickness like vomiting, diarrhea makes them not to take the medicine.

**F2;** so, those myths and the belief, of the side effects like the nausea ,vomiting as a result of the medicine bringing another disease so that is the reason for them not to take.

**P5;** that’s why some people when they are given medicine to go and take at home, they don’t take and throw them away.

**F2;** so, is there any other reason

**F1;**its like when you take the medicine, it brings another disease ,is it like that?

**P3,P5;**Yes.

**F1;**that’s the belief.

**F2;** yes, and that’s why I said a myths or belief .

**F2;**is there any other reasons?, or its only that?

**P5;** yes. It’s only that.

**F2;** ok.

**F2; access to medications like antimalarial drugs and drugs like praziquantel might be a problem. If it is a problem to you or your family, what are the reasons for this problem?**

P5; when there is illness in a person that cannot allow the person to take.

**F2;**  just try to imagine, a healthy person like me.

**P2;**Am thinking of the strength of the medicine that that has been reported that it brings some weakness in a person, and if you are to take this medicine ,you will not be able to work in your garden for almost one week and yet time is going to plant or harvest your simsim from the garden.

**F2;** Sick ones, those who are ill and then work.

**F2;**Sometimes there is this kind of medications like antimalarial drugs and drugs like praziquantel might be a problem. If it is a problem to you or your family, what are the reasons for this problem?

**P1;**the problem of getting these medications is that they are never there or available in the government health centers at most time and they are not enough for the people. If they were enough, it would not be a problem.

**P1;**And since its never available in the government facilities, then you have to buy expensively and that’s another big problem because we don’t have the money.

**P1;**so we are requesting the government if they can bring enough drug in the facilities, the sick ones can go and get it freely, and this would have helped /saved them a lot.

**F2;**one thing is the shortage or no drug in the health facilities. Secondly, their economic status, they may not have the money to buy these medicines from other units, the drug shops.

**F1;** True.

**F2;**So,one is the access of the drug, availability and affordability of the drug.

**F2;**so ,is there any other problem that we are seeing?

**F2;**yes Mummy, you have something to it

**P5;**There is no other problems, other than problem of the drugs and the money to buy them because from the hospitals they are not there and you don’t have the money to them. As it has been said, once sicknesses get a poor person, he dies.

**F2;**so all of us are thinking the same way?

**P1, P2, &P3;** its only drug problem.

F2;ok,our last question for this session.

**F2; Do you think being a man or a woman would make a difference in you or your family accessing praziquantel or using praziquantel?**

**P2;**for me am suggesting that it needs a man, as you see sometimes sickness may be in the family, it will need a man to look for ways of getting the drugs and money to support.

**F2;** ok,let’s talk about you being a woman does it give you any changes, benefit, or difficulty in accessing or using this medicine?

**F2;**yes let’s talk as women.

**P5;**so as a woman, when I get this medicine and have taken it ,I will get better and be able to support my family as I will be having some strength.

**F2;** so, that’s when we are talking about the benefits. What of its access to these medicines?

**P5;** of what, bringing it in the house?

**F2;** yes.

**F2;**we have talked about so many things in respect to this drug, so we are looking at you as a woman does it add anything like changes, benefits or difficulty in accessing or using this medicines.

**P1;** for me, as a woman the benefits I have seen from God giving me, makes me to do the same benefit to others.

**Mod;** so let’s have this example, taking that am a man and you are a woman, do you think you can have more access to his drugs than your husband?

**P1;** he has asked, as about women…

**F2;** yes, so as you do think you are the one to deal with the drugs giving or it’s the husband/

**P1;**as a woman with children, you are supposed to do whatever it takes to make sure your family is ok. And sometimes these men are never home, then you will start from 1to 10 doing everything.

One of the most important thing is to have drugs in the house for these people you staying with and whenever there is sickness you will be the one to carry this child to the hospital, their father will come back later asking where the wife has gone from the children.

And the children will be the one to tell him that she has taken the baby to the hospital.

So it’s the responsibility of the mother to check for the wellbeing of the children/family, check on them every morning to find out whether they are fine, they have eaten or child is not eating.

So that is the changes you have to put in your family and must know as a woman.

**F2;**like they are always available and they know all the affair of the home than the men. And they are easily accessible at home most of the time.

**F1;**in terms of the medical access..

**F2;**they are easily, the seeking behavior. They are the one to know how the day is and where do i get the services.

**Mod;** and two ,even if they have the medicines, they are the one to administer and even have the higher access of medicines right from the health facility and even at home.

**F2:**am seeing some new suggestion.

**P5;** am having suggestion about time, we are the one who have time of giving drugs to these children and if you are to give it to the father, he might be even at work.so for us even if we are too busy, you will be able to give the medicine at the right time ,seeing its now eight pm.

**F2;** alright,any other?

**F2;** I think most of the questions, we exhausted them, any other suggestion we have not added here.

**F2;** thank you so much for your participation, and have a great day.
