# Supplementary material for: Gendered lives, gendered Vulnerabilities: An intersectional gender analysis of exposure to and treatment of schistosomiasis in Pakwach district, Uganda
Source: PLoS Negl Trop Dis. 2023 Nov 10;17(11):e0010639. doi: 10.1371/journal.pntd.0010639 (PMC10684070; doi:10.1371/journal.pntd.0010639)
Supplement: S1 Data — (ZIP) [file pntd.0010639.s001.zip › FGD Schisto Interviews/FGD MALE 18-45 YEARS Pakwach.docx]

**GENDER INTERSECTIONALITY**

**AND**

**SCHISTOSOMIASIS IN RURAL UGANDA**

**TRANSCRIPTIONS AND TRANSLATIONS FOR FOCUSED GROUP DISCUSSION.**

# Abbreviations and acronyms

FGD – Focus Group Discussion

GP4 –Group Four.

F1-Facilitator 1

F2-Facilitator 2

Mod-Moderator

P1-Participant 1

P2-Participant 2

P3-Participant 3

P4-Participant 4

P5-Participant 5

**GP4. MALE (18-45) FGD**

**Introduction:**

**F2;**you are most welcome my name is Noah Okumu and am a facilitator for discussion today and my colleagues are phillip,Ocama Peter our moderator,Nakiranda Salama is our Administrator. Then from left hand we have Tolith Emmanuel, Ocircan Santos, God’s power Ismael, Uhuru Stephen and lastly Ofoymungu Pius, you are most welcome once again.

Let us organize our masks; you will be required to remove it when talking so that our voices can be heard. We are group number 4 age group 18 to 45yrs I belief we are all in that age bracket. We have a total of 12 questions and we answer them one after the other taking 5 minutes for each question so that we take only 1 hour. We shall do like that and those consent forms hope you have filled them.

**All the participants;** yes.

**F2;** let us be free and you can give us your experiences, whatever we are going to discussed will be confidential and no calling of names. We are together.

**All participants;** yes.

**F2;** our first question is, (he reads the questions…..)

**F2; what activities do you or your family or relatives perform that might lead to infection with schistosomiasis?**

**F2;** let start with you.

**P1.**the activity that can make us get disease is fishing

**F2**; yes, try to increase your voice; fishing

**P2;** moving in contaminated water with bilharzia

**F2;** moving in contaminated water without any protective gears.

**F2;** you can tell us.

**P3;** some places near water bodies you find people defecating in there, when it rains the fecal matters are carried to the water bodies.

**F2;** open defecation around water bodies.

**P4; we have** drinking unboiled water most time tend to spread it.

**F2;** drinking unboiled water

**P5;** snails mining

**F2;** snail mining

**F2;** there is fishing, fetching water

**F2;** ok, another one.

**F2; why are men more likely to be infected than women in some communities?**

**P4;** first of all, we have gender balance. Men tend to look for various ways of survival more than women then one way is looking for life from water

**F2;** like what?

**P4;** like digging of earth worms from mud used as fish bates.

**F2;** what is that? Mode of fishing

**P4;** yes

**P5;** those are earth worms used for fishing as a bate.

**F2;** so they first dig to get the earth worms from the mud.

**P4;** and we have to enter in very dirty water and that’s where these worms tend to hide in there.

**F1;** that is...

**F2;** that’s still an activity related to fishing where they have to dig and get the earth worms to be used as a bate on a hook, how do they call it? Digging earth worms.

P4; digging of earth worms

F1; is it fishing

F2; it is not fishing but the act of looking for a bate for fishing.

P5;they dig mud looking for these earth worms, which are later put on hooks as bate to get the fish.

F1; what is that?

F2; digging of earth worms, he (laughs…)

F2; and he put it in the areas of gender roles that men are to defend over the family.

F2; ok, you had some suggestions? Has it got lost?

P4; like we men the activities that we do …

F2; we are talking about men generally

P3; ok, let me say something. Like we men sometimes we go and dig near the water bodies where these water snails are to plant greens.

**F2;** liking farming along the water bodies to plant greens.

**P5;** to add on what they have said in reference to activities men risk their to do along river, like this water which women normally fetch, at times it’s hard for them to enter, so the men has to enter and cut down the water reeds and papyrus to create passage for the ladies because the women tend to fear, that the work is hard.

**F2;** so one is farming along the river or watered areas for vegetables then the male also take that risky work of, like where women fetch water from at times it gets blocked; it’s the men who go and open. They have to dig to make sure the women have access to water because the women cannot do it.

**F2;** as we keep on thinking, yes

**P5;** another one is swimming in the river.

**F2;** swimming, men love swimming more than women. Women don’t love swimming.

**F2;** so why do you think men are more likely to be infected than women in some communities

P2;in some places like koppio river, men normally go to very dirty points to place their hooks and can stay in water for some times you may find that where you are standing in has a lot of water snails making it easy for the worms to get into your body.

P5; what he is saying is that there is this type of fishing, where you have to use the hooks but you have to go down the water to get this big water snails to be used as bate.

**F2;** so they first go down into water to get these things the bate then prepare it...

**F1;** they keep diving with the purpose of...

**F2;** is to get, is also a bate?

P5; it’s also form of bate, they are like these snails with big cells “koppa”

**F2;** they call it “koppa”

**F1;** they deep dive to get the “koppa” thing (big size of snails used as bate for fishing)

**F2;** have you ever deep dive to grab them?

P4; it’s not easy to do that, and when you have little air?

P2; whenever they are fishing, they just drink this water direct.

**F2**; for the male when they are fishing, they just drink it direct, there is nothing like…

**F2;** ok,

**P5**; there is a channel where this water flows contains all short of wrong things that flows into the river.

**F2;** so these are still connected to the fishing, fishing modes and types of fishing.

P5; when you are removing these, you have to get them.

F2; you have to get them, ok.

F2; on the other hand,

**F2; why are women or their children more likely to be infected in some communities?**

P2; fetching of water for domestic use, like washing of plates, cooking

F2; they fetch the water and when they reach home they start using it direct.

F2; so fetching of water. Yes

P3;sometimes these children go playing in dirty water and may end drinking that same dirty water they are playing.

F2; what.

P3; children play in dirty water and also drink the same water.

F2; they play in water and drink it.

F2; yes

P1;Eeh,for examples ,women normally fetch water from streams where you may find earth worms, hook worms and others may be present in the water after most especially after heavy rains and use it to bathe their children.

**F2;** fetching this water and using it bathe children at home.

F2; yes

P4; we have walking bare footed after raining.

F2; no protective wears like shoes, they walk bare foot

F2; so they don’t put on Gum boots

**P5;** children are like this; where they love playing is the same place they defecate in and end up getting the disease there.

**P4;** they urinate also there.

**F2**; ok, so children where they play, they defecate there.

**F2**; open defecation, ok.

**F2;** alright, what else do you think women or their children more likely to be infected in some communities?

**P5;** if you are to follow the ways women go the river is more than men. A woman can go to the river to fetch water for about five (5) times and a man may go fishing once a week, this fetching of water alone put this woman at a high risk of getting infected than this man who goes fishing once a week.

**F2;** so their frequency, like in a day they can move five (5) to six (6) times to fetch water but for the man sometimes once a week and uses the money for one week but for the woman every day.

**F2;** Aaah,why do they fetch this water? Or why do they like accessing water?

P2; for drinking,

F2; for domestic use

P4; yeah for domestic use.

P5; in our culture it’s the women to do that

F2; that’s why cultural

P5; it’s their role…

P4; that’s why they are still following that cultural belief of women fetching for the home and its very important. When pipe water was brought ,there was life style changes but still women finds its easy to get water from the river, that’s why they can move five (5)-(6) times fetching this water just like what master had said.

**F2; what changes in lifestyle can you or your family make to prevent you from getting schistosomiasis**

P1; boiling of water

F2; drinking boiled water

P2; using of tap water

F2; using safe water

P3; avoid walking bare footed

F2; use of protective wears, like gumboots.

F2; what other life style?

P4; we have use of medicines to treat the disease (bilharzia).

F2; we are still on life style changes

F1; what changes in your life you can make to prevent getting bilharzia disease

P4; ok.

P4; digging of pit latrines

P5; avoiding open defecation,

F2; digging and using it, he (laughs…) some people they dig the pit latrine and don’t use it ,they defecate outside.

F2; yes

P3;I want to add something, after preparing the pit latrine they must put tippy tap next to it so that after using the latrine ,you have to wash your hands.

F2; hand washing

P2; avoid defecating and urinating in the water / rivers.

**F2;** avoid direct urinating in the river. There is no urination without defecation.

**F2, F1, P1, P2, P5; P3** ;( laughing…)

**F2**; what other changes are…..

**P5;** am seeing what women do, people should do real farming instead of collecting water weeds for sources.

**F1;** avoid farming?

**P5;** that’s not real farming because people just go and collect some plants from the river just like any other wild plants. They do that because of lack of food. If they are to practice farming they will not be going there to collect the water plants.

**F2;** food security

**F2**; yes,

**P3;** avoid half cooked meals, for example like when people do open defecation, these fish can feed on these fecal matters and when you have cook this fish you can get disease like hook worms and others.

**P5;** that practice happens down here, where you find some fishermen start eating this fish when they are still on fire roasting them, one side is eaten while the other remaining is still on fire with blood.

**P4;** that’s what they call real life of a fisherman or hunter.

**P4;** and sometimes they compete who can make it first.

**P5;** and that’s the real Jonam man.

**F2;** so eating raw fish actually.

**P4;**eating the fish when it’s still having some blood in it and they don’t mind ,then next day you start seeing somebody’s abdominal start swelling claiming that somebody has been bewitched.

**F2;** ok,

**F2; what changes in your community or health systems or local government would help control or eradicate schistosomiasis from your community?**

**P5;** first, giving of health education to people in the community regarding how they are to live.

**F2;** community sensitization

**P2;** distribution of drugs.

**F2;** who should give the drugs?

**P3;** social gathering, should be used to sensitized people.

**F2;** they should take advantage of social gathering to sensitize people.

**P5;** somebody had talked about drugs, so the government should supply drugs to people to treat bilharzia.

**F2;** the government should give medicine for bilharzia treatment to people.

**F2**; what else, what of the different hospital system that can help us avoid bilharzia?

**P5;** before even going to the hospital system, this people in the community need to be talk to use pit latrines at all times, avoid open defecation you may find in some homes children are left to defecate anyhow and elders don’t bother cleaning or removing their fecal matters so people need to be sensitized that what they are doing is not right, they need to change.

**F2;** community should emphasized that they dig and use pit latrines. Keep children very well, and avoid open defecation and latrines use, then sensitization and awareness.

**F2;** what of the hospitals?

**P4;** peer group counseling, the health workers should be having sessions to health educate people about the dangers of some activities people are doing. Most time people tend to enter dirty/dangerous areas where they can get bilharzia.

**F2;** so you are thinking health system should focus on health education.

**F1;** health education.

**P5;**if possible ,in tis hospital there should be a facility for testing people for bilharzia when called upon because at most time people normally go when there is signs and symptoms like swollen abdomen for treatment.

**F1;** routine screening for bilharzia

**F2;** yes, routine screening for bilharzia

**F2;** boss you talk,

**F2;** what of the government should do

**P3;** the government should acquire health system of distributing drugs for bilharzia to the community.

**P5;** government should provide drugs.

**F2;** issues to do community treatment, the government should make sure there is that provision for treating people.

**P5**;they should supply of tap water(safe water) in areas where there is high risk of getting bilharzia and provision of strict laws to people so that people can fear and adapt some behaviors like public health officers use to move from home to home checking latrines and others made people to change and once there is laxity from the government ,people will continue defecating in the open places like on the raods,in towns where there are areas where people just go and urinate behind buildings and the police officers just look on, without any action.

**F1;** law enforcement

Mod; am suggesting that we try to raise up our voices because we are having a lot of noises from metal work going outside, so we end recoding a lot of noises from outside.

**F2;**one thing he was talking of the government should come up with a law and enforced laws that every home must have pit latrines and provision of safe water, if people have safe water within their reach, they will minimize the risks of going to the water bodies.

**P5;** there is one thing that the government has not taken action are places in towns where there are areas where people just go defecate and urinate behind buildings and the police officers just look on, it’s like something normal and government does not take any action, so when they enforced some laws this things will be controlled.

**Mod;** so what do you want the government to do?

**P5;** monitoring.

**Mod;** so I was looking at if you monitor or enforced laws what have you provided to the community, so we have to look at the positive side from the government to constructs public toilets so that if you want to ease yourself you can go the pit latrine or toilet and then you can enforced the law and monitor the usage of the toilet otherwise it will not be possible.

That’s what happened in Kampala where KCCA came up with laws of no open defecation and urination and they were charging money for the toilets, people had to tell them hey you don’t want us to urinate any how stop charging us money for the toilet because we don’t have money to pay and construct public toilets in different parts of the city so had to stop charging money and employed people to maintain the place and people can access it for free and its always clean.

Likewise the government can construct for us public toilets or pit latrines within the town for the public to use to avoid urinating behind bars, buildings and any other non-gazette area.

**F2;** so any other changes or suggestions?

**F2; has your family ever discussed use of praziquantel or any ways to prevent schistosomiasis? If they have what are their opinions?**

**P2;** such discussion most times comes when the government has started distributing drugs for bilharzia.

**P4;** to add on what he has said, as a family you should have rules and regulations to guide the young ones

That you don’t need to defecate anyhow, use the pit latrines and you have to construct and use the pit latrines otherwise the child will start defecating behind houses. As for me I have put that regulations and constructed pit latrines.

**P5;** you have asked what is disturbing us here in Jonam, in our families, our area here many people including me people don’t think of bilharzia being one of diseases disturbing us, and no one thinks of some ways of preventing bilharzia, the only time people think of Bilharzia is when the government comes with some program of drugs distribution that’s when people think of the disease.

Even when a person is having some signs, he cannot even remember that I used to do some activities that could have probably led me into getting the disease. People even don’t think that open defecation of children at home can also lead to getting bilharzia. People only know that bilharzia only get fishermen

**F2;** to say they don’t talk about it at all.

P4; there is nothing completely, no complain at all.

**F2;** so all they think about are the fishermen and those who don’t fish are adamant.

**F2; who is most important is deciding if a family member comes in contact with schistosoma mansoni infected waters or receives praziquantel for treatment of schistosomiasis? Why do you think that person is important?**

P2; in every family?

**F2;** yes every family.

**P2;** decision from the parents.

**F2;** parents, which one exactly your mother or your father?

**P2;** both of them.

**F2;** why do say the parents?

**P2**; for example for me being a man, I may love fishing they can tell me to stop fishing it may increase bilharzia from entering your body and should take medicine when having abdominal pain.

**P5;** talking about river or talking about drugs whether the drugs have come or not is the person who is always heard is the male parent. He is the one to decide, he can tell the children that I don’t want these things and even about illnesses /diseases are always the men. Women most times whatever they say ,they don’t follow them and even taking of the medicines if you a man don’t follow it up, no one will take the medicine including many women who fear the drugs saying these drugs normally disturb them and when they are given to go home with drugs, they are thrown aside and not taken.so you as a man should follow up starting from the woman and her children you watch them take the medicines otherwise the woman will make the whole family not to take the medicine.

**F2;** one said it’s all the parents because they oversee the home.

The second responder said the fathers, he is very specific the fathers.

**Mod**; and the reason is because people in a family or members tend to adhere to the instructions of the fathers than the women.

**F1;** they don’t bother and...

**F2;** and they fear him most.

**Mod**; yes.

**F1;** that’s make them to take the medicine

**Mod;** yes

**F2; who should be given the praziquantel?**

**P5;** they should start giving everybody.

**Mod;** except...

**F2;** except children from 5yrs below?

P4; from six (6) years below.

**F2; who should not be given the praziquantel?**

**P2;** the pregnant mothers.

**F2**; and why

**P2;** their pregnancy may be young and may results in abortion because the medicine is too strong.

**F1;** pregnant mothers

**F2;** pregnant mothers because the medication may affect the unborn child.

**F2**; any other suggestions of who should not take?

**P4;** the young children from six (6) years below because of the stipulation of the age that they should not take as it may claim their lives because of the strength of the medicine.

**F2;** children below six (6) years

**F1;** what was the reason.

**F2;** they should not take because the medication will be too strong for their age.

**F1;** below six (6) years

**F2;** yes, he said below six (6) years.

**P5;** then people who are very sick or ill should not take because the medicine is so strong that if given to them, it may finish them up.

**F2, P2, P3, P4 ;**( laughing…..)

**P4;** they may dig a grave for real!

**F2;** hmm, who do think should not take?

**P1;** young children between one (1) to six (6)years of age, yes.

**F2;** ok

**F2; Are there any reasons why you or your family members or community should one not take praziquantel?**

**P2;** there is no reason as to why somebody should not take this medicine because this medicine helps when you take it you add some days.

**P3;** transfer, for example if someone has been renting here in Pakwach and gets a transfer to say in Paidha, he or she will miss the drugs.

**F2;** we are saying at you or family member having any reason for not taking medicine.

**P3;** when am sick

**F2;** if he is sick or any other family member is it can prevent them from taking the medicine.

**F2;** what do think can prevent you or your family member from taking this medicine?

**P3;** the medicine is supposed to be given to everybody, but some people do fear.so fear can make that person not to take the medicine because of the side effects.

Secondly, the people who normally distribute this medicine want you to come and take the medicine when they are seeing so most people end up fearing to go and swallow the medicine.

Thirdly, the instructions for the taking of the medicine where it requires you to take the medicine after eating. Some people fear to go for medication because they have not eaten. May be until there is a change in monitoring whereby the medicines are given to the mothers to give to their families and to make sure that the family members take the medicine after eating and she is accountable for it.

Otherwise when they are given medicine to go with at home some people will not take the medicine from home and that is what happen at most time.

**F2;** so there is fear

**F1;** and for any reasons why you or your family members or community should not take praziquantel?

**P5;** I said there is no reason. There is no way how a family or my family can be blocked from taking the medicine. But in other places some people even don’t take this drug because of their religious whatever; when they are giving drugs they say their religion they don’t take medicines.

**F1;** religious practices and believes.

**Mod;** any cultural problems there?

**P5;** and even illiteracy, there are some people are not even aware that this disease exists, and such people when things get into this, they look at it as a joke.

**F2;** ok, what can stop you from taking medicine?

**P2;** nothing. Unless when am not around during the time of distribution.

**All participants; F1, F2, and Mod; (**laughing….)

**F2;** will you take when you are there?

**P2;** yes.

**F2; Are there any reasons why a person should not take or not be given praziquantel?**

**P4;**that is a good question, and this will depend on the condition of a person for example a person may be sick and you can see the condition is not good and if you are to give it may worsen the condition so you don’t give because of condition of the person.

**F2;** one of the reason is when the person is sick, ill bed ridden.

**F1;** do not take.

**F2;** any other?

**P3;** when the person is below six (6) years.

**F2;** when the person is young below six (6) years

**F2;** another one?

**P5;** a person who is pregnant.

**F2;** somebody who is pregnant should not take, why?

**P3;** to avoid premature babies.

**P5;** that’s what I asking myself why pregnant mothers should not take, and in our sitting now I will come to know.

**P4;** what I heard is that when a mother is given this medicine, it reacts with the body and sometimes it makes the baby to come out before the estimated age. (Premature birth).

**P3;** what my teacher told us in class was that this drug should not be given to pregnant mothers because it can cause premature births.

**F2;** ok. It causes abortion and premature birth.

**P3;** yes, premature birth.

**F2;** ok.

**F2; Access to medications like antimalarial drugs and drugs like praziquantel might be a problem. If it is a problem to you or your family, what are the reasons for this problem?**

**F2;** ok, let me ask like this, is getting this drug a problem to you and your family?

**P4;** it’s a problem.

**F2;** can you get antimalarial easily?

**P3;** yes.

**F2;** what of the drug for bilharzia?

**P3;** I do swallow.

**F2;** not swallowing, but are you able to get it easily?

**P3;** yes, from village health teams (VHTs).

**F2;** where do they get it from?

**P3;** they get them from the health centers.

**P5;** for bilharzia, it’s always hard to get most time we get it when there is government program for distribution of this drug.

**P4;** you cannot get it.

**P5;** and even when you go to the health centers you will not get them there.

**F2;** so the question is why it is a problem?

**F2;** you can start.

**P5;** they are expensive.

**F2**; yes, they are expensive.

**P4;** its only when the government organizes for the program then we can get the drugs.

**F2;** one is that they are not accessible to people because of their prices, they are expensive.

Two, they are only available on program by the government.

**P3;** it takes time to come.

**F2;** ok, it takes time to be brought

**F1;** delay in supply.

**F2;** any other reason why this medicine is a problem to us.

If it’s not there let us go the last question which directly to us and each of us will have to answer.

**F2; Do you think being a man or a woman would make a difference in you or your family accessing praziquantel or using praziquantel?**

**P3;** it brings well-being (being healthy) to me and my family.

**F2;**you have started but we are asking whether you think being a man or a woman would make a difference in you or your family accessing praziquantel or using praziquantel?

**P5;**being a man or woman in accessing or using this drug does not make any differences because we always wait for the government programs, so whether you are man or woman ,still you have to wait for the program to get the medicines. Unless we are given another way out on how to get these medicines then it will be easier for us.

**F2;** it can be easy, but the advantages would be there and it’s hard specifically for this one.

**F1;** so is there any differences in you being a man in accessing praziquantel or using praziquantel?

**P5;** there is no differences .That biasness is not here.

**F2;** what are you thinking?

**P4;**from the question asked that whether I think being a man or a woman would make a difference in me or our family in accessing praziquantel or using praziquantel? For me I have found out that there is no differences because we all wait for the government program even if you go as a man to the health center ,where to start from will also be hard, generally it’s hard for both of us to get this medicine.

**P5;** to add on to that, the way we have been discussing it there will be no differences but if we are to buy there would be differences because in most family it’s the men to provide even if a child is to be taken to the hospital still the men who are having job and are responsible can easily access this medicine from where ever they are available and men would have got advantages over women because most women always wait from men to provide for the family.

**F2;** yes ocama, have you got the point.

**Mod;** sorry, I was absent minded.

**F2 ;(** laughs…)

**F2;**he is saying if the medicine where made accessible, readily available then the men would have an advantage of accessing them over the women just because of the gender role that men are naturally to provide ,they will easily get the medications wherever they are available.

**Mod;** irrespective of where he is going to get it from.

**F2;** yes, provided it’s available.

**F2;**because praziquantel is not readily available for both men and women that makes both of them vulnerable but if it was readily available ,men were going to have advantage over the women

**F1;** so the limitation is the availability and access to drugs.

**F2;** what advantages do you as man get in accessing or using “Ambila,” ”baya”, “bitroocide” or praziquantel as we call it?

**P3;**we men normally do manual work, like digging of earth worms used for fishing to make survival easy for and this makes us to get this disease so we need quick access to this drug.

**F2;** men can easily fend; there is that advantage that men can easily fend for the family by doing manual work, labor to see that they work to feed the family.

**P5;**and other is that if we can get access to this drug to take, we men have a lot most time we don’t have anything that can stop us from taking this medicine because women when they are pregnant they are stopped from taking this medicine and for us all day ,all night we can be taking this medicine.

**F2;** with the usage of the drugs, men are usually and you may find women at certain period are pregnant so can be stopped from using the drug but for the man he is always free to use this drug.

**F2;** leaving this one, do we have any other?

**F2;** any other? Boss,

**F2;** what are you thinking?

**F2;** ok.

**P5;** yes another one can be there, if the drug is available men can take more not for any other reasons but to show good examples to the family members, because if you are the head of the family and you are not taking the drug how do you expect a child to take the medicine.so when such things comes you as a parent you should be the first to start so that other people can follow you.

**F2;** ok, this man is like an example to the family like the usage of praziquantel when it’s available once a man takes then family can follow suit so that is an advantage of having influence over the family. So his example of taking the drug or using it not even the children, not even the wife can say no because if the head of the home has taken, so that is an advantage.

**F2;**we have finished ,if there is anything to add you are welcome or any question you want us to answer ,you are free to ask.

**F2;** if there is nothing, thank you so much for your participation.
