# Supplementary material for: Gendered lives, gendered Vulnerabilities: An intersectional gender analysis of exposure to and treatment of schistosomiasis in Pakwach district, Uganda
Source: PLoS Negl Trop Dis. 2023 Nov 10;17(11):e0010639. doi: 10.1371/journal.pntd.0010639 (PMC10684070; doi:10.1371/journal.pntd.0010639)
Supplement: S1 Data — (ZIP) [file pntd.0010639.s001.zip › FGD Schisto Interviews/FGD MALE 46-65 PKH.docx]

**GENDER INTERSECTIONALITY**

**AND**

**SCHISTOSOMIASIS IN RURAL UGANDA**

**TRANSCRIPTIONS AND TRANSLATIONS FOR FOCUSED GROUP DISCUSSION.**

# Abbreviations and acronyms

FGD – Focus Group Discussion

GP2 –Group Two.

F1-Facilitator 1

F2-Facilitator 2

Mod-Moderator

P1-Participant 1

P2-Participant 2

P3-Participant 3

P4-Participant 4

P5-Participant 5

**GP2. MALE (46-65) FGD**

**Introduction:**

**F2;you are welcome for this discussion today, my name is** Noah Okumu and am a facilitator for discussion today and my colleagues are phillip,Ocama Peter our moderator,Nakiranda Salama is our Administrator. I will request you also to introduce yourself so that we can know each other. Am called Jokonyai Santos, Okecha Genaro, Maditho Onyango, Okwong Kabi and Lastly Nyabongo John .Thank once again and feel at home.

**F2;** we are aware that this is the second group and from 46-65 years. And we are going to start with the men, and the first group we have finished with them, the females. We are going to have questions about twelve (12) of them exactly and all of us I think will answer one by one.

Your thoughts or suggestions will give us what you see, how you see them and your experiences.

**F2; so our first question is what activities do you or your family or relatives perform that might lead to infection with schistosomiasis?**

**P1;** the work that are done at family are; the first is fishing by my children and secondly fetching of water from the river, those are what I have seen been done.

**F2;** so, fishing and fetching of water for domestic use eeh.Water is used for home use?

**P1**; yes.

**F2;** yes you had also taken up your hand.

**P2;** the work that we do as he has said I would add secondly is cleanliness, you must have pit latrines, drying rake, rubbish pit and the compound must always be cleaned.

**F2;** (clarifies...) we are talking about activities do you or your family or relatives perform that might lead to infection with schistosomiasis?

**P2;**the main activity is fetching of water from the river for bathing as sometimes there is no money for buying tap water**.**

**F2;** fetching water and specifically for domestic use.

**F2;** is there any other activities they do? Or it’s only those two.

**F2;** ok, aya(Alright). Eeh you have something?

**P4:** am still seeing this activities that can lead to getting of bilharzia disease are like this going to the river to swim and sometimes fetching water where you will find it’s not boiled and they drink it just like that and sometimes not even being filtered so those are some ways of getting this bilharzia disease.

**F2;** ok.yes.

**P2;**let me talk a bit about this swimming ,those days when we were still young children would go swimming at ten o’clock in the morning and would come out of water at midday, we would have generalized body itching.

**F2;** he is talking about swimming and specifically about the time at around ten to midday.

**P3;**let’s look at rainy seasons were would a lot of swamps or collected pool of water in which children would go to swim and would even defecate and go back again to swim, and this would lead to spread of the disease.

**F2;** open defecation,

**P3;**and secondly this young boys who move at night also do defecate anywhere and would not think of pit latrine and all fecal matters are taken to the river by water runoff, hence leading to the spread of the bilharzia disease.

**P3;** they move at night and defecate anywhere.

**F2;** open defecation. Ok.

**F2; why are men more likely to be infected than women in some communities?**

**P5;**this is very true, because we men are always in water both day and night, pushing of the boat to the river will need you to enter and sometimes its dirty ,thick with water weeds and that’s why it’s more in men.

**F2;** for men their life is more depended on water where they go fishing both day and night and that’s why they more likely to be infected than women.

**F2;** yes

**P3;** for me I want to talk about what happened to me,

**F1;** this one is talking about life experience

**P3;** there was a young girl who was staying with me, went and fetched water from the river and during that there was a lot of water snails and collected some in the jerrycan,boiled it for me to take a bath at night as I was taking a bath I felt something on my head and my back, I called for light and found out that they were snails but every these snails landed on got burnt and whenever I take flagyl ,I normally get severe reactions on my body with swelling and itching of my hands meaning that these snails when they are harboring these bilharzia during this time of diluting hot water with cold water, this warm water can makes this worms to become more active to penetrate/enter our bodies.

**F2;** fetching water for domestics use. Whenever you have fetched water from the river, you are advised to leave it under sunshine for the whole day because when fetch it and use it immediately; you easily get bilharzia even at home.

**F1;** how are the men now getting involved?

**F2;** yes that will explain, they fetched water for them and bathe with it at home. Then I don’t know.

**F2;** yes Jokonyai;

P1;another thing I have seen that makes men more likely to get the disease than the women is that we men like bathing in the river most time than the women.

**F2;** so the men like bathing direct from the source

**F2;** F1, P2, P3&P4; (laughs…)

**F1;** that’s very true.

**F2; yes...**

**P5;** to add on to that us men normally when thirsty drink this water directly from the river whenever we are fishing and this increases the risk of getting the disease us than women.

**F2;** so they use the very water they are in.

**P2;** some women don’t cook this fish and other food properly adding bilharzia on bilharzia.

**F1, F2** ;( laughs……)

**F2;** yes

**P4**;aah,to add on that ,what makes us and other young men get this bilharzia because of the activity we do in the water we have some fishermen who do deep diving for about one to two hour under water to mine or collect big snails use for fishing as fishing bathe.

**P3;** under water.

**F2;** what! He keeps air for all this time

P4; he keeps diving to collect the snails and comes out to pour them on the boat and dives again,

**P3;** and comes with snails placed between neck and the shoulder, and others grabbed on his chest. That’s what we have been doing for many years.

**P2;** we do that to feed our family.

**P4;** so looking at the delay under water and the collection of these water snails makes these worms /bilharzia to enter in their body.

**F2;** exposure

**F1;** do we call that one snail mining?

**F2;** it’s different this is deep diving.

**Mod;** yeah, that’s different.

**P4;** and that’s why most of them always have swollen/distended abdomen and when you look at their skins is always dry and whitish.

**P3;** some are dead.

**F2;**the type and there is another type of snails that stay in the deeper side of the river and it seems to be more dangerous than those at the sores, so when they go deeper they encounter that type of snail that spreads that type of bilharzia.

**Mod;** sometimes it can even cut you because the edges are sharp.

**F2;** so they pack them here on the neck and carry some on their chest.(Laughing….).

**F2, P2, P3&p5;** (…continues laughing….)

**F2;** I don’t know how they swim coming back?

**P1;** packed from down upward, Oh, this system! We have come from far.

**P3;** we did that.

**F2;** so you are all deep divers for “koppa” bigger size of water snails” use as fish bathe.

F2; ok,

**P3;** and another one is, there are some groups of wise people who came looking for these small types of snails for making chicken feeds has made us also getting exposed because you have to enter deep in water step on them and start scooping these snails which are the very carrier for bilharzia.

**F2;** that’s snail mining now

**P3;** it’s still being carried out in Panyimur even now, but here we have stopped the activity.

**F2;** yes

P4; but people still go there to mine from Panyimur

**P3;** they do it secretly.

**P3; (**laughs…)

**F2;** so there is snail mining also among the men! That exposes them more.

**Mod;** they value getting money.

**F2;** more of economic bit of it

**F2;** ok, is there any other activity that we men do?

**F2;** if there is nothing, Aaah.

**F2; why are women or their children more likely to be infected in some communities?**

**P2;** children its swimming and for the women it’s mainly fetching water.

**F2;** for children is fishing, swimming any time they are in water and for the women fetching water.

**F1;** children love swimming

**F2;** for the women, fetching water.

**P3;** are you asking about the children and women?

**F2;** yes, children and women, why are women or their children more likely to be infected in some communities?

**P3;** men are women’s porters because they collect all the money from men after fishing or coming back from the river.

**F2; (**laughs...), so what of the women and what do you think about them?

**P3;** the women are always asking for money for jerrycan, bottles of water for solar water disinfection, have you seen. All problems are on men

**F2;** so meaning that women cannot get that disease?

**P3;** yeah, most time it’s the men and for them they just goal keepers.

**F2;** Ok,

**P1;** I have seen that women can get this disease more easily than men because our women like washing clothes from the river.

**F2;** ok, washing clothes.

**P5;** like for children, I have seen that they can get it easily during the raining seasons, they tend to play in this runoff and with open defecation; they can get exposed to this disease.

**F2;** Hmm, women go washing and the children can go on open defecation.

**F2;** but open defecation, do you think it’s only the children?

**F2;** P1, P2, P5, and Mod ;( laughter’s……..)

**P4;** for them they first start with opened defecation and once there is pool of water, they start playing in it.

**F2;** OK,

**F2; what changes in lifestyle can you or your family make to prevent you from getting schistosomiasis?**

**P4;** for me at my home I have decided that no going of children to the river to bathe instead they should fetch water boil it and use it for bathing.

Secondly, cleanliness must be at home, and once a child has defecated in the compound, the fecal matter must be taken to the pit latrine immediately because once rain gets it there, the whole compound will be contaminated and that can bring diseases at home.

**F2;**ensuring they minimize access of water to children who go swimming and the other hand sanitation in the home, the issues of opened defecation ,they have to use the pit latrine and that’s according to what he does at his home.

**F2; yes**

**P1;** my suggestion to the ways of preventing bilharzia disease from my home he like right now the government has brought for tap water, why then can’t the government supply us with free water in every family so that we can use it for drinking, washing of our clothes so that this issue of going to the river by our children should not go and my madam can stop because water will be at my door and this bilharzia will be over.

**F2;** (clarifies..) am thinking that you will have to give me the same answer again, but now we are talking about your life style, what changes in lifestyle can you or your family make to prevent you from getting schistosomiasis? This is yet yours.

**P1;**I have seen it this way, going to the river this bilharzia its strength is more when the sun is up and in the morning they are weak, therefore our women has to change the time of fetching water.

**F2;** changes in time to access water, at least early morning.

**F2;** Hmmm

**P3;** true. You know during the time of our mothers, they used to fetch water very early in the morning when these worms are still down and the top of water still cold. The worms where there but we were not thinking of them like these days. And now our current women don’t want to go to the river early because they say fear hippopotamus so the easiest way is to bring tap water. But still our young boys who want to go fishing will still carry it for us, because they will have to stand in water and if they would be on the boat, it would be better.

And there are other bad fishing methods call “korokota” where you stand in water for long time pulling the nets from the water, other fishing method where you have to remove “pomo” (the fishing bathe) like earth worms “lanyata” from mud or under water hyacinth and again the washing of nets which requires you to stand in the water for long time. I wish they could go fish or stand for short period of time, it would be better.

**F2;** reducing on the exposure time is what he is trying to hint on.

It goes back to the first person and also there is other things he added things to do with the mode of fishing and washing of nets.

**Mod;** yeah.

**Mod;** and you could still add these different modes of fishing where you need to be in water for long time guiding the net, and pulling it back to the boat to remove the fish the “korokota” unlike the other fishing mode/style where you on the boat.

**F1;** the mode of fishing

**F2;** and also another thing, the washing of the nets and you have to wash them in water.

**Mod;** so under the modes you can talk of the washing and others….

**F2;**ok.

**F2;** maybe we go to the next questions.

**F2; what changes in your community like we are from different villages Mubogo central, north, south and puyoo or health systems or local government would help control or eradicate schistosomiasis from your community?**

**F2;** Yes

**P2;** for me I have seen it this way,

**F2;** let start with the family!

**P2;** yes, like the government has come with policy of everyone/family must have pit latrine to reduce the following of fecal matters to the river, and people are digging pit latrine unlike those days people were doing opened defecation but now it’s not being done unless at night that’s when somebody can do it.

**P2;** but for the water you cannot stop, it’s hard because our lives depend on the river.

**F2;** but what can you do?

**F2;** so one thing is the issues of sanitation, ensuring every home must have latrines

**Mod;** promoting the use of pit latrine

**F2;** promoting use of pit latrines

**F2;** yeah

**P3;**what we can do, and there was a year I have seen is that there used to be giving of bilharzia drugs at schools to school children and even in the community and for me who used to fear drugs ,I gained confidence and took the medicine because the worms had stopped me from eating fatty meat and fish, pawpaw , posho from maize and sweat potatoes but now I can eat them and there is a big change after taking the medicine .And another thing is the drug the prevention is better than cure eeh we are not going to use it because we survive on the river.

**F2, F1, P2, P1 ;**( laughers...)

**F2;** mass drugs,

**F2;** yes, Jokonyai

**P1;** what I have seen from my village, there is changes because many people once their stool samples are taken for have been testing negative for bilharzia and it’s because the government has been giving us a lot of health education over the radio or sometimes they organize within the community and they come to teach people through a lot of changes I have in people.

**F2;** Ok

**F2;** Something on sensitization and health education

**P4;** what I have seen that can help us prevent bilharzia for us right now is that health education and the number of children who used to go for swimming has reduced as they play at home most times. This has made number of people who are having bilharzia has reduced because activities like swimming and deep diving where you would stay for a long time in water has stopped.

Another thing that has reduced the strength of bilharzia is “baya”,(the praziquantel) that government is giving people to take has brought changes in the life of people.

Secondly, still on this health education in the community where people have been attending has made changes in the level of cleanliness, many people have constructed pit latrines, bathing shelters and whenever a child passes a stool, they parents or care takers would clean the fecal matter and take directly to pit latrine which was not the case of those days, so those are some of the things that has brought changes in us.

**F2;** ok

**F2;** so there is mass sensitization coming up, mass drugs administration coming up and the issues of sanitation at household and community level has improved.

**F2;** yes.

**P5;**for me I have seen that, the strength has reduced but not so much and for killing it, I have seen three things;

First, the government should increase the number of VHTs (Village Health Teams) and other people who can give health education in the village should be there.

**P5;** secondly, I have seen that,.

**F1;** community empowerment,

**F2;** adding the number of VHTs.

**Mod;** adding the numbers of the VHTs and training of some people who can train others

**F2;** training of people who can train others.

**Mod;** training of trainers

**P5;** secondly, I have seen that the government is bringing drugs but they are leaving period to pass too much.

**F2;** and they are leaving the Period...

**P5;** sometimes they take over three to four years without using the drugs and that is affecting the effectiveness of the drugs. I wish it is given monthly or after 3 months apart, this bilharzia disease would have been cleared.

Thirdly; as they had said that for us we are fishermen, meaning that we cannot be separated because even if you have given us the medicine and have taken it, we shall still go back the river and the worms will still enter in us, therefore if the government would invent/discover or make for us a cream/lotion which you can smear on your body

**P5 & others ;(** laughs…)

**P3;** yes,

**P5;** to prevent the spread and entry of the worms into our body and that is one I have seen can help us prevent the spread of bilharzia.

**P3;** paste…paste…

**F2;** things to do with research**,**

**Mod;** yes

**F2;** something to do with research and some other preventive measures especially the fishing force may be they can get something to apply on their body like a prophylaxis.

**Mod;** more of mosquito’s repellants

**F2;** he is giving example of mosquitoes repellants.

**P3;** the greasy one

**F2 & other participants ;(** laugh…..)

**F2;** yes, there was a point that you had given and I told you that you will share with us later in this point, about….

**P1;** I was talking about the prevention of bilharzia in our families since they have brought tap water, if the government can implement it because we are poor sometimes buying this water is hard and many taps have been closed because sometimes they would bring a bill of about twenty or thirty thousands and yet you don’t have any source of income and if the government can supply us with free water so that our children and women can stop going to fetch water ,wash clothes and if we are to tap water it’s hard for us.

**F1 & F2;** access to safe water.

**F2;** safe water chain

**Mod;** you bring this element of the water and the bill

**F2;** yes, the cost is high and the few taps which are there have been closed.

**F2**; yes somebody has taken his hand?

**P3;** yes, for me I want to repeat something on the drugs which I have seen when HIV/AIDS had just entered, there was no drug, the strength was too much and many people died. When the drug was discovered, many people started taking the drugs and others have stayed on drugs alive for over thirty years meaning that the drug is working and if the drug was not there, many people would have died. But it’s still being found among people just like bilharzia were we say when you go to the river ,you will get it and when you are back home ,you take the medicine that’s good.

All these is like cleaning rubbish at home, whenever you have trees at home ,the leaves will always fall down and you clean it, wind blows and again still you will continue cleaning and that is life where you have to keep taking care of it.

Your body can get dirty, you wash it and you cannot stop bathing because when you do so just after three weeks, you will smell.

**F2 & other participants ;**( laugh…)

**F2;** he has given example of HIV/AIDS.

**P3;**so if we would have this medicine and use it after fishing or take after some days ,weeks or month it would be fine and would have help us a lot to at least push some three ,four days ahead.

**F1;** constant supply of praziquantel

**F2;** its accessibility. Readily available because they are exposed at all times and need it more of prophylaxis.

**Mod;** I think if they would come up with a prophylaxis treatment or even vaccines for it would be better just like vaccine for typhoid ,cholera where even if you have taken contaminated water ,you will be protected.

**F2;** ok, let’s go to the next question.

**F2;** may be let me ask like this, are you all married?

**All participants;** yes.

**F2; has your family ever discussed use of praziquantel or any ways to prevent schistosomiasis? If they have what are their opinions?**

**F2;** you can start**.**

**P1;** like family, this issue of this drug is very difficult, that is why this plan of giving the medicines and taking it immediately was the best option because of fear especially the women, always refer to that drug being bad and they fear to take it that is what is happening in my family. Like when they are given medicine to go home with, they don’t take it and that’s sometimes I quarrel with them claiming that the drugs reacts badly with them.

**F2;** asking whether you have discussed as a family about the use of praziquantel or ways to prevent schistosomiasis (bilharzia) or not, and what have you discussed? And not this type of discussion with the VHT or any other health worker.

**P2;**yes we have discussed and I told them that bilharzia is a bad disease, for example for me I have vomited blood two times all related to Bilharzia so I was advised about the medicine and took it since 2008 up to now I have not got any problem again.

**F2;** what advise did you give them?

**P1;** the advice I gave them was to take medicine to prevent this disease.

For the young ones who are still fishing should always take medicine because for us we were fishing without taking any medicine. And people are taking medicines, just the other time the medicine was not enough for the people.

**F2;** now let us give example from our own homes.

**P5; t**he discussion as a family, we have discussed it and what I have found out is that some people after discussion, they agree with you but later go their ways just like what this big man was saying that every is given their own medicines and you find others not taking the medicines despite the discussion about the dangers of this disease (bilharzia).

**P2;** just like from my home, you will find madam is worsening the situation, telling everyone how bad the drug is and how severe the side effects were to the children making them not to take the medicine and this has been giving us great challenges at home.

**F2;**yes.

**P3;**you know we are here ,we are Africans and many people have died without taking this medicine because Africans despises things more so the men ,they wait until they are badly off that’s when they will visit the hospital.

The women are fearful, they react very fast and get treatment but for the men they persevere a lot, thinking of getting better soon until they cannot get up. I was one of those stubborn fellows who used to refuse going to the hospital until my brother here talked to me and I changed my behaviors not going to the hospital.

So without serious sensitization and health education people will not change.

**F2;** ok, thank you.

**F2;** so in the family they have shared about praziquantel and the most especially about praziquantel they have mixed opinions there are those who have accepted, those who have rejected and others who fear especially women who even instill fear in children.

**Mod**; others **e**xaggerate the side effects of the drugs, that the child reacted badly and try to make the drug look bad..

**P2;** Just as I have said that I reacted terribly at first but now am fine, I don’t even feel anything.

**F2;** ok,

**F2; who is most important is deciding if a family member comes in contact with schistosoma mansoni infected waters or receives praziquantel for treatment of schistosomiasis? Why do you think that person is important?**

**P5;** the male head of the family.

**F2, P1, P2, P3 ;**( laughs….)

**F2;** the father.

**Mod;** the study is becoming interesting.

**F2;** why do you think he is the one important?

**P4;** because when any child refuses to take this drugs, it’s the responsibility of the father/husband to make sure this child takes the medicine. And it’s not only drug for bilharzia that people refuses to take but other drugs as well, so the head of the family has to make sure everyone take their medicine as prescribed, because may at times you will find a sick person would go to the health center to get medicine and take it for only two days as they get better like this ,they tend to abandon these drugs so it’s the head of the family to make sure everybody at home complete their doses of drugs and if you don’t ,this medicine will not be taken.

**P5;** it’s the husband or the father because he is the one who brought his wife at home and they got children but still he is the one who takes care of them all in sickness and death of any member of his family so to prevent any kind of behavior or sickness from getting worse which may make him spend a lot of money in an obvious way and yet he can talk and change the situation and saves his money, that makes him the important person and the decision maker in a home.

**F2;**any other suggestion which is different from his?

**F1;** the man,

**F2;** the man supports his homes in medications, eating, the burials,

**F1;** then the family head, the one who has brought the wife.

**F2;** brought the wife and the children are his.

**F1;** fountains of honor

**F1, F2, Mod;** (laughs…)

**F2;**yes.

**P3;** for me the speeches made by these two members are good but the main teaching goes to the women because they are the nurses because we men most time we never at home or can have a journey which can take you away from the family for a week, one month or maybe he is working in Kampala and leaves the wife at home with children where she has to care of the family but for the orders still comes from me even if on phone.

**F1, F2 &P3;** (laughs...)

**F2;** so it still goes to the fathers.

**F2;** he still supplementing that women should know how to handle these things at home but still the authority comes from the man even if he is away from home for a month.

**F1, F2 ;**( laughs…)

**P5;** so we have found out that the mothers are like nurses who start keeping children from baby up to when they are older and when you are going to start a home, you should not get a woman with weak mind.

**F2;** ok let go us to the next.

**F2;** who should be given the praziquantel?

**F2;** (explains…more on the question), who should be given the drug for bilharzia, is it the children, mothers, fathers or the fishermen, pregnant mother or sick people?

**P3;** everyone has to get it.

**F2;** everybody.

**P3;** yes, because the child will swallow his and I will take mine.

**P1;** everybody under the recommended age group

**F2;** every recommended age.

**P1;** I don’t know about the pregnant women because we are saying everybody, maybe you can tell us more.

**F1;** everybody who is eligible.

**F2; Are there any reasons why a person should not take or not be given praziquantel?**

**P4;** am suggesting that the newly born or babies from two to three months should not take

**F2;** ok,

**P4;** but everyone of good age should take this medicine.

**F1;** three months up to..?

**F2;** newly born, he said two to three months, very young one/ the infants.

**F2;** any other?

**P1;** sometimes back they were saying the pregnant mothers should not take but now I don’t know.

**F2;** (laughs…), the pregnant mothers.

**F2;** another?

**P5;** I have seen that everyone should take it but I doubt whether the unborn can get this medicine from her mother’s womb.

**F2;** they don’t get it**.** (Laughs…)

**P2;** am asking about this drug, whether the manufacturers don not put any instructions on the use of the medicine?

Giving instructions about who should take or should not take the medicine.

**F2;** we are asking your suggestions about who should not take this drug.

**P1;** who should not take this drug?

**F2;** so you have suggested the pregnant mothers and the young babies.

**F2;** do you have another?

**F1;** even if you have taken alcohol.

**F1 &F2 ;**( laughs…), ok.

**F2; Are there any reasons why you or your family members or community should one take praziquantel?**

**F2;** yes,

**P2;** just like we had talked before about the fear of having abdominal pain, diarrhea and vomiting after taking the medicine makes people not to get the medicine.

**F2;**fear of the side effects.

**P1;** sometimes shortage of drugs were some villages may get and others misses.

**F2;** first imagine that the medicine is coming, and are there any reasons why you or your family members or community should not take praziquantel?

**P1;**rumors or listening to wrong information about the drug from fellow women.

**F1;** threatening rumors,all about adverse side effects.

**F2;** rumors like on what?

**P1;** rumors about how bad this drug is and how terrible are the side effects like severe abdominal pain creating fears.

**P3;**there was something which was happening rumors, they were giving drugs to people and the side effects were severe abdominal pain that makes you roll as if you’re going to die and this is what I saw brought fear to people who have not yet taken the drugs.

**F2;** so fear also, but still fear of the side effects.

**P3;** so I have been covering it with something good

**P1;** coating the drug with something sweet to remove the bitter taste that remains in the mouth.

**F2;** I think we can go to the next.

**F1;** what of something like, for me am a born again and don’t take medicines

**F2;** the issues of religious affiliations,

**P5;**that you don’t need to use medicines when you are sick.

**P3;**the issues of religious beliefs was happening when the full gospel Pentecostal churches had just come ,they were saying no taking of both local herbs and manufactured drugs but after having enough knowledge, they started taking the medicine because at first they were ignorant.

But I have not heard of any at our place here except one in Okuru County that they are having one religion that does not belief in taking medicines.

**F2;** God has overcome / won Satan,(laughs…) ok

**F2; Are there any reasons why a person should not take or not be given praziquantel?**

**F1;** Eeh ten**,**

**F2;** yes ten.

**F2;** yes we can talk on very fast

**P2;**am suggesting that ,when the time of the medicine has reached and the person is very sick or very ill should not be given because if you are to give the person medicine ,he can even die because the medicine is too strong that’s how I have seen it.

**F2;** the ill someone who is ill at the time of administration of the drugs

**P3;** the question that you have asked**,** for me am thinking these way, when we were still young boys growing up in 1964, we had four boys with swollen belly (abdominal swelling) and they were saying it was poisoning and yet it was this disease Bilharzia, one person out of the four was taken to Gulu and was treated and the abdominal swelling disappeared and the other two died leaving one to struggle with liver disease as said before but later also died (the late Opasi).But John the son of Asumpta and Ongeya son of Javuru died because of ignorance.

**F2;** so the way you have seen who should not be given this drug or who should take?

**P3;** this drug should be given to a person who has been tested and found to have the disease and also to those who have tested negative as well to protect their body.

**F2;** now who should not be given this medicine?

**P3;** Heeh who should not be given…

**F2;** yes

**P5;** for me I was thinking of two or three people, first very sick person, secondly pregnant mothers because this drug can cause abortion or miscarriages and thirdly the young babies.

**P1;** for me am looking at a person with Tuberculosis (TB) disease who is very weak.

**F2;** he is trying to be specific TB patients.

**F2;** ok, let’s go the next question because our time has gone.

**F2; Access to medications like antimalarial drugs and drugs like praziquantel might be a problem. If it is a problem to you or your family, what are the reasons for this problem?**

**F2;** yes

**P2;** have seen at most time when you go to the hospital ,they write for you medicine to go and buy, now when you don’t have the money it becomes hard to get the drug.

**F1;the access or**

**F2;the availability ,not readily available.**

**F2;** yes.

**P1;**what I have seen or sometimes I tend to have problems with the supply of the drug which is low and the consumers are high and this cause some people to miss drugs because you may go at a time when the drug is over simply because few drugs were supplied to meet the population.

**F2;** the quantity, supply does not meet the demand of the population.

**Mod;** supply of the medicines does not meet the supply and that comes back to us the leaders in the district.

**F2;** if you are to get from outside, do you get the drugs?

**P2;** you can get

**F2;** so why can’t you get it.

**P2;** you can get except the high cost that cut you off.

**F2;** the high cost of the drugs

**F1;** Hmm.

**F2; Do you think being a man or a woman would make a difference in you or your family accessing praziquantel or using praziquantel?**

**P2;** the advantage is when you have the money to buy drugs and bring home but if don’t have the money to buy the drugs then there is no advantages.

**F2;** so in accessing praziquantel or using praziquantel does not make you man enough?

**F2;** advantage to you

**F1;** to say, is it easy to get the drugs or it’s a big problem to you

**P3;**there is no any advantage of being a man, you are as a burden, the only thing you will be seeing will also problems and too much stress will be the one to make you all.

Your own the children will become your enemy and if they don’t want whatever they will be doing will always be despising things.

Being a man needs you to have enough things, then you can get some advantage and you can give some orders.

**F2;** so being a man, can it help you to get the drug?

**P3;** you can get the drugs when you have the money or you have been supported then you can get the drugs. So it’s too difficult to be a man.

**F1, F2, &other Participants; (**laughing….)

**P1;** for me am seeing it at this point that being the head of the family, you should have something to do which can give you some income, even if doing some odd jobs at least you will have some five thousands to buy some drugs of two thousands and have a balance of three thousands to be in your pocket otherwise without any then it will be a disadvantage.

**F2;** the advantage of being a bread winner give you access of buying medications and aspect of work defending for the family.

**F1;** so, it means they have a better source of income?

**F2;** I think Aaah...

**F1**; is it the income level that makes them more men,

**F2;** more of income level. Income level and other sources of income

**P2;** we are men when we have money. The money we get it from the river.

**F1, F2 &P2; (**laughs…)

**F2;** Aaah, what other advantages do you have?

**F2;** what other things when failed by a woman you can come in as a man,

**P5;** for me I have seen that being a man, makes you have the responsibility of your family .And when the disease has comes to your child ,when you are there the woman has nothing to do with that. It’s you to run to your friends and borrow some money to take the child to the hospital. On the day where feeding is hard, still you will have to look for ways to feed your family either digging some body’s garden to make sure they eat.

So I have found out that being a man, getting drugs to treat your family cannot defeat you because you can pick any of your clothes and run to your friend to help with some money because you know it will be paid back by you.

**F2;** so the virtue of being responsible for the family and being the head of the family,

**F1;** give them upper hand ……

**F2;** give them upper hand to take charge of accessing the medications when they need.

**F1;** and the connections…

**F2;** yes, the connections gives them link with friends and gets on well.

**F2;** eeyo, we are going to end with yours.

**P3;** for me find being a man is good to have a policy, like being an Engineer it’s good to have a helper and being a man when you get a wife who can help you then sometimes it’s easy but if get a woman whose head does not reason well then you have to climb the hills alone.

**F1, F2, Mod & other participants;** (laughs...)

**P3;** that’s why the Alur men normally leave their women and children for a far place, stays there and even die minus looking back at their families because of such women.

**F2;** like the other advantage is like the men have helpers, and those are the women. The supports they have from the women are of advantage where when things are hard these women can support other than you being alone.

**F1;** is like the position of command they were talking about.

**F2;** may be, but no is like when you find a woman who does not support you then you are doomed.

F2; I think if there is nothing else to add, from here we have exhausted all the questions.
