# Supplementary material for: Gendered lives, gendered Vulnerabilities: An intersectional gender analysis of exposure to and treatment of schistosomiasis in Pakwach district, Uganda
Source: PLoS Negl Trop Dis. 2023 Nov 10;17(11):e0010639. doi: 10.1371/journal.pntd.0010639 (PMC10684070; doi:10.1371/journal.pntd.0010639)
Supplement: S1 Data — (ZIP) [file pntd.0010639.s001.zip › FGD Schisto Interviews/FGD MALE(46-65)YRS.PANYIMUR.docx]

**GENDER INTERSECTIONALITY**

**AND**

**SCHISTOSOMIASIS IN RURAL UGANDA**

**TRANSCRIPTION FOR FOCUSED GROUP DISCUSSION.**

**Abbreviations and acronyms**

**FGD** – Focus Group Discussion

**GP2** –Group Two.

**F1**-Facilitator 1

**F2**-Facilitator 2

**Mod**-Moderator

**P1**-Participant 1

**P2**-Participant 2

**P3**-Participant 3

**P4**-Participant 4

**P5**-Participant 5

**GP2. MALE (46-65) FGD**

**F2;** not to waste time we are going to start.

The first thing we are supposed to do is to agree that you have agreed to participate in questioning, answering and sharing your experiences in this focused group discussion.

So you are going to fill these forms here, either in English or Alur language.

**F2;** in this form contain the following; the purpose of the study which is looking at the gender in transmission of bilharzia and secondly gender in the use of praziquantel, “baya” or bitroocide. My name again is Okumu Noah and today you are group two and you will tell us your name and the village.

**F2;**We are going to start with you ,lower the mask down and speak louder so that we can listen your voice very well.

P1.my name is Vincent Okumu from Afoda village Ganda 1.

**P2;** my name is Orieda Rogers from Singilla central.

**P3;** Okumu silvers from kuluber village

**P4;** to say we are only using Sub County not ward?

**F2;** yes.

**P4;** ok, am Alex Owonda by name I come from kaal village Ganda 1

**P5;** Adubango Michael from Cendi village Ganda parish.

**F2;** thank you, and you are well distributed.

**F2;** from here we have Peter Ocama, Salama and Phillip.

**F2;** we are going start answering, one after the other as he continues writing our name tags.

We have twelve questions in total and we are going to give five minutes for each question and answering.

**F2; and the first question is what activities do you or your family or relatives perform that might lead to infection with schistosomiasis?**

**P4;** we start answering?

**F2;** yes. You can start by introducing yourself.

**P4;** I had introduced myself before, I will start with my family as for the people of the lake, our livelihood depend on the lake, fetch and my children do fishing from the lake. And once they have gone I normally tell that they are at risk of getting bilharzia and that is one of the work they do.

**P3**, the work that we do in my family that can put us at a risk of getting bilharzia are; we do farming, and buying of the fish from the landing sites.

**F2;** how do you handle the buying?

**P3;** we still enter the water to clean the fish and others do fishing.

**F2;** fishing and buying fish itself

**P5;** work that can make us from my family to get bilharzia is fetching of water from the wells and some people don’t have latrines, so they end up having open defecation and once it rains, it washes the feaces with worms to the water bodies including streams which we use because accessing safe water/borehole is hard for us.

**P1;** for me one of the things that can make my to get bilharzias is water snails, because they do snail mining and they do that as a mean of getting money.

**F2;** snail mining.

**P1;** I don’t know how to avoid it.

**P2;** I have seen that most time even digging is done by the lake banks, those who go to buy fish still enter into the water and through that bilharzia can enters our body.

**F2;** let’s come back to us men, why do you….

**P2;** maybe I can add something

**F2**; yes, add

**P2;** I don’t know how you may see this, with this flooding all latrines have been destroyed by water and this has brought to us increments to how people can get diseases.

**F2;** how then,

**P2;**the how is because of these broken latrines all the dirt has been carried to the lake and some children and some adults do go there to take a bath from the lake.

**F2;** bathing in the lake

**Mod;** to add on to that with this flood many pit latrines has been destroyed and many people are not using pit latrines so they do open defecation and they feel the water has reached them close to home and end up bathing in it which exposes them to bilharzia.

**F2; why are men more likely to be infected than women in some communities?**

**P2;** men are more prone because most of the heavy work in the lake are done by men like fishing

**P5;** to add on that even digging at the banks is done by men where they may work on that piece of land for even one week and that may put them in a greater risk of getting the disease.

**F2;** so there is farming and some spots are only for the men to go

**F2;** ok, any other reasons?

**P2;** for me the major activity is fishing which is only done by the men which could place them at a risk of getting disease.

**F2;** I think the major activities are those one.

**F2; why are women or their children more likely to be infected in some communities?**

**P3;** for me women are more likely to get this disease because of fetching of water where they have to enter in the lake to fetch.

**P4;** for me I have another suggestion, the way we survive here in Panyimur I cannot agree that its women who are more likely to be infected with this disease because I have seen that both men are women are always in the lake. The women are always interacting with the processing of fish and fetching water at landing sites while the men are always fishing twenty four hours so they are both equally at risk of getting this disease.

**F2;** so on the women side they are always processing the fish,

**P4**; yes, they are always processing the fish and fetching of water, they have to enter there.

**F2;** yes you had another points

**P4;** an attempt, am disagreeing with his points, it’s not that women are always processing fish The only way women are at a risk of getting bilharzia is fetching of water because they do it daily and here in Panyimur, some women are always at the garden digging and if you say they do get the disease from the farms, there I do agree it can happen. But sincere speaking it’s the men who do go fishing and process the fish at most times.

**F2;** who normally process the fish, is it the men or the women?

**P4;** the men. The women only remove the scale from outside on the dry land.

**F2;** what of the washing?

**P4;** that’s why am saying may be in the process of cleaning they can get the disease.

**P2;** there is no way you can avoid women from using water in twenty four hours; they always use water to do many things.

**P1**;for me I have seen that its draw because men bring these fish from the lake and pour them on the dry land and the women start processing it by removing the intestines, scales, cleaning it in the lake and taking to the market.

**P5;**for me it’s not draw because when you take the duration men take using the lake compare to the time women take to buy fish and clean it then back home. There is a very big difference,

**P4;** yes, there is a big difference.

**P5;** for instance a person who goes fishing for about one month in the lake and the one who goes in for thirty or two hours in the Lake.

**P4;** I wanted to know how long does it take for bilharzia to enter into your body, is minutes or hours?

**P3;**it does not matter whether you have taken one month or one day, as long as you have entered into the water and the bilharzia is there ,even if you have ten seconds it would have entered your body.

**F2;**it does not take a lot of time, that’s why sometimes after entering into water ,you may start feeling itching of the lake.

**P4;** so the person who has been in it for one month has got a lot of it now.

**F1;** the moment you enter in water it bites and enter your leg.

**P4;** no problem let go now.

**F2; what changes in lifestyle can you or your family make to prevent you from getting schistosomiasis?**

**F2;** yes

**P4;** what I have seen to change our lifestyle, first we must have pit latrines in our homes.

**F2;** yes

**P3;** for me I have seen that let us change our lives because you struggle to have pit latrine at your home with all other required items at home but there is another person who may not see all these and will go the lake and urinate there, and once you enter the lake, you get infected so the only way out is that we should always have the medicine at all times.

**P2;** what we can do to prevent bilharzia from our families is that, these farming we normally do in swampy places we should be putting on gumboots.

**P1;** I have seen also this talking we are having, if we would also inform some people who lacking such knowledge would be good.

**F1;** sensitization of the community.

**F2;** yes, any other changes?

**F2; what changes in your community or health systems or local government would help control or eradicate schistosomiasis from your community?**

**F2;** yes number one

**P1;**I have found out that there should be enough knowledge on how to prevent bilharzia meaning that the environment must be clean, pit latrines and other things like when they say every day in the morning you should bring water to wash hands and other different things to prevent other diseases.

**P1;**Secondly I have seen that we should at least pay visit to the hospital or health centers to discuss some of the things which has been disturbing us with the health workers so that the government can support us to prevent some of these challenges.

**F2;** so improve on their knowledge on the preventive measures and improving sanitation at house hold level and thirdly, they have to work on their health seeking behavior.

**F2;** yes,

**P3;** let me also add something it may also be similar to what he has said that one time should be allocated to teach community that these worms are there and if health education is done in the community that stop urinating in the water bodies to prevent bilharzia, stop defecating in water and others do open defecation and their feaces may be having bilharzia where when it rains it washes the fecal matters into the lake as some people have their activities in the lake.

**F2;** so that is both side of the government and the health facilities?

**P3;** yes that is on the government and the hospitals or health system.

**P2;** I wanted to add something small that the government if could provide equipment for detecting and testing this disease with signs and symptoms I have heard that it brings abdominal pain and others that equipment should be there to prove that its really bilharzia disturbing and then they can get the treatment that would be good.

**P2;**And if the government can also get the drugs for bilharzia in all the health centers so that everyone who is tested and found to be having the disease should be given immediately because some people when given this medicine to go home with them to swallow ,they don’t take the medicine. So if the government can have this medicines and the testing /diagnostic equipment in this health centers it would be very good.

**F2;** one is diagnostics equipment and secondly medications.

**F2; has your family ever discussed use of praziquantel or any ways to prevent schistosomiasis? If they have what are their opinions?**

**P4;** for me as I have come here, am a VHT we shall have teaching on this, and I will start it with my family and with one or two people who are here I know they join me but as we had discuss here that you may be taking control measures against bilharzia when your neighbor is doing opposite and you become a bad person.

**F2;** we are talking about your own home now, that have you discussed about bilharzia and taking of the praziquantel

**P4;** that one we have discussed and the medicines when we were given, we took them

**F2;** how were they, was it good or bad and what was their opinion?

**P4;**the way I saw it, the children of five to eight years did not seem to like the medicines but older children were ok with taking of the medicines.

**F2;** so now you leave the medicines aside and about the discussion did they welcome the suggestion?

**P4;** about the discussion it was well accepted.

**F2;** let me come to you.

**P3;** thank you, you have asked a very good question and once you have been diagnosed with bilharzia disease be open because bilharzia is not a good disease. For me I have sat many times with my family to discuss about bilharzia and have got the charts about the spread of bilharzia, pinned it in my house and use it for teaching my family that when you urinate or defecate in water and when you have the bilharzia it will enter the water and stay in water snails and once you enter in that water the bilharzia will enter in your body.so the children asked me, daddy should we be killing the snails once we get them? I told them yes, if you can but carefully because when you step on the snail barefoot the bilharzia will enter in your body. And how does it look like? I told them even for me I have not seen them but you can see them using a microscope and they are dangerous. They appreciated it and when ever given medicines for bilharzia they take them but the side effects still affects them and they talk about it that daddy the drug was too strong.

**F2;** now we are talking about our experience or the Congolese called it “expera”

**P5;** so for me am answering that, I have been meeting with my family not only about bilharzia but about other diseases like malaria.so like right now we are talking about bilharzia we have been emphasizing the use of pit latrines and discouraging the open defecation by young children and we teach them how to use pit latrines to prevent such diseases.

**P2;** for me on my own behalf and my family, am seeing the main issue is more on preventive measures. So for me and my family we use to do snail mining but after discussing about the dangers of bilharzia we stopped immediately because it has brought a big problem to my family and it took a lot of money from me. We now employ other people to do the work and none of my family member goes closer to it starting from that time.

**P1;** I don’t think I have a lot to say because I have seen what bilharzia has done to people who have been doing snail mining activities, and when the drugs for bilharzia my family took the medicines and I told them the benefits of medicines and with the dangers of bilharzia they have seen where some died and others having swollen abdomen as a result of snail mining activities, next time when the drug is being distributed, I know they will take it without any problem.

**F2; who is most important is deciding if a family member comes in contact with schistosoma mansoni infected waters or receives praziquantel for treatment of schistosomiasis? Why do you think that person is important?**

**F2;** yes number one

**P1;** the parents

**F2;** the parents. Between them who decides more?

P1;because sometimes when the man is not at home or has gone for a journey, it’s the woman to take full responsibility and when the woman is not around ,the man has to take the responsibility.

**F2;** both parents and the reasons. (Laughs…)

**F2;** yes

**P2**;from my side ,the woman I have has knowledge of medication and the time of giving this drugs ,so most times she is the one take the responsibility.

**F2;** who decides on taking or not taking the medicines or don’t go there, who makes the decision?

**P2;** it’s our responsibility.

**F2** ;( laughs…) yes,

**P5;** am answering this way, in our tradition it’s the man to take most of the important decisions in a family and a woman only takes decisions when the husband is not around.

**F2;** why have you said so?

**P5;**I have suggested that way because we have seen that most times women tend to think more of the kitchen than other things so for you as a man you have to generalized everything and make sure everything is moving smoothly to protect the name of the family.

**F2; who should be given the praziquantel?**

**P4**; a person who has been tested to be having the worms.

**P3;** as per my own opinion with our lake here in Panyimur I would suggest that everybody of the required age should take regardless of whether you have it or not.

**P2;** am suggesting that for those who lives along the lake or rivers should be given as long as they meet the required age

**F2;** the risky population to be given

**P5;** for me I would suggest everybody who reaches the age should be given and the drugs should be enough not be given alone to children and the elders to wait.it should be just like the other time were everybody was given.

**F2; who should not be given the praziquantel?**

**P2;** any person whose age has not yet reach, especially the under five years

**P3**; a person who is very sick or ill because if given with the strength of the medicine he or she may die

**P4;** to add on to that the pregnant mothers

**P5;** even the breastfeeding mothers of children less than six months

**F2;** any other who should not take the medicine?

**All participants;** not there

**F2; Are there any reasons why you or your family members or community should one take praziquantel?**

**P5;**am suggesting that it’s good to take the medicine whether you have been tested or not so that in case you have the worms you will be treated and you will be fine. And if you don’t have the worms yet still you will be protected from the disease by taking the medicine before any sign or symptoms shows up.

**P4;** it’s just like that. A white man said “prevention is better than cure”

**P3;** in order to take medicine, first of all me and my family should know our level of cleanliness how is it.

**F2;** the question is like this Are there any reasons why you or your family members or community should one take praziquantel?

**P3**; in that case what will make me take the medicine is when I know that where am going like the lake is highly infested with the worms and then I will forced myself to take the medicine.

**F2; Are there any reasons why a person should not take or not be given praziquantel?**

**P4;**for I don’t think there is any reason that can make me and my family not to take this medicine unless am too sick or ill to take the medicine.

**P3**; for me what makes me not to take it is because it is hard to get unlike those days were you can get it easily.

You may go to the health centers and it will not be there, so getting it is hard.

**P2**; it is also expensive.

**F1**; what if the drug is there and is there any reason that can stop you from taking the medicine?

**P3;** there is one reason I have seen is that there is some religion that used not to allow people to take medicines.

**F1;** are the full gospel here?

**All participants;** many

**F1;** I used hearing that they used not to take medicines.

**P3;** there are so many churches here

**F2;** is there any other reason? Or these are the main ones?

**All participants;** there are no other reasons.

**F2; Access to medications like antimalarial drugs and drugs like praziquantel might be a problem, If it is a problem to you or your family, what are the reasons for this problem?**

**P2;** am going to answer, this thing is a problem and am going to put it on the government because they have put hospitals but you may find for two to three months there is no drugs and yet people are falling sick almost daily.

If the government and other partners can maintain a constant supply of essential medicines more especially the anti-malarial to these health facilities for the patients it would be the best.

**F2**; any other reason why…

**P4;** some times I see that what makes it hard is that I have failed to understand that is it really mosquitoes that are responsible for spread of Malaria because you can treat a child today and after three days the child is still sick and yet money is going and it becomes a hard for us and that’s why one day we were asking ourselves whether its mosquito only responsible for the spread of malaria.

**F2**; so what is the main problem there, is it money?

**P4**; yes, the main problem is money because even if you go to the hospital, still they will tell you to go and buy.

So the buying is the problem since we don’t have money.

**P3;** so the main problem are two, first the drug not being there and secondly the money to buy these drugs and yet the money is not there.

**P2;** like for the government health centers just like colleagues has said it, you will find that everything you have to buy and yet you don’t have money to them and there is sickness/ill health.

**P4;** you may go with money but you will find the price is high.

**F2;** ok, let go to our last question.

**F2; Do you think being a man or a woman would make a difference in you or your family accessing praziquantel or using praziquantel?**

**P2;** the answer is yes. The reason being as a man you have to plan for the welfare of the family and later you can plan for the medicines if it’s needed.

**P3;** he has almost answered it all because we blacks the responsibility of the family is on the man.That’s why I thank God for creating me a man because if you are to fall sick ,I just can imagine what you would do if you were not used with the responsibilities.

**P4;** the role and the tasks are all for the men finished.

**F2;** ok, the role and tasks are for the men.

**F1;** men are the one who plans…

**P4;** yes

**F2;** fend though the ladies come first (laughs…)

**F2;** so apart from that those were the few questions if you are not adding on the last question. I think that is all what we had and from my side I would like to say thank you for coming...

**P4;** may be before you say the last word…

**F2;** ok,

**P4;** will you bring the results or help the community?

**F2;**as we earlier said the purpose of the study ;number one the issue of gender to do with bilharzia exposure, prevention and two the use of praziquantel in the gender (the male and the female) and all the decisions will come back to help in the decisions making in the community the decision will come back to us.

**P4;** may be you add something to us.

**F2;** I will add like this this disease here is still with us, like in our community Pakwach, when we sample randomly you will find that five out of ten people are having Bilharzia that number is 50% so the government has brought so many strategies to reduce the burden of bilharzia but it’s not going down .According to the government we are supposed to clear or eradicate bilharzia by 2025 but we may not reach there why?

That is why this question is coming that is the role you play as a man or the role you play as a woman which is still hindering us from getting rid of this Bilharzia. You can see that if you cannot up a decision according to what you have said if don’t have the money as a man you cannot buy the drugs.it is a problem ,if this trend continues we may not reach there, so it’s a gap. This kind of research is going to help us reach there. Why it is that women are more exposed than men, which activities of women are there making them get more bilharzia than men.so we are trying to filter out this.so this can or research will help us get interventions to plan and get back to the community to help us get rid of bilharzia because this water is still with us.

**F2;** can I share something just small,

**All participants;** yes

**F2;** last week we did some studies were we picked two communities were one was kivujje and the other Nyakagei.in kivujje we sample/collected one hundred (104) of children eight twelve to fifteen both boys and girls, one hundred and two (102) were positive and only two were negative.

Then in Nyakagei we collected samples from one hundred and eight (108), we got 108 were positive and 0 were negative.am giving you results which is being shared nationally.

**F1;** this are results of last week. The strength of this disease is more than corona, we fear corona for nothing because this is what is going to kill us.

**F2;** thank you very much every one.
