# Supplementary material for: Gendered lives, gendered Vulnerabilities: An intersectional gender analysis of exposure to and treatment of schistosomiasis in Pakwach district, Uganda
Source: PLoS Negl Trop Dis. 2023 Nov 10;17(11):e0010639. doi: 10.1371/journal.pntd.0010639 (PMC10684070; doi:10.1371/journal.pntd.0010639)
Supplement: S1 Data — (ZIP) [file pntd.0010639.s001.zip › KII Schisto Interviews/KII Dr. Ajal Paul.docx]

***Study title:*** Gender intersectionality

and

Schistosomiasis in rural Uganda

***Interviewer:*** *Nakiranda Salama*

***Respondent:*** *Dr. Ajal Paul* ***Position/Designations:*** *Medical doctor, DHO*

***Proceedings;***

- *Interviewer welcomes Respondent*
- *Interviewer introduces herself*
- *Introduces the Project and Project Leads*
- *Funders*
- *Reminds Respondent of some crucial ethical considerations (Note: Respondent had signed the consent form)*

***Grand Tour Question:***

*How does gender intersect with other factors towards influencing preventive chemotherapy and WASH interventions in Pakwach?*

***Interviewer***: Can you please tell us about?

***Respondent:*** My names is Ajal Paul, I am a male, a medical doctor by training and a public health specialist. I am the District Health Officer in Pakwach District. I oversee the health facilities in Pakwach District. I oversee the health facilities in Pakwach District.

***Interviewer:*** *What is your* role in relation to Schistosomiasis control/prevention?

***Respondent:*** I am the overall coordinator of all project related to Schistosomiasis control and prevention or any other thing in the district. All the projects between the Ministry of Health and other partners are coordinated by me. I also appointed a focal person officer who coordinates the activities related to schistosomiasis on my behalf.

***Interviewer:*** What policies do you use for guidance in your overall work?

***Respondent:*** All of our work?

***Interviewer:*** Yes all your work as a medical doctor and DHO.

***Respondent:*** Of course we do everything in line with the Constitution of Uganda and being a local government, we operate using the Local Government Act which enables us to operate as a local government entity. In addition, we have many policies of ministry of health which we use. Currently, we have gone into the National Development Plan III (NDP III). We just completed National development plan 2 which captures all that we do in terms health services in the district. But there are many other policies and guidelines that we use. As you know, health is wide we have policies in relation to family planning, HIV Policy, non-communicable disease health document. We have many.

***Interviewer:*** Are policies that use specifically for schistosomiasis?

***Respondent:*** I can’t recall one off head at the moment but we have a number of guidelines on schistosomiasis control and prevention. Because schistosomiasis has been in this district for a number of decades.

***Interviewer:*** I hope we can contact you later for those.

***Respondent:*** *Sure*

***Interviewer:*** What are the key predisposing factors to schistosomiasis?

***Respondent:*** There are many. One is of course poor hygiene and sanitation in the community. We still have poor latrine coverage of less than 70% and like only 20% of our villages are open defecation free. Latrine coverage might be about 60% but not everyone in those homes with latrines still uses those latrines. We still have people defecating in the open and when it rains all this is washed back into the river where there are members of the community doing activities like fishing and fetching water. The water is therefore infested with schistosomiasis.

The river is a source of livelihood for the people and the community here have their livelihood in the river. Fishing is the main economic activity which is done in the river by the able bodied people. The main source of water to use in the homes is also the river. So we get the girls going to the river to collect water and the children also bathing from the river.

***Interviewer:*** How are men at a higher risk?

***Respondent:*** Men are ideally the bread winners and they must be able to earn and sustain the family. Like I said, one of the major activities is fishing and men are the ones that fish which makes them come into prolonged contact with the water of the river which exposes them on daily basis to schistosomiasis infection.

***Interviewer:*** *And women, what makes the risk higher for them?*

***Respondent:*** While as women are not so much involved in fishing but they also participate for example when the men deliver the fish from the river at the river shores or landing site, it is women who are there to receive the fish and process the fish and they do all this while exposed to the water. They actually do this while standing in the water. Therefore, young women and middle aged women definitely are at a higher risk. They also have to fetch water for domestic use and to get the cleaner water, they have to move in the water so their legs and feet are in the water for duration they use while collecting the water from the river. Then they also do a lot of washing in the water. During this they still stand in the water to do the entire washing of clothes and this exposed them to being infected with schistosomiasis.

***Interviewer:*** How are pregnant women vulnerable?

***Respondent:*** Pregnant women depending on the stage of their pregnancy they do the same activities as other women, they still have to fetch water and get engaged in other activities that they usually do before pregnancy.

***Interviewer:*** You have said the livelihoods in fact the lives of people in Pakwach depend on water, at this present time, how possible or realistic is it to prevent skin contact with high-risk schistosoma waters for each gender type? Give reasons for your answer

***Interviewer:*** How is possible for females?

***Respondent:*** It can be done through some engagement and talking to them and explaining the risks involved with the waters. I still believe that this water can be collected and brought out for use without entering the water and exposing themselves but the community has to first understand and appreciate the risks involved when they enter the water. If they don’t appreciate it, they won’t see the need. If they are made aware of and convinced I think they can stop that exposure.

***Interviewer:*** Hasn’t there been sensitization already? Haven’t they been made aware of the risk?

***Respondent:*** I have been in this district over 16 years and I think the emphasis has been on mass drug administration not so much on health education especially engaging these people where they do these activities from. People infested with schistosomiasis are not even aware until such a time when they are very sick.

***Interviewer:*** How can it be done with males?

The males also like I said while they are in the river doing the fishing, the exposure is not much but while they are preparing to enter the boots or when they delivering the fish, they spent a lot of time at the shores, the shallow water at the shores where the schistosoma is more prevalent. They stand there in the water which if they are made aware they can change.

***Interviewer:*** Anything different for the pregnant women?

***Respondent:*** It is still the same but I think we can put more emphasis on pregnant women and try to discourage them from going into the river.

***Interviewer:*** What are the nature of treatment seeking behavior with regard to Schistosomiasis?

***Respondent:*** Well, schistosomiasis as it the majority of the community members who are infested with schistosomiasis are not even aware that they have a problem so it is only those ones who are symptomatic, who have may be abdominal problems and other symptoms those ones do come to the health facilities. Well, depending on the level of the facility like where there is a laboratory the tests can be done and the medicine given but the majority are not even aware that they have schistosomiasis in their bodies until such time of mass drug administration and everyone is given this treatment.

***Interviewer:*** What gender issues affect treatment seeking behavior of schisto patients?

***Respondent:*** In my opinion**,** women have a better health seeking behavior for schistosomiasis and generally for many of other health problems. When you go to any of our facilities you find that the queue is filled with women. I think this is because women are more conscious of their health than the men and they also have to bring children to the health facility.

**Men,** by the time they come for treatment, it is already late. I think this is because women are more conscious of their health than the men. The men feel they are strong and they have work to do. They continue doing their work and by the time they are coming the disease may has already progressed and forced them to come but we have seen women and their children they are more. They come more quickly to the facilities than the men, generally.

***Interviewer:*** How does being female or male gender or others (that’s is man; woman, mother/ father, pregnant mothers) influence behavior change and praziquantel uptake towards better control of schistosomiasis in your district.

***Respondent:*** In my opinion or from what I have seen, women are more likely to take the PQZ tablets than he men. Men have a belief that before you take PQZ tablet you have to prepare before you take it and when they are given they put it aside and postpone taking it until may be when they have the right time then they will take it. And sometime they end up not taking it. Sometime find a man with schistosomiasis and then you ask what was the last time you took PQZ and the person will tell that I have the tablet in the house but they have not even taken it. For women once they have come for any treatment and they are given tablets, they are more likely to take.

***Interviewer:*** Can you please tell us about your experience in implementing interventions to control schistosomiasis in your community?

***Respondent:*** Well, my experience has been more of supervision, and they have not actively participated in the actual implementation but I have been involved in planning and supervising and at the end of it all we share report of interventions done. Am also involved in coordinating partners who are involved in schistosomiasis control in the district on implementation and I have also participated in some studies related to schistosomiasis as student and also as a health manager.

***Interviewer:*** Ok then, Praziquantel mass drug administration is one of the key interventions for treatment, control and prevention of schistosomiasis. Please comment on its access and how it has helped as far as prevention and treatment are concerned.

***Respondent***: My assessment as a health professional who has been treating all stages of the schistosomiasis disease and working in the community which has one of the highest prevalence of schistosomiasis in the world is that praziquantel mass drug administration has not achieved much. I have been in this place for the last 16 years. I have seen year in year out the mass drug administration. At best the mass drug administration is given twice a year but of recent it has been used to once a year and I think that is because of resources. Twice every year the whole community would get PZQ and then eventually it reduced to once a year I think because of the limited resources mainly. Then of recent it reduced to only school going children. But still even if they take the medication, the next year they would still be in contact the water. I have not even noticed any improvement.

***Interviewer:*** So, what would you do better (generally) to improve the situation?

***Respondent***: Of course praziquantel is a proven drug and it kills the schistosomiasis in the body. That one there is no doubt. Every time it is given the load of the schistosoma in the community is reduced but like I said if these drug is administered once a year that means in the next 11 months of the year, the community is still exposed and the disease has time to progress in their bodies until the next mass drug administration, even at 100% access still there is a period where these people are vulnerable.

So I would think that there should be another approach on improving on the sanitation of the people and improving access to clean water away from the river. This cam ensure that throughout the year if the disease is infested in someone’s body. All members getting this would be better and also of focus together with improving the sanitation.

If I say something about improving access, everyone here who is above one year is exposed. Therefore it should the whole community getting the drug around the same time and if possible the minimum it could be at least twice a year. So twice a year all members of the community of Pakwach getting this praziquantel would improve it access and may be afford some protection for the community, of course these should be done while also strengthening sanitation and access to clean and safe water*.*

***Interviewer:*** What would you do better focusing on different gender (men vs. women vs. pregnant women, fathers, mothers, aunties, uncles, grandfathers, grandmothers, girls or boys)

(At work/ by occupation/ economy, in the family, in the health facility, or in political administration) help improve access to and utilization of PZQ?

**Family and workplaces**

***Respondent***: Am convinced that in partnership with other partners of the local government, it is possible to use this same water of the river, if treated water and made it safe for the community. Of Pakwach. It is not a very big thing the water is near them it is just a matter of treating the water now this would help both the parents at home and the men fish and all people at landing sites because all these use this water.

**Community and Local Government**

***Respondent***: Then two, the local government together with the partners can work together to improve the sanitation in the community and this we are trying to do by improving the latrine coverage and latrine use and we can also achieve defecation free status where no one deposits their wastes out in the open once this is done it will break the cycle of schistosomiasis spread and transmission. Once this is done, it will ensure that all these categories both men and women are catered for. It will break the cycle .This will make the life of the women better for women it may cut them off from getting in contact with high risk water.

***Interviewer:*** What changes in gender (roles, responsibilities, behaviors, expectations, or individual characteristics linked to a perceived sex identity) do you think can improve preventive chemotherapy or WASH in Pakwach?

***Respondent***: I can’t put my fingers easily on what changes can be made. For example for the women, their biggest access is the river. Probably if this role of providing water was shared with the men or boys but still the exposure would change to the boys.

The role of taking children to the health facilities is basically for the women so if this role was shared with the men probably it would improve on access to health care. The women have many responsibilities. Fetching water, preparing the food. Taking the children to health care facilities may be delayed but if they are sharing this responsibility with the men, access to chemotherapy, praziquantel would be improved. When the woman is doing other things at least the man should be able to ensure that those who need chemotherapy get it.

There should be something done to improve the wash status in the community.

Improving the safe water, because water is plenty in the water bodies. All the communities in Pakwach are called people of Jonam. Jonam means people of the water literally leave alone the river. It would not be a very tricky once this is done**.**

Then lastly is on economic empowerment of the community, so empowering the community to do other activities like farming, tourism, trading. Other aspects that put food on their tables and may sustain their livelihood to be able to reduce on the dependence on the river**.**

***Interviewer:*** Are there any comments/recommendations/suggestions you would like to make?

***Respondent:*** No

***Interviewer:*** *Thank you so much for your time and agreeing to be part of this.*
