# Supplementary material for: Gendered lives, gendered Vulnerabilities: An intersectional gender analysis of exposure to and treatment of schistosomiasis in Pakwach district, Uganda
Source: PLoS Negl Trop Dis. 2023 Nov 10;17(11):e0010639. doi: 10.1371/journal.pntd.0010639 (PMC10684070; doi:10.1371/journal.pntd.0010639)
Supplement: S1 Data — (ZIP) [file pntd.0010639.s001.zip › KII Schisto Interviews/KII Dr. Oryema JB.docx]

***Study title:*** Gender intersectionality

and

Schistosomiasis in rural Uganda

| ***Interviewer:*** *Assoc. Prof. Sarah Ssali*  ***Respondent:*** *Dr.* Oryema John Bosco ***Position/Designation:*** *Incharge of Pakwach HC IV*  ***Proceedings;***   - *Interviewer welcomes the respondent to the interview* - *Interviewer introduces herself* - *Introduces the Project and Project Leads* - *Introduces Funders* - *Reminds Respondent of some crucial ethical considerations (Note: Respondent had signed the consent form)*   ***Grand Tour Question:***  *How does gender intersect with other factors towards influencing preventive chemotherapy and WASH interventions in Pakwach?*  ***Interviewer:*** Can you please tell us about yourself?  ***Respondent:*** My name is Oryema John Bosco. I am a medical doctor and the Incharge of Pakwach health centre IV. I have been the incharge for one year. |
| --- |
| ***Interviewer:*** What are the predisposing factors to schistosomiasis?  ***Respondent:*** The gender domestic activities expose women to schistosomiasis. Females are more exposed compared to the males because of domestic activities like fetching water and actually some of them do fishing but most factors that predisposes them is fetching water from the lake.  ***Interviewer:*** How does fetching water affect expose them to infection  They go to fetch water and step the in water body which is not safe water and that is how it affects them.  ***Interviewer:*** Which category of people is more predisposed? Are they the poor ones, the ones closest to the lake, things like that?  ***Respondent:*** Yes, does who are close to the water source and those with poor economic status compared to those in average status are more affected. They can’t access safe water like the tap water which requires payment.  ***Interviewer:*** What are the nature of treatment seeking behavior with regard to Schistosomiasis  ***Respondent:*** The treatment seeking behavior is poor because most of them are always coming with complications like vomiting blood. So they delay until they get severe complications.  ***Interviewer:*** What gender issues affect treatment seeking behavior of schisto patients?  ***Respondent:* I**n early stages early, we receive more of females, males come late than the females.  ***Interviewer:*** So how do capture the prevalence among males?  ***Respondent:*** For the prevalence of males we capture in terms of complains sometimes it is community leaders who report the cases in the community. Some people do not report even when they have complication especially men.  Men generally have poor treatment seeking behaviours. They do not take priority of their health. Also men’s smoking and taking alcohol make them present complications late. It makes them have more complications that female.  For women’s late complain is because they are sometimes taken up by domestic work and they have no time for coming to the facility. They are not empowered to access facilities, they lack financial support and transport facilitation. Sometimes they are not even aware of the symptoms.  ***Interviewer:*** Are there any activities you have done to encourage behavior change?  ***Respondent:*** We normally discuss with health centers no behavior change activities in the community.  ***Interviewer:*** Does Schistosomiasis re-occur or you can’t get infected again you have ever been infected?  ***Respondent:*** Schisto recurs, they get new infections, and there is no clear data of those re-infected. Reasons for reinfection include, exposure to the water in domestic activities.  ***Interviewer:*** How does being female or male gender or others (that’s is man; woman, mother/ father, pregnant mothers) influence behavior change and praziquantel uptake towards better control of schistosomiasis in your district.  ***Respondent:*** Females always comply better with the information given at the health facility than the males.  Treatment! The females always comply with treatment compared to the male. Like I said men have social habits like smoking and drinking which blocks their compliance.  ***Interviewer:*** What else affects the men?  ***Respondent:*** Sometimes when you give them appointment they don’t respond. Most of them are in a low level social economic status and they depend mostly on fishing. |
| ***Interviewer:*** Mass drug administration is one of the key interventions for treatment, control and prevention of schistosomiasis.  How successful has it been?  ***Respondent:*** Right now I don’t have the evidence based data.  But the challenge is that most patients complain of abdominal complains after swallowing.  Some females have complained of pregnancy termination.  Sometimes they can’t take it while preparing to conceive.  Some complain of the smell.  ***Interviewer:*** Do both genders complain of these?  ***Respondent:*** Yes but If they eat before swallowing then no complications.  ***Interviewer:*** What have you done to influence behavior change?  ***Respondent:*** The community education, dialogues and intervention however we have not been able to access all the communities. Most of the dialogues are done at the health facilities. |
| ***Interviewer:*** At this present time, how possible or realistic is it to prevent skin contact with high-risk schistosoma waters for each gender type? Give reasons for your answer  ***Respondent:***  It may be very hard because people cannot stop getting into contact with the activities that support them. I suggest a progress approach which we should come up with together with them. |
| ***Interviewer:*** What changes in gender (roles, responsibilities, behaviors, expectations, or individual characteristics linked to a perceived sex identity) do you think can improve preventive chemotherapy or WASH in Pakwach?  ***Respondent:*** More boreholes within the community*.* Can’t afford monthly water bills for water, more latrines for the population. Dig more to see how it affects the gender. |
| ***Interviewer:*** Do you have any comments or suggestions?  ***Respondent:*** No.  ***Interviewer:*** Thank you very much |
