# Supplementary material for: Gendered lives, gendered Vulnerabilities: An intersectional gender analysis of exposure to and treatment of schistosomiasis in Pakwach district, Uganda
Source: PLoS Negl Trop Dis. 2023 Nov 10;17(11):e0010639. doi: 10.1371/journal.pntd.0010639 (PMC10684070; doi:10.1371/journal.pntd.0010639)
Supplement: S1 Data — (ZIP) [file pntd.0010639.s001.zip › KII Schisto Interviews/KII Mr. Odongo Tom.docx]

***Study title:*** Gender intersectionality

and

Schistosomiasis in rural Uganda

***Interviewer:*** *Nakiranda Salama*

***Respondent:*** *Mr. Odongo Tom.* ***Position/Designation:*** Comprehensive nurse, Incharge (Abolo health Centre II)

***Proceedings;***

- *Interviewer welcomes Respondent*
- *Interviewer introduces herself*
- *Introduces the Project and Project Leads*
- *Introduces Funders*
- *Reminds Respondent of some crucial ethical considerations (Note: The Respondent had signed the consent form)*

***Grand Tour Question:***

*How does gender intersect with other factors towards influencing preventive chemotherapy and WASH interventions in Pakwach?*

**Interviewer:** Can you please tell us about yourself

***Respondent:*** My name is Odongo Tom. I am I male and am 28 years old. I am an enrolled comprehensive nurse In charge of Abolo health Centre II in Pakwach District Local government. I am the in charge nurse. Health facility in charge and also the in charge of surveillance for these topological cases like schistosomiasis.

**Interviewer:** What is your role in relation to Schistosomiasis control/prevention?

***Respondent:*** Being the in charge, my role is whenever we get these cases of schistosomiasis ensure they are well diagnosed. We make sure these people access the treatment more especially paraziquantile.

**Interviewer:** What policies guide his/her work?

***Respondent***: We have our professional code of conduct which guides us to give quality work. Even health guidelines which are provided by ministry of health and also the constitution of Uganda also guides us. Because we have articles that guide the health workers or civil servants. Some part of it also guides us to provide quality services to our patients and community.

**Interviewer:** Are there policies or guidelines that guide your work in relation to Schisto?

***Respondent***: We normally consider the general health policies. There is no specific policy that guides to schistosomiasis treatment. May be in terms of clinical guidelines, but mostly we can follow the clinical guidelines that guide us to give services to schistoma patients.

***Interviewer:*** What are the key predisposing factors to schistosomiasis? What are those that make the general population vulnerable?

***Respondent:*** One is the economic activity that the community is doing is fishing. They are around the lake and the river. When they are entering the lake, they are not protected more so others are having wounds, cuts on their bodies, the schistosoma can easily access and enter their bodies. Two, we have these women more especially they prefer that clean water like from the borehole is made for drinking and using this lake for washing clothes and cooking and even bathing at home. You find somebody leaving the borehole there or they fetch borehole water with two jerrycans. The line of people at the borehole is also always long. So when they fetch water from the water bodies, they are exposed to schistosomiasis and also children who like swimming. Right now the water level has risen and it has killed very many people. The waters become stagnant near the homesteads and you find children playing in that water.

***Interviewer:*** What factors put men at a higher risk?

***Respondent:*** They do the fishing and it keeps them in the water. There are some activities, we have these brokers they do transport activities on boats that carry people. They carry people to the boats. Men are the ones that participate as brokers carrying the people from dry area to the boats in the water bodies.

***Interviewer:*** What about women, what predisposes them apart from fishing water which you already talked about?

***Respondent:*** For us here, apart from fishing these people also participate in collecting snails, we call them osongolo. Women also participate in that thing. You find women in the morning hours like at around 5 to 6 am they go and collect these snails. They also participate in it and it can make them to contract the diseases while they are in the water collecting those snails for chicken feeds**.**

**Interviewer:** Is it still the same case for pregnant women?

***Respondent:*** The pregnant women can also get this disease because they also participate in these activities. One is that when it comes to this praziquantel they cannot take it because it can terminate the pregnancy. Previously there was research done and there was a policy where people should get this praziquantel every person whether you have test or not but women who are pregnant were not allowed to take.

**Interviewer:** So most of the people’s activities in Pakwach are done in the water. At this present time, how possible or realistic is it to prevent skin contact with high-risk schistosoma waters for each gender type? Give reasons for your answer.

**For women;**

***Respondent***: I don’t think it can really work. I can say by health educating them. For example if you know you have wounds don’t enter in water because you will get in contact with schistosomiasis. In fact you should stay away because there is no another way like protective gears or overalls they can put on they enter water they would be safe. Even the same thing applies to men and the pregnant women. So the only best way is to health educate them and

Secondly routine distribution of praziquantel so that everyone gets and the whole community is treated.

***Interviewer:*** What is the nature of treatment seeking behavior with regard to Schistosomiasis?

***Respondent***: There is one thing we learnt from these patients some fear to swallow the medication, they talk about the side effect that the medicine has high side effects. Whenever they swallow sometimes they vomit. At times when you get to direct observation therapy most especially for children, they swallow it on empty stomachs like these kids from school. So you find for the kids may swallow it on empty stomach even when it disturbs them. But others you may give them and they say they will swallow later but they may not even be in position to swallow. And secondly the availability of this medicine, it may not be easily accessible you find right now I don’t when they last distributed this medicine and am sure there are already people who need it if they can make it routinely like this vaccination thing it would work better for our communities around and it would reduce the high rate of the complications which people are getting from schistosomiasis like voting of blood and others.

***Interviewer:*** What gender issues affect treatment seeking behavior of schisto patients?

***Respondent***: The gender, men here are meant to be fish mongers. They go and do fishing in the lake. So when you take this praziquantel home you may not find them. Moreover sometimes they go and stay at the lake for two to three days. They takes their utensils there to cook form there. It is hard to give them praziquantel. Sometimes they end up missing out because of this activity and since they are men they feel strong and do not usually come to the health facility.

Even this alcohol behaviours, you find someone who is addicted to taking alcohol will not mind taking this medicine. He will not have time for this medicine but time for alcohol. And we have seen even with other treatment they do not comply well.

**Interviewer:** What about women? What affects their treatment seeking behavior?

***Respondent***: Women carry pregnancies so they miss out sometimes. Moreover when the mass drug administration is done when the woman is pregnant, she may not get another chance. She may take after delivery but also sometimes it is not safe for a woman to swallow some of these medicines while breast feeding.

And then the activities of women, they have a lot of work, domestic work and they can easily forget to swallow the medicine.

**Interviewer:** How does being female or male gender or others (that’s is man; woman, mother/ father, pregnant mothers) influence behavior change and praziquantel uptake towards better control of schistosomiasis in your district.

***Respondent***: I think we need joined action to attain the attention of these people. We need to involve all the stakeholders, we have to come together. Like measles and cholera campaigns we managed to make people come together. And in these neglected tropical disease it is also important that we do that.

***Interviewer:*** So if people come together what would be the influence of the man and that of the woman?

***Respondent***: People need health information first and it matters who is giving this information. Our communities here they are usually. You know they here better when somebody is talking like VHTs. Sometimes we need to put health assistants on ground or these clinical teams. They should give this information first. Like for me, I always tell them like they assume vomiting blood at late stage when they have bilharzia it is still superstition like in our area here they say they have problems in their family, or they are being bewitched or they failed to pay dowry for their grandmother. So they me am ever telling them that is bilharzia in a late stage and they usually consider it. Right now even go to the hospital for control. So those are the things. So whoever gives this information, it matters a lot.

**Interviewer:** Does it matter if man/mother or woman/father is the one giving this information?

***Respondent***: If the father or mother gets the right information at the right time from the right person, they can pass this information to the people at home. They can change the behavior from home.

Also if we get a peer client. Someone who has survived the bilharzia, they can also pass this information to the people which can help them better.

***Interviewer:*** Can you please tell us about your experience in implementing interventions to control schistosomiasis in your community?

***Respondent***: One is that, others accept their condition others do not. Some even come openly to the health centers to access these services. What limits us is the medicine and the services sometimes are not up to standard so.

Two, the cultural beliefs about this disease are still dominant. People are not fully informed others still belief that if their grandparents used to take this water long time ago but they were not getting this disease why is it that it is this time that they are getting it.

We usually tell them that those people used to die of this disease but they were not noticing and others are now trying to understand.

There is also challenge people that still have challenge in accessing clean water for example in Panyamur. Even where there are boreholes, they are few and you find a very long line so some people just leave and fetch water from the lake where they won’t face such challenges. Also this national water sewerage cooperation is here running but people cannot put up with those bills therefore they have to keep fetching water from the lake. Now the flooding level is high the toilets that were there have broken down and people are now, they don’t have toilets which means they are using the same lake for defecation.

Also men’s behavior. Men when they are going for fishing there is no latrines at the lake so they just use the lake in other ways they are infecting themselves there.

Sometimes we organize quarterly meeting with them to ensure they learn and they share their experiences with us. Those are the things they learn from them and the community here.

***Interviewer:*** Praziquantel mass drug administration is one of the key interventions for treatment, control and prevention of schistosomiasis. Can you comment on its access and how it has performed in regard to prevention and cure of schistosomiasis?

***Respondent***: Well, I could say it has done a good job for some but the accessibility is not that to standard as I was saying this drug, when it reaches on ground we highlight the clinical things. These are nurses, clinical officers and medical officers. You find this medicines when it come s it is pushed to the hands of clinical assistants or health officers. The health teams are the ones that distribute so you find this one alone. You can’t know whether they have done it well or not on the ground there but we believe when we get these people and medicines are there, we give them immediately to these people we know it is DOT direct Observation Therapy they should get it. So from there I still see the access is not okay it is poor we should get another channel of how these people can access the medicine.

***Interviewer:*** Has it done some level of controlling?

***Respondent***: The rate of the disease is coming down. But my worry is during that time when the medicine is not there I think the disease is raising. If there is a way the donors or the ministry of health can make this medicine to be routine it should be accessible. It should be there all the time because when it is the clinic it is expensive for these people to access. But now days the cases are not as before.

***Interviewer***: What would you do better (generally)?

***Respondent***: We need the medicines to be distributed in all facilities and health workers should access it all. It should not be just specific people, the teams have to be clear. Secondly, we need mentors on the ground for the health teams so that they can do their work better. Health education, people only get some health information when they get to the facility we need to take the information to them. It would be better if the community is well informed about this schistosomiasis. Also the health teams need to be well trained and given guidelines. I, am basing on guidelines should be produced by government and if there are some new updates about it we need to access it.

***Interviewer:*** What would you do better focusing on different gender (men vs. women vs. pregnant women, fathers, mothers, aunties, uncles, grandfathers, grandmothers, girls or boys)

(At work/ by occupation/ economy, in the family, in the health facility, or in political administration) help improve access to and utilization of PZQ?

***Respondent:*** In relation to men, women and children activities. Those are cultural based things.

Still for women we need to talk them because as I had said what is making them not to take the medicine and not to go to the health facility is that they have too much work at home We need to give them DOT they swallow then they go and do their work and other things or we tend to advise them. And also to be informed that if they have wounds on their bodies they should not enter the river because they might contract the disease.

For men, it is the same thing we also need to talk to them when they have wounds they should first wait before they enter into the water for their economic activities like fishing. We need to sensitize them.

For the children, we have a role to play because whenever I pass on the road, you find them collecting this dirty water swimming. We need to be chasing them from such, everyone in the community because they are doing wrong things. We parents we need to talk to them.

We need to involve everyone, at the health facility. Even the political will has to be there so that people will get positive attitude to praziquantel up take. When we were distributing the cholera vaccine it was better because the person would take the vaccine when we are seeing so that is one of the area where we see can contribute to the uptake of this praziquantel.

***Interviewer:*** What changes in gender (roles, responsibilities, behaviors, expectations, or individual characteristics linked to a perceived sex identity) do you think can improve preventive chemotherapy or WASH in Pakwach?

***Respondent:*** One is access to clean water for the women because they are the ones fetching clean water from the lake. Government needs to provide access to clean water and make it affordable to the community members. Two, we still need to health educate them. Many people learn from things like talk shows on radios but they are very rare.

On the side on environmental health, people need to have toilets. They nay. Mostly that area if we start from that angel we can improve from there. Clean water has improved some communities already who have access to clean water.

For men, as I have already said it is sensitization so that they can protect themselves while they go to fish, like not entering the water when they have wounds.

***Interviewer:*** Do you have any comments/recommendations/suggestions?

***Respondent:*** No, I think that’s all I had.

***Interviewer:*** Thank you very much sir.
