# Supplementary material for: Gendered lives, gendered Vulnerabilities: An intersectional gender analysis of exposure to and treatment of schistosomiasis in Pakwach district, Uganda
Source: PLoS Negl Trop Dis. 2023 Nov 10;17(11):e0010639. doi: 10.1371/journal.pntd.0010639 (PMC10684070; doi:10.1371/journal.pntd.0010639)
Supplement: S1 Data — (ZIP) [file pntd.0010639.s001.zip › KII Schisto Interviews/KII Mr. Odongo Yoweli.docx]

***Study title:*** Gender intersectionality

and

Schistosomiasis in rural Uganda

| ***Interviewer:*** *Assoc. Prof. Sarah Ssali*  ***Respondent:*** Odongo Yowel ***Position/Designation:*** Health Inspector of HCIV, Pakwach  ***Proceedings;***   - *Interviewer welcomes the respondent to the interview* - *Interviewer introduces herself* - *Introduces the Project and Project Leads* - *Introduces Funders* - *Reminds Respondent of some crucial ethical considerations (Note: Respondent had signed the consent form)*   ***Grand Tour Question:***  *How does gender intersect with other factors towards influencing preventive chemotherapy and WASH interventions in Pakwach?* |
| --- |
| ***Interviewer:*** Can you please tell us about yourself before we start?  ***Respondent:*** My name is Odongo Yowel. I am 38 years and I am a male. I am the Health Inspector of Pakwach Health Centre IV (HCIV) in Pakwach district. I have worked at HCIV for 2 years. I am a native of Pakwach district.  ***Interviewer:*** What does your work involve?  ***Respondent:*** My work involves promoting health and preventing diseases. This includes home visits, school inspections, school health and advocacy for sanitation (promotion), community health education, promotion of clean and safe water in the community and community sensitization at large to promote health.  ***Interviewer:*** So, that is very interesting to know, when did you graduate?  ***Respondent:*** In 2018, that is I graduate with my current qualification diploma in Environmental health service.  ***Interviewer:*** What are the key predisposing factors to schistosomiasis?  ***Respondent:*** You see our community has river Nile. People are always in the water women, children and men. They are exposed to the water 24 hours. Some go there to swim and bath while others to fish. There is also stagnant waters in the community where people go farming. People farm around. Then children go to catch fish, swim in that water other than the river. So those are the factors that expose them.  ***Interviewer:*** So when we come to gender, what makes men vulnerable?  ***Respondent:*** Men become vulnerable in the sense that fishing activities are always done by men who are ever frequenting in the water. Some are always there in the water day and night. This also involves boys. They begin to learn how to fish when still young.  ***Interviewer:*** At what age, do they begin entering the water to fish?  ***Respondent:*** Some start at a very young age depending on village and household they come from. Some begin as early as 10 years. So Poor households see it as the boy trying to save the family survival. They can even send a young boy from 10 years to go fishing.  ***Interviewer:*** What about the women, what renders them vulnerable?  ***Respondent:*** The vulnerability of woman depends on which home she comes from. Some homes cannot afford safe water so women from those households have to go and fetch water from the lake and also wash clothes which exposes them. Some households can only afford that water for drinking and they can’t use it for domestic work. Therefore there is relationship there between the household a woman is coming from and the vulnerability to schistosomiasis.  ***Interviewer:*** Is there any extra vulnerability for pregnant women or it’s the same with these other women?  ***Respondent:*** Some of them when are pregnant prefer water from river or swamp other than tap water, leaving chlorinated water. They feel that when they take tap water they get abnormal feeling like nausea, etc. Some might even send child to fetch water for them from the lake. But after delivery, they go back to normal.  ***Interviewer:*** Is that only for the poor women?  ***Respondent:*** No, it cuts across***.***  ***Interviewer:*** And for children apart from the boys, is there anything?  ***Respondent:*** The girl child are exposed depending on family she coming from. If it is family that fetches water from the lake she will also have to fetch water from the lake. Sometimes she is living with an old grandma, she will permanently go to fetch water in river or swamp. Also if family is poor, she will begin going to fetch water, they will say, this one can now carry a 10 litre Jerrycan.  ***Interviewer:*** At this present time, how possible or realistic is it to prevent skin contact with high-risk schistosoma waters for each gender type? Give reasons for your answer  ***Respondent:*** That possibility for me, it is not actually possible as per now because we can’t avoid living with poor households in this community. Much as we advocate for safe water but not everyone can afford it. Also we can’t afford the men not going fishing and we can’t afford telling them that when they go for fishing they should not enter the water. So that possibility, I don’t see it at the moment.  ***Interviewer:*** So you don’t see it whether for men, women or children as long as households are poor.  ***Respondent:*** Exactly.  ***Interviewer:*** Ok the next question would be, what is the nature of treatment seeking behavior with regard to Schistosomiasis?  ***Respondent:*** Treatment seeking behavior is Very poor in our community. People only come to the facility when they are seriously ill. Coming early is poor for all genders and ages.  ***Interviewer:*** How about locality, would the urban people treat better than the rural ones?  ***Respondent:*** When it comes to locality that one I support because the urban people in catchment area of the facility. They also here from the radio and when they get the information they come earlier than those in rural areas where someone is not getting any information or seeing any health centre..  ***Interviewer:*** Why do you think that the treatment seeking behavior is poor for women?  ***Respondent:*** Most of our women not empowered. They think there are there to serve others including men. That mindset and thinking of coming to the health centre on their own is not taken seriously by themselves. The problem is that they are not empowered, they are just buried in households concentrating on household work. Even when the health message reaches them they don’t care. Mindset and level of education matters. Those who are somehow educated and empowered seek health services. Most of them are just traditional African women who think since they got married to the man, the man is the one who is big for her she is just there to do domestic work. Even when she is sick you find her carrying basket and go to the garden instead of going to treatment. They value going to the garden better than going to treatment.  ***Interviewer:*** How about when children are sick?  ***Respondent:*** Even when children is sick they don’t act in time. They tend to ignore until the situation becomes worse. You find a child has been brought after 24 hour of a fever. They tend to buy medications such as Panadol until when it gets worse and when they reach there they become enemies with the health workers since they demand for services as soon as possible not knowing tht they are the ones who delayed.  ***Interviewer:*** We have seen what delays women. So what delays men?  ***Respondent:*** Taking children to the health centre is seen as women’s work. That is what men assume. For their own, they believe that at the health facility the attitude of health workers is not good. That they are even sent to buy medicines or that they won’t be handled the way they expect. So that mentality is there. Even when we are having community dialogues they the same things. Some women deliver from home because they say the attitude of health workers is not good.  ***Interviewer:*** What do they want health workers to do for them?  ***Respondent:*** Remember when I explained the issue of taking the child late and wanting quick services, You understand that. Attitude of community towards health workers is negative. Men expect some nice interaction when they go to the health facility but you no people are different. Even getting them to buy medicines is blamed on health workers, who they accuse of stealing medicines from the facilities. So that has brought every serious gap between health workers and the community. It is men who take it very seriously than the women. Men also complain of the delays to receive treatment when they go to the health facility.  ***Interviewer:*** So they want to be treated quickly, they don’t want to wait?  ***Respondent:*** Exactly  ***Interviewer:*** You have told us that children may delay because of parents’ attitude and commitment to work, is there any other reason?  ***Respondent:*** Yes, someone might say when if I take my child there, I don’t have money.  ***Interviewer:*** But aren’t these government facilities with free services.  ***Respondent:*** Yes, we don’t ask for money from them but the drugs are never enough and therefore we ask them to buy some.  ***Interviewer:*** How does being of female or male gender or others (that’s is man; woman, mother/ father, pregnant mothers) influence behavior change and praziquantel uptake towards better control of schistosomiasis in your district.  ***Behaviour change****:*  ***Respondent:*** Gender greatly affects Behaviour change, women and men respond differently. The response of women is better than that of men. Women are easily found at home when there is mass drug administration than men. They are the ones to take care of the home. So drug distribution finds them at home.  Men are not always at home, they are ever in the drinking joints or in the river fishing and doing other things. This also affects the male children. Boys have a responsibility to provide fish for household use. So during the distribution you ladies at home but not men.  During uptake of the drug women are also better. The men even when they pick the drug, they careless to take it up. Those who take alcohol think it can cure everything. Women even care to bring their children during mass drug administration. Most of the children brought by women. Men may come alone and you can whether they have others they say that their mother will bring them. Those are the kind of things.  ***Interviewer:*** How can we use gender to improve behavior change?  ***Respondent:*** To me, I would suggest that we can do the assessment, compare between a man and a woman, who has some basic knowledge and who can pick the message very well and try to speak to members in the household.  ***Interviewer:*** I mean you people working on the ground, how does the understanding of gender help you improve behaviour change?  ***Respondent:*** Like we had the issue of typhoid in one of the communities, it was actually very serious but when we realized the majority were women we convinced with leaders and targeted convening meeting with women. We then realized that most of the roles are left for women. Tried to advocate how to share gender responsibilities between men and women. It was very difficult to for women to believe that some activities could be done by men equally. We later brought in men and they were saying that some of the things are for women by God’s design. We also tried convincing them but it wasn’t easy. Therefore women are vulnerable and it’s because of lack of empowerment and education.  ***Interviewer:*** For you as a health inspector you appreciate the gender issues, you have seen them and yet we still need behaviour change, how can you use behaviour change to improve use and access to praziquantel?  ***Respondent:*** I think that one may be right away during mobilization we should involve the mobilisers to be of both sexes. In some villages we have VHTs who are all one sex while other have both sex. Some community members prefer to be addressed by a VHT of the same gender others of a different gender. We would propose because we also compare where you have a female VHT more women tend to turn up and the same on the side of men. So we should put male and female VHT to give them choice of where to go or pick medication from.  ***Interviewer:*** You said that you have done this since 2018, how has your experience been like?  What do you see as working well and what has not in controlling bilharzia?  **Good experiences:**  ***Respondent:*** The success has been the mobilization has been perfect on the side of distributors and administration of drugs people are eager and they turn up for the drug because people are informed that there is bilharzia from the river.  **Bad experience**  ***Respondent:*** The challenge has been the inadequacy of the drug, I don’t remember when we ever had enough drugs. Then the programme schedule the programme begins late and by the time they finish they want a report so distribution is done on pressure but majority of the people turn up except a few who dodge. The also the side effects for example on the school children. If they take without eating they get stomach pain. The medication has to be taken after meals so some children dodge claiming they have not eaten. No team moves around to check on reactions and effects, to give the community confidence. You get to know about reactions and effects after some days.  ***Interviewer:*** Are there are specific gender experiences whether good or bad?  ***Respondent:*** Some pregnant women who may wish to take the drug but by standard the drug is not allowed to pregnant women. Also when one is very sick you do not treat. But the pregnant woman may have high interest of taking the drug and some say give me I will take after I deliver.  ***Interviewer:*** So when it comes to adherence, do the men adhere very well?  ***Respondent:*** Those who come take but there are those drunkards who do not surface and you just have to look for them other who are responsible come.  ***Interviewer:*** How do you look for these drunkards who have refused to come?  ***Respondent:*** Drugs are distributed by VHTs. They plan to capture the man in the morning when he has not gone for drinking. But there are some stubborn ones who refuse and go away.  ***Interviewer:*** When we come to Praziquantel use what are the access issues you have come across, you have told us that there is Inadequate supply is there anything else that affects supply?  ***Respondent:*** Some villages are large with a very big population yet there are only two VHTs to serve the village and the drugs are distributed at particular time schedule, all this affects access. The previous distribution was done during corona time and they had to move from home to home. Distributors are few but some village are wide with high populations. When we advocate for more distributors, there is also the financial constraint.  ***Interviewer:*** Who appoints the drug distributors?  ***Respondent:*** They come from the village. The village members choose their volunteers, somebody who has the qualifications and is willing.  ***Interviewer:*** Why do they choose few?  ***Respondent:*** Ministry of Health budgets for two per village whether the village is big or small. This becomes a challenge for the distribution time.  ***Interviewer:*** How does gender play into that?  ***Respondent:*** The gender issue here is that with the women being responsible to take children to distribution point. The man expects the woman to take all the children. Assuming she is pregnant or sick, she may not be able to take the children.  When it comes to prevention and treatment using praziquantel what are the issues there?  ***Respondent:*** I talked about treatment. For men whoever comes can take the drug and those who don’t come don’t take. Women do not come when pregnant because the drug can terminate the pregnancy.  Then about prevention, refer to my explanation at the beginning. Prevention would mean that we avoid contact with contaminated water, take medication and improve on sanitation in our environment. This interrupts the life cycle of the schisto. The challenge is that because of poverty, poor women can’t afford safe and clean water for drinking and fishing therefore they cannot avoid contact with the water. So the poverty is the major issue there. It affects men, women and children especially boys.  ***Interviewer:*** Do you ever use praziquantel for prevention?  ***Respondent:*** No, by standard we should give it twice a year. We don’t have it for prevention in the community.  ***Interviewer:*** So all these people you are giving in the community and at schools it is for what? Do you distribute to everyone or you first check to see who is sick?  ***Respondent:*** Yes, everyone is supposed to get.  ***Interviewer:*** Is it for prevention?  ***Respondent:*** Of course we are preventing.  ***Interviewer:*** How many times do you distribute?  ***Respondent:*** Twice a year. I said the reason why we do not for the ministry of health guidelines od distributing twice a year, is because it has never been there all the times.  ***Interviewer:*** Since 2018, have you ever seen it being distributed?  ***Respondent:*** Before 2018, I was working in another sub- county called Wadilla, by the time we used to give twice a year in every community and everyone above 5 years. Since 2018, I have never seen it being given.  ***Interviewer:*** You gave us gender issues concerning access, prevention and treatment of bilharzia. The question now is that what would you do better?  ***Respondent:*** At the moment want we are doing differently is sanitation promotion and prevention. That everybody should have a latrine and there should be no open defecation. We are also advocating for good sanitation at the landing site whereby all the landing sites must have a latrine and used properly. We are also discouraging men who go to fish from defecating in the water because they may not be knowing but they are the very people who have bilharzia. We are also trying to encourage women to use clean and safe water for all the domestic work. Actually we are trying to compare and make them know the cost of treating bilharzia to the cost of paying for water. Bilharzia can cost you a lot of money or even cost you your life. A jerrycan of water is at most 200UGX.  ***Interviewer:*** How does focusing on different gender (men vs. women vs. pregnant women, fathers, mothers, aunties, uncles, grandfathers, grandmothers, girls or boys)  (At work/ by occupation/ economy, in the family, in the health facility, or in political administration) help improve access to and utilization of PZQ?  ***Interviewer:*** Let’s begin with the family  ***Respondent:*** In the family we can do assessment. The VHTs know these people well they know who is more empowered, who would they approach and succeed, either men or women. So they go to that person then the person can help influence others in the home.  Then in the community, we should engage community leaders of all sexes to gain entry. When you enter the village through leaders of different gender then all of them will also speak. Like I said members in the community are given choice to choose which one to go to either male or female. Also ensure that when choosing leaders political and health leaders including VHTs they should be of both sexes to ease entry and access to both sexes.  ***Interviewer:*** How can focusing on gender help us improve access and utilization of PZQ in the health facility?  In our facility we have 2 clinical rooms and two clinical officers male and male. We ensure each sits in any clinical room and then patients are given a choice of where to go and who to see. Gender is paramount at the health facility. Some patients are not comfortable being seen by a member of the different sex and may not speak freely. Even on admission some women prefer men some fellow women.  ***Interviewer:*** Do you get men who prefer to been seen by female clinical officers  ***Respondent:*** Yes they are there.  ***Interviewer:*** How would the gender knowledge help government to plan better for PZQ access and use?  ***Respondent:*** They should think of rural communities. When doing so they should zero on women because they are the seekers of treatment for themselves and children. Think of pregnant women, grandmother, etc. and plan better how these can be accessed at their various localities. They should also think of men who are drunkards, who wake up every morning and go to drink coming home late hoping women have solved all the problems. They should also pass by football clubs, drinking joints, cinema joints and so on where men gather for leisure to capture them for distribution and education. Men do not have time to go where the distribution is occurring.  ***Interviewer:*** How about at school how does thinking about gender help us improve PZQ use?  ***Respondent:*** In schools I think the response to PZQ is good, gender has no much effect. However, some schools have mature ladies and there are some who are pregnant, others dodge these drugs thinking they might be pregnant. There should be plans for screening for any pregnancy and ruling out the assumptions. Side effects worse with women. There should plans to manage the side effects with women. Also the problem of food which is across all genders causes children to dodge the drugs. There should be plans at school to give children something to eat before they take medication.  ***Interviewer:*** Do you have another chemotherapy apart from PZQ?  ***Respondent:*** No, it is only PZQ.  ***Interviewer:*** What changes in gender (roles, responsibilities, behaviors, expectations, or individual characteristics linked to a perceived sex identity) do you think can improve preventive chemotherapy or WASH in Pakwach?  ***Respondent:*** One is we are to scale up advocacy for good hygiene and sanitation practices that is including use of latrines. Then, massive community health education to make them appreciate that bilharzia can be got from contact with contaminated water then they avoid contact in any way they can. The third on is dousing rivers with chemicals which is not harmful for people because as explained poverty is high so avoiding contact with water is hard.  Another suggestion is target women and men to form groups and train and sensitize to sensitize the community about bilharzia, PZQ up take and promoting WASH.  ***Interviewer:*** What must change in cultural gender norms?  ***Respondent:*** The thinking that women are the sole care taker of home. We have to change that, women and men should share the responsibilities of the home.  ***Interviewer:*** Do you have any comments or suggestions?  ***Respondent:*** I have one question, can we get the feedback from this survey?  ***Interviewer:*** Yes, we can be able to avail with a report. Otherwise if you remember anything let me know please.  ***Respondent:*** Alright, thank you  ***Interviewer:*** Thank you so much for all this time. |
