# Supplementary material for: Gendered lives, gendered Vulnerabilities: An intersectional gender analysis of exposure to and treatment of schistosomiasis in Pakwach district, Uganda
Source: PLoS Negl Trop Dis. 2023 Nov 10;17(11):e0010639. doi: 10.1371/journal.pntd.0010639 (PMC10684070; doi:10.1371/journal.pntd.0010639)
Supplement: S1 Data — (ZIP) [file pntd.0010639.s001.zip › KII Schisto Interviews/KII Mr. Okumu Noah.docx]

***Study title:*** Gender intersectionality

and

Schistosomiasis in rural Uganda

***Interviewer:*** *Nakiranda Salama*

***Respondent:*** *Mr. Okumu Noah* ***Position/Designation:*** *District* *Vector Control Officer*

***Proceedings;***

- *Interviewer welcomes Respondent*
- *Interviewer introduces herself*
- *Introduces the Project and Project Leads*
- *Funders*
- *Reminds Respondent of some crucial ethical considerations (Note: Respondent had signed the consent form)*

***Grand Tour Question:***

*How does gender intersect with other factors towards influencing preventive chemotherapy and WASH interventions in Pakwach?*

***Interviewer:*** Can you please tell us about yourself?

***Respondent:*** My name is Okumu Noah. I am a male. I am the District Vector Control Officer in Pakwach District Local government in West Nile region formally Nebbi District.

***Interviewer:*** What is role of focus as a Vector Control officer?

***Respondent:*** Vector Control officer, focuses on Tropical Diseases especially the neglected Tropical diseases.

***Interviewer:*** What is your role are Schistosomiasis control/prevention?

***Respondent:*** I ensure coordination and planning for surveys in the communities to ensure that there is health education on prevention and control of schistosomiasis both in the community and health facilities and to ensure that where provisions are I do plan and coordinate for mass drug administration for schistosomiasis.

That is directly but another role is concerning research on the effects of Schistosomiasis, research concerning the effects of the diseases, like how are people affected, how is transmission going on, where resources are available, we also do that.

When it comes to health education, the component of control, the component of water and sanitation are very important.

***Interviewer:*** What policies guide his/her work?

***Respondent:*** There are policies that are in place for the public officers like the standing orders that we work along. There are policies by ministry of health that is how far we go.

***Interviewer:*** What policies guide his/her work in relation to Schisto?

***Respondent:*** There are guide lines that guide work in relation to schistosomiasis and they are under vector control division which is under the ministry of health. Those guidelines and they stipulate issues to do with the treatment levels, who should be treated and who should not, when, how and why. All those guidelines but they are not specifically for Schistomiasis but they are for all the neglected diseases of which Schistomiasis is among.

***Interviewer:*** What are the key predisposing factors to schistosomiasis?

For the general population;

***Respondent:*** One is the general population has that free access to water bodies, the lake and the river. Lake Albert and the Albert Nile. So that access is through the activities that they are involved in for example fetching water, washing clothes, fishing, and snail mining. Then also issues of swimming and bathing. There people who swim and bath from the river.

***Interviewer:*** What are those factors that make men more vulnerable?

***Respondent:*** Men, since our society has dedicated that men tend to be the bread winners, the main economic activity here is fishing therefore men tend to do a lot of fishing .They spend most of their time and their life in the lake so that they can get fish to sell to provide for their families.

The other economic activity is snail mining, the snails are mined mainly for chicken feeds so they dive deep in the lake to collect those snails.

Another one they love is bathing and swimming in the lake. In the hot hours of the day they just go into the river or lake to swim.

***Interviewer:*** What are the predisposing factors for women specifically?

***Respondent:*** The water coverage, clean water access is very low so most of the women have to go the lake or rivers to fetch water because clean water is very low in the district. Some women also carry clothes and utensils to go wash from the lake.

Another group is that of business women who go buy fish from the lake and the river to go sell to the customers. In that process they come to the river and get in contact with the water.

***Interviewer:*** Is it any different for the pregnant women?

***Respondent:*** Pregnant women, not as such because they are involved in the same activities that those that are not pregnant do.

***Interviewer:*** At this present time, how possible or realistic is it to prevent skin contact with high-risk schistosoma waters for each gender type? Give reasons for your answer

**For the Females;**

***Respondent:*** You know that the possibility of that happening is far too, I think it is impossible for now. It may be possible only when there is an alternative way of accessing clean and safe water. But still for those involved in the selling fish it is still hard. There are also those believes that processing the fish for domestic use or preparing is better with water from the lake so still unless the mind set of people is changed which is hard, preventing their contact with risky waters seems impossible.

**For males;**

***Respondent:*** Like for women it is very hard, it is really impossible even we have seen projects that the government has tried. Introducing modern methods of fishing but that has failed. They are used to those methods they grew up using and they cannot do away with fishing of course.

**Pregnant females**

***Respondent:*** As earlier said, the pregnant women still operate as those that are not pregnant.

***Interviewer:*** What are the nature of treatment seeking behavior with regard to Schistosomiasis?

The treatment is not so bad especially when administering mass drug administration.

***Interviewer:*** What about going to health facilities?

***Respondent:*** People do not have that initiative to say let me go and test, they always think of other things. It is hard to find someone say thet am not feeling well let me go and test for bilharzia.

***Interviewer:*** What gender issues affect treatment seeking behavior of schisto patients?

***Respondent:*** Men are generally have poor health seeking behaviours, they don’t love being at health facilities. They tend to be busy all the time like concentrating on fishing and making money.

For women, still with bilharzia, it is rear because they hardly suspect, they can go other diseases. They also tend to be busy at home doing house chore.

***Interviewer:*** How does being female or male gender or others (that’s is man; woman, mother/ father, pregnant mothers) influence behavior change and praziquantel uptake towards better control of schistosomiasis in your district.

**For males;**

***Respondent:*** For males, they generally have poor seeking behaviours that makes hard among men yet they have the power to influence, they can make decisions independently, and they can make decisions concerning their health and follow them up. But because of the poor seeking behaviours and the attitude towards health is a challenge. At times they come to seek treatment. By virtual of the positions in society they can influence change in society, if they take lead by taking medication or deciding that we should not use water bodies or saying that we have to construct latrine and stop open defecation but if he is the same man who says I cannot take medication.

**For Women;**

One, if we are to look at the family level and the nature of women. One is that women are family councilors they can influence the family to take PRQ or beginning with even the family head, they can influence them. Actually more of the health information is held by women. Women are always at the health facility. Women relatively have a good seeking behaviours. They are actually good, and if there is any programme because men do not stay home but women are mostly home and if you want to talk to a community, the bigger number you will find are women. If there are community dialogues, women will attend more than the men. Health information or any other is mostly received by women. They can also easily talk to the family about health information. Even when it is an outreach to treat any disease.

***Interviewer:*** Can you please tell us about your experience in implementing interventions to control schistosomiasis in your community?

***Respondent:*** My experience is that I have had a lot of challenges with behavioral change but I got to discover that it takes a process but some factors are beyond us for example when it comes to access of clean water it is beyond us, when it comes to latrine coverage and other sanitation issues it is something to with community they must first change their attitude towards certain things to see that they have them like latrines.. When it comes to adherence to treatment drug administration people have different perspectives towards bilharzia, some fear the reaction of the medication or the side effects. Others feel that this drug is not ok and they cannot take it. Some even say that they are not sick so why are you treating them. For the three years I have realized that people need to be talked to about the benefits of the drug. Health education for me, is one of those basics which I think can bring a change.

***Interviewer:*** Praziquantel mass drug administration is one of the key interventions for treatment, control and prevention of schistosomiasis. Please comment on it.

**1, Access**

***Respondent:*** I would say the drug is inaccessible. Getting the drug is hard. First, in our essential drug supply through the Ministry of Health and National Drug Authority it is not on the list. We just receive it as a donation that people just have to offer especially from World Health Organisation. It is not even present on the open market and if it is there it is very expensive that none of the community members can afford. There if one has it, she or he would have to wait until there is mass drug administration. That’s why we have people vomiting blood but no medication.

**Interviewer:** How has PZQ performed with regard to prevention and treatment?

***Respondent:*** For prevention and treatment it has done very little, for the few surveys we have had, the disease seems to be getting worse although we have not had some cases like people having extended abdomen. But you find that when you test there are many cases than before. Even drug administration has not done much to prevent or control the disease.

**Interviewer:** What would you do better (generally)?

***Respondent:*** There are a number of things and one is advocacy and seeing that we communicate issues of bilharzia to people who can come in to support such as people involved in sanitation for support. Health education is also necessary, the community need to know their role in preventing the disease. They have a role to play not just thinking of preventive chemotherapy, we cannot dig latrines for all of them. We cannot go and fetch water for them. They have to see that they go get clean water or they avoid contact with contaminated water or minimize it.

I think the health education should also focus on people coming for treatment and encouraging them to know their statuses (Schisto-status).

We would lobby to be supported in this area such that we to extensive and frequent health education to the community other than just chemotherapy.

***Interviewer:*** What would you do better focusing on different gender (men vs. women vs. pregnant women, fathers, mothers, aunties, uncles, grandfathers, grandmothers, girls or boys)

*(*At work/ by occupation/ economy, in the family, in the health facility, or in political administration) help improve access to and utilization of PZQ?

***Respondent:*** For men; they need to be educated because they are more at risk since they do fishing, swimming and bathing in the lake. They need to protect themselves and their families. There any more men who come to health facilities at late stages while vomiting blood. Basically in all areas awareness campaigns is a huge solution.

For women, sensitization is still very important. Talking to them about the risks, providing them wither clean water access.

In the health facilities it is to improve on our diagnosis capacity like diagnosing stool, and also include suspicion, a doctor what do you do, as a lab technician what do you do. Everyone who comes to the facility at the first test has to be tested for bilharzia like it is done for HIV.

***Interviewer:*** What changes in gender (roles, responsibilities, behaviors, expectations, or individual characteristics linked to a perceived sex identity) do you think can improve preventive chemotherapy or WASH in Pakwach?

***Respondent:*** Involving men to take up responsibility to protect their families such as construction of latrines. Women cannot take up some responsibilities such as constructing latrines. They have to take up the lead because they can do those activities but they just leave everything.

Women can guide the community more than the men. If men would allow women to take up the lead concerning health issue the conditions would surely improve. As had earlier said women have more health information they are most times at the health facilities taking children. So they should be left to make the decisions concerning the health of people in the homes and also be financially supported by the men.

***Interviewer****:* Do you have any comments/recommendations/suggestions?

***Respondent:*** I think I would draw the recommendation from the different discussions we put forward like right now If the likes of the school of women and gender studies, ministry of gender come up with the wellbeing programs for women, children and men all together to see that the health of the women and the men are like and even the children if we raise our voices together by engaging and focusing on the wellbeing of all genders not just women, we need to focus on women because they are the backbone of our economy, children are the future but men are also very important in our economy. If we do that, we could do better to reduce on the predisposing factors.

We also need to focus on the area of awareness.
