# Supplementary material for: Gendered lives, gendered Vulnerabilities: An intersectional gender analysis of exposure to and treatment of schistosomiasis in Pakwach district, Uganda
Source: PLoS Negl Trop Dis. 2023 Nov 10;17(11):e0010639. doi: 10.1371/journal.pntd.0010639 (PMC10684070; doi:10.1371/journal.pntd.0010639)
Supplement: S1 Data — (ZIP) [file pntd.0010639.s001.zip › KII Schisto Interviews/KII Mr. Shaban Ofoi.docx]

***Study title:*** Gender intersectionality

and

Schistosomiasis in rural Uganda

| ***Interviewer:*** *Assoc. Prof. Sarah Ssali*  ***Respondent:*** *Mr.* *Shaban Ofoi*  ***Position/Designation:*** LC3 Chairman(Panymur Sub-County)  ***Proceedings;***   - *Interviewer welcomes the respondent to the interview* - *Interviewer introduces herself* - *Introduces the Project and Project Leads* - *Introduces Funders* - *Reminds Respondent of some crucial ethical considerations (Note: Respondent had signed the consent form)*   ***Grand Tour Question:***  *How does gender intersect with other factors towards influencing preventive chemotherapy and WASH interventions in Pakwach?* |
| --- |
| ***Interviewer:*** Could you please tell us about yourself?  ***Respondent:*** My name is Shaban Ofoi, am 40 years old male. I am the LC. 3 Chairman of Panyamur Sub-County Pakwach district. The lower local government is led by an LC3 or mayor in municipality. Panyamur is located along L. Albert. As the LC3 chairman I play similar roles as the President at national level and LC5 at district level. The difference is the size of jurisdiction and power. Upper local government have others roles. LC3 also mobilizes populace to conduct government interventions, makes policies and bylaws to run affairs at LC3 level.  ***Interviewer****:* What policies?  ***Respondent*:** Generating policies from a national policy for example customizing the national policy. LC3 is also responsible for the protection of lives of people under his/her jurisdiction and budgets to run affairs at the level of sub county. The president causes the budget to be presented to the Parliament of Uganda the LC3 does the same at the level of the S/C. He/she does budget presentation, approval and monitors and supervises implementation.  ***Interviewer****:* What is your work in relation to schistosomiasis?  ***Respondent:*** I Partnered with government and NGOs in relation to distribution of PZQ for schools. I also mobilize communities to ensure medicine is taken and do school visits to encourage teachers to administer medicines and ensure pupils take the medicines.  Sometimes I Interface with parents to discourage them from getting into the lakes and rivers.  I ensured that sources of cleaner and safer water were installed. I have written projects to supply piped clean and safe water on landing sites to reduce people’s contact with the water. I have encouraged clearing stagnant water including swamps. Areas that connect from lakes to such water points can be breeding grounds and school children play into such ponds near homes which makes them vulnerable. I also engage in sensitizing people on Schist and to constantly test. We are conducting a national programme that is rolled through the Ministry of Health and it is under the office of the DHO.  ***Interviewer:*** What are the key predisposing factors to schistosomiasis?  **Generally:**  ***Respondent:*** Lifestyle and Livelihoods. People are predominantly fishermen and you know fishing is supposed to be done in the water and not out. The methods they use require them you to get into the water and cast your nets in the water therefore they walk into water bodies lakes, swamps and rivers.  ***Interviewer:*** Is there anything else that makes them have to enter the water?  ***Respondent:*** Yes, also collecting fish shells for chicken feeds especially by women. They get into the lake to scoop this which is very dangerous as they are in stagnant water points with limited waves that provide breeding grounds for organisms that cause diseases like schistosomiasis.  Women also go to fetch water, house chores the domain of women. Previously, most areas didn’t have clean and safe water. Coverage of clean and safe water was 35%. The biggest water sources was the lake. 70% of the population used lake as source. Now, there over 90% coverage of clean and safe water. All areas have tap water in such places. This has reduced contact with water. Poverty is also a big problem, people don’t do these things because of choice. They don’t have alternatives, and it’s about survival. Some fetch water for others to earn a living. Others to wash others clothes to get a meal for a day (largely females). Another thing is customs and traditions: People stick to customs and traditions. Some think drinking water from lake or river is the best like how their grandparents did. Some Bathing in the water, especially the younger ones, even older people largely males. Women mostly bath from home. But because of low income they can’t rely on tap water for drinking and using it for drinking and washing plates. Mixing the two leaves the danger. Same jerrycans for fetching lake water are used for safe water.  ***Interviewer:*** What of pregnant women, are they at the same risk?  ***Respondent:*** Whether pregnant or not she is still a woman, and until they reach the last stage they have to continue working and are exposed to danger. They are women according to tradition and it’s dangerous.  ***Interviewer:*** At this present time, how possible or realistic is it to prevent skin contact with high-risk schistosoma waters for each gender type? Give reasons for your answer  ***Respondent:*** It’s not ease because the danger is from the work engaged in especially the males. Females are exposed to water in the process of processing the products from fishing. Fishing is the work of men. Processing is largely the work of females. Females also engage in domestic work for families’ sustenance, they buy fish products, process it by cleaning it in the water thereby, they get into contact with the water. Plus others mentioned above. Putting up clean and safe water reduced those who drunk lake water directly, now they drink tap water. Reduced exposure especially of the young children. But it has not eliminated contact with water because it’s their livelihood and source of living.  **Interviewer:** What are the nature of treatment seeking behavior with regard to Schistosomiasis?  **Respondent:** The challenge is that by the time you think you have bilharzia it could have stayed in the body for a very long time. Normally when it is in the advanced stages. Both male and female, at the point when one begins getting diarrhea, extended belly and vomiting blood. Most fear the test, often because of the effect of the medicine. The cohort better placed to be attended to are largely school going children because they are taken to schools and pupils have to take the medicines at school. The older tricky to deal with as they don’t’ voluntarily test. These are habits in Africa. People go to health facilities when sick, by which time they have liver sclerosis or vomiting blood. It can be 10 years plus before getting help. Household habits are not a challenge. Women are a bit strong when it comes to sickness or diseases however, they take long to seek medical advice. Also they may not have the money since the money is with the male. By the time she says she is sick is when she is really sick. Otherwise she prioritizes many things, self-medicating to affect the symptoms a lot without getting better.  **Interviewer:** So what about the men?  **Respondent:** Men seek medication faster because they have the money. Seeking medical advice does not have diverse effect on the wellbeing of the family. Masculinity is a challenge. Men also self-medicate for the symptoms instead of digging deeper why the symptom.  **Interviewer:** How does being of female or male gender or others (that’s is man; woman, mother/ father, pregnant mothers) influence behavior change and praziquantel uptake towards better control of schistosomiasis in your district?  **Respondent:** Behavior changing is there because of the effects of the disease such as vomiting blood and having liver sclerosis are high and in hospitals they are being told this is bilharzia. The days of protruding bellies have reduced. The cases of vomiting blood is what has gone up and all others relating to bilharzia. So they are learning the hard way, through death and chronic diseases.  Females pick messages faster and respond to them faster than the males. Males are a bit resistant and slow in picking a medical message. Men ask a lot of questions, is it a male or female medical doctor, nurse, etc. But a female person takes the message the way it is first, as a true message and requires response the way it is said.  **Interviewer:** Please comment on taking PQZ for curative?  **Respondent:** The drug has been there for long. All the years. These medications are not enough, are donor supported, not provided by government, not available all the time hence default on the doses. There is fear of the effect and contraindications.  **Interviewer:** What is the gender dimension of PZQ uptake? Drugs are given at the health facility. **Respondent:** Men are rarely at the health facility save if it is big and major issue. The drugs tends to help the female most as they go more frequently for their own illness or children’s illness. Females are more exposed to these messages than their male counterparts. Also more likely to take the meds than the males. The diseases may not be an epidemic but the effects are long term and people are dying.  **Interviewer:** Can you please tell us about your experience in implementing interventions to control schistosomiasis in your community?  **Respondent:** Some people are receptive, they get the message for behavior change. Some take the medication especially females and the young. Exposure is also from fishing and there more fishermen than fisher women. They are not farmers. Therefore, daily they are exposed to contact with water, which exposes them to the disease. Due to poverty there are no alternatives the only way to survive is by fishing. Medications are not readily available due to finance constraints. There is also fear of taking medications due to diverse effects. Some people may have taken as a children. Me in the early 2000s, I was told to eat and take after the meals. I took all the four at once and slept till morning. Such effects are common to all. The medical fraternity need to dig deeper and find out the different effects on different people. Some customs and traditions are not good in the medical fraternity. Some grannies died of effects of bilharzia. When signs and symptoms are factored in you realize the person died of bilharzia. Bilharzia is part of the neglected diseases. So there is need to raise the voices bigger and higher to address the silent killers.  For pregnant women, medics need to dig deeper from the effect of PZQ on pregnancy. A women would fear from the other reasons .There are medical reasons and contradictions for pregnant women not to take PZQ.  **Interviewer:** What would you do better?  Respondent: Don’t look at bilharzia in isolation but in combination with outer effects of life. Some cases are out of livelihood and habits. Others from poverty or low income levels. It affects the poorest families. There is need to fight poverty as a holistic approach in uplifting people.  **Interviewer:** Focusing on different gender (men vs. women vs. pregnant women, fathers, mothers, aunties, uncles, grandfathers, grandmothers, girls or boys)  (At work/ by occupation/ economy, in the family, in the health facility, or in political administration) what would you do to help improve access and utilization of PZQ?  **Within the Family**  ***Respondent:*** Encourage families to embrace testing even when not sick. Regardless of gender and age, voluntary testing should be routine, taking medications construct alternative sources of livelihoods to minimize contact with water bodies.  **In work places**  ***Respondent:*** Everyone fishing needs to take the medication. At the landing sites. There should be a medical doctor to show people how to take the medication. Outreach programs using health facilities in parishes and sub counties are also necessary.  **In the health facility**  ***Respondent:*** Outreaches should be embraced. Get the machines and get out. Get services closer to the families. Move to them if they are not coming to the facilities. It has been done with schools, to test pupils. A high percentage of pupils tests positive. Test results conducted by the district teams was appalling. There were a number of kids with eggs in fecal content.  **In your community**  ***Respondent***: Taking preventive medications for all to show leadership to ensure communities pick it up seriously  **In government**  ***Respondent***: Government should provide the medication in the facilities. Government and people have neglected the diseases. VHTs can be taught how best to administer the drugs and households  ***Interviewer:*** What changes in gender (roles, responsibilities, behaviors, expectations, or individual characteristics linked to a perceived sex identity) do you think can improve preventive chemotherapy or WASH in Pakwach?  ***Respondent***: Males should change their behaviours from thinking they are the strongest sex who cannot take medication. Men should take the lead in taking the medications. Should also get involved in support of the families, not to leave it to the females. Men should be champions of their own societies. Men need to embrace the message regardless of the bearer. Men should spearhead issues of gender. If all choose to do the right thing, there should be no right for gender insensivity.  ***Interviewer:*** Do you have any comments or suggestions?  ***Respondent***: We all know where these cases are. One with certain symptoms such as those of bilharzia needs serious attention. These are long term illnesses. This eats into the economy of the country, family economy, and leaves many children orphans. Ripple effect is high into the economy and livelihoods. It can be taken into consideration to encourage the medication than leaving it to them to buy, even if they know they are supposed to take it. Provision of clean and safe water is needed and encourage people into alternative sources of livelihoods. Even with fishing, we can minimize wild fishing. We can encourage other fishing methods like pond fishing, fishing in cages, to try and minimize body contact. We need to empower the women, as you will empower the entire households and communities. This in the long run it will minimize the number of women who go to fish in lakes and rivers for shells. Traditional leaders should try to try to pick on customs and traditions that are bad. Creating wealth for the poor is necessary. Government should champion this fight. Cases of fish caging should be done at very minimal level because they are very expensive and they require government intervention. Under NUSAF 3 government provided fish caging programmes. But because of poor handling of these projects and high cases of corruption each group was given 17.5M to set up cages, provide fingerlings etc. Community can’t even reap 1M out of it. Money came in kind of nets, fingerlings. The outcome should have been much better. There was Fingerlings and poor quality inputs with ineffective trainings. Community group should be helped out of poverty but the interlocutors have their own interests which make the project a waste. Fingerlings were put and fed for all, the project lasted for a year. Fish couldn’t return 200UGX or 500UGX. Government needs to be at the forefront of such interventions and should be done with the aim of reducing poverty. The project should be audited. Government should check itself to guide the next phase of the project. For government programs, the prerequisite should be that at least a quarter should be female. All female it is the best. Women should own the fishing gear, boats, nets, etc. Most fish mongers are female, they process fish and take to the market. Males do the fishing because it is risky and labor intensive, sleep on the water at night, motorized boats, rowing sticks. They provide oversight and monitor government programs and NUSAF was domain of the district. Our role is to generate interests and have them registered, convene meetings to register projects and then submit to district and Ministry of Gender, Labor and Social Development. The district cannot audit because it was the culprit, the mother district needed to do the process of project implementation. The learning process for the next phase of the projects. They should manage caging and processing. Splitting and brushing within the lake should be discouraged. Fish from cages should be processed and sold fresh, refrigerated and taken to the market. Body contact with water is longer with fishing than with washing.  ***Interviewer:*** It has been great. Thank you so much for your time |
