# Supplementary material for: Gendered lives, gendered Vulnerabilities: An intersectional gender analysis of exposure to and treatment of schistosomiasis in Pakwach district, Uganda
Source: PLoS Negl Trop Dis. 2023 Nov 10;17(11):e0010639. doi: 10.1371/journal.pntd.0010639 (PMC10684070; doi:10.1371/journal.pntd.0010639)
Supplement: S1 Data — (ZIP) [file pntd.0010639.s001.zip › KII Schisto Interviews/KII Mr. Vincent Binega.docx]

***Study title:*** Gender intersectionality

and

Schistosomiasis in rural Uganda

| ***Interviewer:*** *Assoc. Prof. Sarah Ssali*  ***Respondent:*** *Vincent Binega* ***Position/Designation:*** *Comprehensive nursing officer*  ***Proceedings;***   - *Interviewer welcomes the respondent to the interview* - *Interviewer introduces herself* - *Introduces the Project and Project Leads* - *Introduces Funders* - *Reminds Respondent of some crucial ethical considerations (Note: Respondent had signed the consent form)*   ***Grand Tour Question:***  *How does gender intersect with other factors towards influencing preventive chemotherapy and WASH interventions in Pakwach* |
| --- |
| ***Interviewer****:* Good evening Dr. Vicenti  ***Respondent:*** Good evening to you  ***Interviewer****:* Can you please tell us about yourself,  ***Respondent:*** My name is Vicent Binega. I am 36 years old and a staff of Panyamur health centre IV. I am on study leave but due to this corona, we are back to service. I am a vomprehensive nursing officer but am back to school doing clinical medicine and community health at St Augustine University  ***Interviewer****:* How long have you been a comprehensive  ***Respondent:*** I have been to Pakwach health center IV since 2014 and transferred to Panyamur in 2018. While in Pakwach, I worked with the team of Bilharzia control, taking sample from GIT bleeding, distended abdomen which were clear signs of infestation of Schistosomiasis. We did this for almost two year.  ***Interviewer****:* What are the key predisposing factors to schistosomiasis?  ***Respondent:*** Economic activities and livelihoods are too much attached to the water. Fishing is the main economic activity for men. Most of people live along the R. Nile and most of the activities that involve water are done at the lake e.g. clothes washing bathing, etc. After coming back from the garden, women go to the lake to fetch water. The attachment to water is so high and most men can’t spend a day without going to the water body because they have to go fishing.  ***Interviewer****:* What predisposes women?  ***Respondent:*** Using lake water for domestic purposes like fetching water for cooking, clothes washing at the river bank. They take time standing in the water as they are washing clothes. When they go to fetch water, they take time standing in the water as they fill their jerrycans and swim first as they collect water.  ***Interviewer****:* Is it the same for pregnant women?  ***Respondent:*** I think it is the same, in the first and second trimester pregnant women still do the same domestic work. They are at least spared in the third trimester because that is when they are heavy. They stand in water for long time. They don’t know the danger, they do it ignorantly.  ***Interviewer****:* Is there any way to prevent them from contact with water?  ***Respondent:*** Yes there is, recently government brought up a number of projects especially provision of safe water. Piped water. This has prevented women somehow from getting into contact with the water. But for the males piped water has not worked because what takes them to the lake is fishing not water. There is piped water in Alui dry corridor, NWSC, in Pakwach , Panymuir and Wadilai  ***Interviewer****:* What are the nature of treatment seeking behavior with regard to Schistosomiasis?  ***Respondent:*** You know the behaviours of our people, when diagnosed they take drugs, they don’t fear but if it preventive some of them fear to take because of side effects. Before they come to hospital for diagnosis, they begin to get dysentery, vomiting, etc. When they get such signs they are forced to come to hospital for treatment.  ***Interviewer****:* How normally comes males, females or all  ***Respondent:*** People don’t come specifically for prevention. They come with sick. Even the males come when get signs they come for treatment. Females have better seeking behaviours than the males. Men present themselves later than women. Men wait for the condition to intensify first and is going to affect their economic activities.  How does being of female or male gender or others (that’s is man; woman, mother/ father, pregnant mothers) influence behavior change and praziquantel uptake towards better control of schistosomiasis in your district.  **PQZ for curative:**  Once both sexes have come they use them equally.  **PQZ for prevention:**  If preventive, females take more compared to males because men tend to ignore compared to women. Sometimes when we go for outreaches in villages, we find women and children eager to take the medication. Sometimes even when we follow men at landing site and drinking joints they ignore us. They take medication when we use Direct Observation Treatment (DOT) but if you just leave the drug with them, they may not take. But sometimes when called upon they try to pay attention.  ***Interviewer:*** How does gender affect behavior change?  ***Respondent:*** Basing on the economic activity of the sexes, males are rigid to behavior change especially if they are told that their economic activity predisposes them to schistosomiasis. They see it as something they grew up doing except if alternatives are provided for them. For females, the alternative was piped water and to some extent it has reduced the effect of water contact which would have predisposed them to schistosomiasis. Although, sometime they are resistant to taking PZQ because they are permanently at home and they think if they had no contact with water there is no need to take the drug.  ***Interviewer****:* Can you please tell us about your experience in implementing interventions to control schistosomiasis in your community?  ***Respondent:*** Schistosomiasis has been there. Prevention has been there, PZQ and community education have also been there. Most times VHTs have been used to distribute PZQ and people have been educated on proper usage of water. So the communities are aware but the percentage is not that far. Poverty is also a contributing factor because people have to think of their livelihoods are first before thinking of the disease. They know the diseases has cure so they do not take it as a threat to life. Even if contracted it they, they know they will get healed. Hence they continue with their economic activity activities. With regard to prevention, the government and NGOs have tried to intervene enough.  Mostly we get involved with Implementing partners come with specific programmes e.g. the GIT bleeding. They have finance and support to implement the programmes but they have time frames.  ***Interviewer****:* What have been the successes of PZQ mass drug administration?  ***Respondent:*** Has been in used for more than 10 years with some success and some challenges  Rate of GIT bleeding not like before, I remember the rate of death in 2004&5 the death due to GIT bleeding was high but it has reduced though, I cannot tell the figures not clear but it is fairly reduced. If you move to communities near landing sites (men with extended abdomen) or Even at the health facilities where they do stool analysis show that results of Schistosomiasis have reduced. The interventions by Implementing partners in provision of programmes like provision of PZQ and information has helped to reduce bilharzia in the community.  ***Interviewer****:* Anything else about the success?  ***Respondent:*** WASH has also done well in home improvements and sanitation. They implement sanitation programmes in homes teach people to dry water before. They do health education, give information, teach people practically what they are supposed to do to improve sanitation in their home by using local household items instead of bringing others, they made with homes in the community tippi taps using small jerrycans and this is used to wash hands. When people use their own, they tend to sustain them.  ***Interviewer****:* What would you do better?  ***Respondent:*** The routine is not maintained. You find that the IPs that take up prevention are not consistent. Drugs take like 6 months and 1 year not available, are VHTS not motivated. It would be better if IPs follow the routine like providing PZQ after every 2, or 4 months like ANC visits.  Information is also important, people forget and they therefore need repeated health information on prevention so they can follow and do what is necessary on their own to prevent schistosomiasis  ***Interviewer****:* Focusing on different gender (men vs. women vs. pregnant women, fathers, mothers, aunties, uncles, grandfathers, grandmothers, girls or boys)  (At work/ by occupation/ economy, in the family, in the health facility, or in political administration) help improve access and utilization of PZQ?  ***Respondent:*** Females will always embrace use though men are more at risk. Men have poorer health seeking attitude these are the upper adolescent and adult males. Lower adolescents, 15 years and below are better at health seeking since they are controlled by females.  ***Interviewer****:* What interventions can we do in the family to improve gender access?  ***Respondent:*** Involving the heads of the families. Make visits to individual homes. Information should be communicated earlier and clearly about the home visits and that heads of homes must be available to home. Head of family, woman and children should be at home otherwise the man would say I will find mine at home. Like the time of census, the whole family would be found at home and also like cholera vaccination where we used to get men, women and children all waiting at home.  ***Interviewer****:* And what would you do better in your work place/health facility?  ***Respondent:*** We integrate it into the treatment every person who comes for treatment should go home with PZQ whether they have tested positive for bilharzia or negative. I used to do it even without the knowledge of the patient.  ***Interviewer****:* What would you recommend in their work places?  ***Respondent:*** Provide information pertaining to prevention, like how obulamu program does, pinning stickers, fliers, everywhere like at landing sites, etc. Mostly we should provide preventive information.  ***Interviewer****:* What about in the In your community:  ***Respondent:*** We should the same as in the workplace?  In government:  Intensify the availability of the PZQ, also intensify adverts about health information on radios Also integrate PZQ in prescriptions for patients in areas of high prevalence.  ***Interviewer****:* Is there anything these issues have to do with regard to gender?  ***Respondent:*** Women fear to take when they are pregnant due to the effects and they are also prevented by their husbands if they suspect them to be pregnant.  ***Interviewer****:* What changes in gender (roles, responsibilities, behaviors, expectations, or individual characteristics linked to a perceived sex identity) do you think can improve preventive chemotherapy or WASH in Pakwach?  ***Respondent:*** One of them is Involvement of both sexes in prevention. If males are involved the females will follow.  At the level of behavior, men more involved in activity which renders them vulnerable. When they go fishing they defecate in water. If they are educated that it’s wrong to defecate in water. Its better if they go fishing, they first ease themselves before entering the water. Also if they carry boiled water for drinking when going to fish.  Government should recognize the economic activity of men and provide them with preventive gears such as gum boots.  ***Interviewer****:* Anything that should be changed with regard to fishing?  ***Respondent:*** They should be put in groups and given resources for caging, so as to leave many out and only a few getting in contact with water not many people at the same time  Other fishing methods should be used, Baits are snails under water, used on hooks to catch fish. Caging reduces contact with water.  ***Interviewer****:* IPs how about, is it only IPs that provide PZQ**?**  ***Respondent:*** Government also provides but it’s not consistent, they provide like 1,000 tablets of PZQ in a quarter, yet population is 4,000 so supplies are not enough. The gap is wide compared to IPs. Government tends to withdraws when they realizes that IPs are providing and when IPs have left it takes time to take over from IPs. Government does take bilharzia seriously.  ***Interviewer****:* How big is the doze of PZQ?  Dose of PZQ is 4 tabs in a quarter for an adult and children from 5 and below (1/5 a tab).  ***Interviewer****:* Do you have any comments or suggestions?  Get on ground and move to communities to address some unanswered questions. The doctor who came for research collected samples has never come back and people have never received results of the research. They had a high expectation that a better solution would be got for their problems, up to today none has explained to them the problem. Some IPs come in the name of research and go for good without coming up with any solutions or findings. If Involve ourselves in community, we should ensure to leave some hope in them.  ***Interviewer****:* **Thank you very much for this time.** |
