# Supplementary material for: Gendered lives, gendered Vulnerabilities: An intersectional gender analysis of exposure to and treatment of schistosomiasis in Pakwach district, Uganda
Source: PLoS Negl Trop Dis. 2023 Nov 10;17(11):e0010639. doi: 10.1371/journal.pntd.0010639 (PMC10684070; doi:10.1371/journal.pntd.0010639)
Supplement: S1 Data — (ZIP) [file pntd.0010639.s001.zip › KII Schisto Interviews/KII Mr. Wanican Benjamin.docx]

***Study title:*** Gender intersectionality

and

Schistosomiasis in rural Uganda

| ***Interviewer:*** *Assoc. Prof. Sarah Ssali*  ***Respondent:*** *Wanican Benjamin* ***Position/Designation:*** Senior Clinical Officer incharge (Panyamur HC3)  ***Proceedings;***   - *Interviewer welcomes the respondent to the interview* - *Interviewer introduces herself* - *Introduces the Project and Project Leads* - *Introduces Funders* - *Reminds Respondent of some crucial ethical considerations (Note: Respondent had signed the consent form)*   ***Grand Tour Question:***  *How does gender intersect with other factors towards influencing preventive chemotherapy and WASH interventions in Pakwach?*  ***Interviewer:*** Can you please tell us about yourself?  ***Respondent:*** My name is Wanican Benjamin. I am 56 years and a male. Am a Senior Clinical Officer and Health Educator and in charge of Panyamur HC3 in Panymur Sub-County, Pakwach District. Am also the district Surveillance Focal Person for Pakwach district. I head general diseases surveillance though the focus is on COVID19 I have Been at this position since 1998, when in Nebbi and Pakwach became a district in 1998.  ***Interviewer:*** What does Surveillance involve?  ***Respondent:*** It involves monitoring the disease trend in the district and these are diseases of epidemic nature. Coordinating the district surveillance activities. Capacity building of other staff in disease surveillance and active search for some diseases such as vaccine preventable diseases and overall coordination of disease surveillance in the district. Also a clinician of Pakwach HC3. Most of my work is in the community.  ***Interviewer:*** What are the key predisposing factors to schistosomiasis?  ***Respondent:*** Let me begin with community ignorance. They are just ignorant that they think that schistosomiasis has been here and therefore don’t look at it as a disease which can be major problem. Unclean water the Nile is the major source of water. From time to time they fetch water from the Nile for domestic use. The other is economic activities the main economic activity is fishing as they go fishing they are exposed to bilharzia. Also the problem of Poverty – They don’t take the initiative to boil the water despite the training to boil or sterilize the equipment given to homes with high prevalence. Once the solar sterilization is spoiled no one can take the initiative to repair it they go back to drinking the wrong water.  ***Interviewer:*** Is the equipment given to every household or it is communally known.  ***Respondent:*** It was given to every house =hold in areas with high prevalence.  ***Interviewer:*** How expensive it is?  ***Respondent:*** By that time it was between 150,000 to 200,000UGX and every household received and provision was by an NGO called Solvaten. It was supplied more than 3 year ago.  ***Interviewer:*** How is its system?  ***Respondent:*** You Fill it with water, it has some part of solar, and put it in the sun the Sun will heat the water. When it shows blue it means the water is safe enough to be used.  Is that water hot or cold  ***Respondent:*** Oh it’s hot.  ***Interviewer:*** You have been giving us the general predisposing factors to schistosomiasis are there any that affect women specifically and those that affect men?  Now for women, they fetch water for entire family. They are the ones that get water for all work needing water. Most of the women are fish mongers. They go very earl to the river either to collect water or to buy fish. There they get into contact with the water and get ingected.  Also men, most are fishermen and they throw feaces in the water. Many get to water and get infected.  The children like playing in the water. Bath directly in the water, which exposes them.  ***Interviewer:*** At this present time, how possible or realistic is it to prevent skin contact with high-risk schistosoma waters for each gender type? Give reasons for your answer  ***Respondent:*** Yes. It’s possible when you look at the different types of interventions that can be put in place.  ***Interviewer:*** How?  ***Respondent:*** If the community is told the time of transmission so that we can limit or avoid contact with water. There should also be boreholes constructed in areas with very prevalence. At the same time, we shouldn’t forget Sensitization of community members and empower them with knowledge and skills because the biggest problem is ignorance. We should tell them **to** boil and sterilize the water as well as telling them why. There should be echnical engineering aspect of latrine construction. They should be able to change their economic activities Most of econ activities like fishing make them stay in water for long. If there is an alternative econ activity which could be introduce and limit contact with water and exposure to schistosomiasis.  Solvatel method of sterilizing water can be provided again. Most could be broken. We could introduce a better method the current one only takes about 10 litres of water which is not enough for the entire family. Bigger capacity needed.  ***Interviewer:*** So these are largely methods for preventing schistosomiasis, but how can contact with water be limited?  ***Respondent:*** I talked of construction of boreholes.  ***Interviewer:*** Yes  ***Respondent:*** Also there is that time zizcaria (the worm that causes schistosomiasis) comes out of the host.  ***Interviewer:*** When does it come out  ***Respondent:*** We have been told that if you go early enough before 6 am to 7 am and late after 6.00pm it is safe. So we need to sensitize people. But these are dangerous times for women to go fetch water.  ***Interviewer:*** Does that mean the men who fish in the night are safe.  ***Respondent:*** No I have not taken any research on that.  **Interviewer:** Isn’t dangerous for women, if they are the ones who fetch water, wont they fall into other dangers  ***Respondent:*** It has not practical, we have not seen such things. Most of these things are just theoretical.  ***Interviewer:*** So then, do these things affect each gender different (the remedies you mentioned)?  ***Respondent:*** I cannot think of any other thing?  ***Interviewer:*** That’s ok.  ***Respondent:*** There is something I forgot  ***Interviewer:*** Yes please  ***Respondent:*** The distribution of PZQ as a preventive measure should be very regularly because once you give PZQ they will take it and it will reduce chances of transmission.  ***Interviewer:*** Are there are specific methods for children?  ***Respondent:*** Yes, children as I told you, one they like playing in the water we could invent alternative means for children where they can play from. Also alternatives for swimming e.g. swimming pools but I don’t know how it can be maintained though.  ***Interviewer:*** What is the nature of treatment seeking behavior with regard to Schistosomiasis  ***Respondent:*** Well treatment seeking behaviours is not only with all the diseases. It is difficult to recognize Schistosomiasis within a short time. Sometimes they come with different complications like liver problem, abdominal pain. Only stool examination will reveal schistosomiasis. They do not specifically come for schistosomiasis but general complications. Therefore, health care seeking behavior is poor in the community.  ***Interviewer:*** Is it the same across all genders?  ***Respondent:*** For Women its worse More women come for treatment. Few men seek treatment. But then when you look at this someone would stays at home with a sick child until the child starts disturbing her peace or when the child has developed some complications as I told you schistosomiasis is a chronicle disease, sometimes they do not realize it timely. Seek treatment when child has complications or is complicating her life.  ***Interviewer:*** When you say more women come for treatment do they come for their own or for the children?  ***Respondent:*** For both their and the children.  ***Interviewer:*** Do men not come even when it has been discovered?  ***Respondent:*** The OPD data shows that less men come compared to women.  ***Interviewer:*** Why?  ***Respondent:*** There are many reasons first of all men are the bread winners. They first engage in economic activity to support the family. They also want to show that they are men so they resist until the disease has made them weak enough and that is when they seek treatment. Then another area. Sometimes it’s the way of service delivery, sometimes they have to wait at the health centre to get treatment but men are impatient unless the women who always wait for service at health centre.  ***Interviewer:*** How does gender influence behavior change in relationship to schistosomiasis?  ***Respondent:*** One when you look at, in my experience what I have seen is that more women can access information but because of limitation in education the application of the knowledge is a few people are literate. More men are literates than women so when they get information they can use it faster. Culturally women are put in the backyard therefore they have limited access to information, they spend most time at home doing housework. She will have no time to move to the facility more often. Most child rearing is for the women. Also especially men prefer to send boys to schools where they get information. Women need men’s backing for finance and support e.g. transport to the facility where far.  ***Interviewer:*** Do we have men accessing this information and using  ***Respondent:*** Yes but men are very slow adopting to change. Women once they exposed to information, they can easily adopt to change. Even female VHTs do better than the male.  ***Interviewer:*** How does being of female or male gender or others influence PZQ uptake?  ***Respondent:*** No effect, all of them as long as the drug has been distributed. Only people with taboos or some people with allergies fearing reactions who don’t take.  ***Interviewer:*** Are all homes given the drug?  ***Respondent:*** Yes  ***Interviewer:*** When the VHTs are distributing, do they find the men and women there?  ***Respondent:*** When planning for drug administration, the communities are mobilized through the LCs and VHTs over the radio. As I told you men go for economic activities women are found at home with the children.  ***Interviewer:*** So what happens to the man?  ***Respondent:*** When the man is away they leave the drugs or make another appointment.  ***Interviewer:*** Can you please tell us about your experience in implementing interventions to control schistosomiasis in your community? (since 1998 )  ***Respondent:*** Thank you so much! I have been educating communities on schistosomiasis. prevention and treatment. Two is supervision and distribution of PZQ in community and schools. Then, working with landing site to construct latrines. Supporting supervision of the facilities in the district, supporting the health workers in the area of prevention and treatment.  ***Interviewer:*** In all these task what did you find them?  ***Respondent:*** Our community perception, they say they have been fishing and drinking this water for long. Apparently they see no danger of bilharzia. Even with borehole they will prefer Nile or lake water because they say borehole water is salty.  The other is dependency syndrome, most people want to depend on the donors or government. Sometimes PZQ sold in clinics but people prefer to wait for government distribution. They don’t want to buy. They could not even make community contribution as the government paid for basics. Even with latrine construction they don’t want to make contribution. Even those we completed the usage was not good. Even with PZQ administration you find that some community members have not eaten food so you leave it with them and tell them to take it when they have eaten. Another challenge was with the community distributors because they expect more money which affect the overall distribution like at school they hide the drugs and do not give to pupils and sell it out later.  ***Interviewer:*** Who refuses to give children?  ***Respondent:*** For school we train and use teacher. Some teachers refuse and it doesn’t apply to all schools.  ***Interviewer:*** What is the positive experience?  ***Respondent:*** There was increased coverage and the prevalence went down drugs free of charge. There was civil and supportive political environment created by the leaders at community/health facility level.  ***Interviewer:*** Praziquantel mass drug administration is one of the key interventions for treatment, control and prevention of schistosomiasis.  What would you comment on access:  **Respondent:** There is shortage of the drug in the facility the persons test positive but there are no drugs to give them. Some drugs are sold to the private clinics by VHTs. But the private pharmacies can’t stoke those medications because it is expensive but on low demand.  ***Interviewer:*** How much is a dose of four tablets.  ***Respondent:*** It is about 5000UGX to 6000UGX  ***Interviewer:*** Are there any other challenges you have observed in treatment?  ***Respondent:*** They complain of the reactions of the drug and some do not take because of that.  ***Interviewer:*** What would you do to improve the system if you had a chance?  ***Respondent:*** You know behavior change doesn’t take a short time so we should continue talking to them. PZQ should also be included among other essential drugs that are regularly supplied. The community distributors should be supported. We should do assessment among the distributors to find out what is the best way to support them instead of coming up with our own ways of supporting them.  ***Interviewer:*** Anything else you would do better given your experience  ***Respondent:*** If the government can put piped water to limit access to lake. We cannot take away fishing and fish mongering though.  ***Interviewer:*** What about focusing on the gender angle?  ***Respondent:*** We empower women with knowledge about the disease and other economic activities.  ***Interviewer:*** Focusing on different gender (men vs. women vs. pregnant women, fathers, mothers, aunties, uncles, grandfathers, grandmothers, girls or boys)  (At work/ by occupation/ economy, in the family, in the health facility, or in political administration) help improve access and utilization of PZQ?  Within the Family?  ***Respondent:*** We design programmes to target the females because they are the closest to the family members. They can encourage other members including their men. The best approach is to build their capacity.  ***Respondent:*** You know facility based service there is no discrimination between males and females. The only issue is the lack of the drug in the facility. So as I said let the ministry supply the drug regularly.  ***Interviewer:*** What about you as workforce  ***Respondent:*** Right from the institution, we have been trained to serve people irrespective of their gender, religion or political affiliation. But if it happens the supervisor would discuss with the health worker involved.  A clinical officer can be a female or a female so gender does not affect service delivery.  ***Interviewer:*** Are most of your VHTs male or females.  ***Respondent:*** It is almost the same.  ***Interviewer:*** Are the nurses male or female?  ***Respondent:*** There are more females than males.  ***Interviewer:*** What about in your community?  ***Respondent:*** In the community some people tend to serve their relatives better than others then also we have some political interference. Also as I told you more men are literate but most females are illiterate. We can begin with drug distributors we put a minimum qualification for someone being a drug distributor. We also need to train them. Also the government is coming up with the idea of community extension workers but the challenge will be that they won’t work on voluntary basis and their will also be few therefore they cannot cover all the households.  We should make the number of drug distributors more females than male because females always perform better than the males.  ***Interviewer:*** What would government do?  ***Respondent:*** There should be deliberate enrollment of female VHTs, they should make it a policy in areas with high schistosomiasis prevalence. They should also be guided by the how long the drug remains effective in the body while distributing it.  ***Interviewer:*** Any improvements that can be done at school?  ***Respondent:*** Let the senior woman teacher take the lead in distribution in the school but normally they give the science teachers to lead who are mostly male. Sometimes there is limited supervision by the Head Teacher.  What changes in gender (roles, responsibilities, behaviors, expectations, or individual characteristics linked to a perceived sex identity) do you think can improve preventive chemotherapy or WASH in Pakwach?  ***Respondent:*** I had talked of empowering females with knowledge and skills.  Do you have any comments or suggestions?  ***Respondent:*** At the moment, no.  ***Interviewer:*** Thank you very much  . |
| --- |
