# Supplementary material for: Gendered lives, gendered Vulnerabilities: An intersectional gender analysis of exposure to and treatment of schistosomiasis in Pakwach district, Uganda
Source: PLoS Negl Trop Dis. 2023 Nov 10;17(11):e0010639. doi: 10.1371/journal.pntd.0010639 (PMC10684070; doi:10.1371/journal.pntd.0010639)
Supplement: S1 Data — (ZIP) [file pntd.0010639.s001.zip › KII Schisto Interviews/KII Ms. Fauthum Judith.docx]

***Study title:*** Gender intersectionality

and

Schistosomiasis in rural Uganda

| ***Interviewer:*** *Assoc. Prof. Sarah Ssali*  ***Respondent:*** Fauthum Judith Kigezi ***Position/Designation:*** Health Inspector  ***Proceedings;***   - *Interviewer welcomes the respondent to the interview* - *Interviewer introduces herself* - *Introduces the Project and Project Leads* - *Introduces Funders* - *Reminds Respondent of some crucial ethical considerations (Note: Respondent had signed the consent form)*   ***Grand Tour Question:***  *How does gender intersect with other factors towards influencing preventive chemotherapy and WASH interventions in Pakwach* |
| --- |
| ***Interviewer:*** Can you please tell us about yourself  ***Respondent:*** My name is Fauthum Judith Kigezi, 40 years and am a Health Inspector  ***Interviewer:*** What does being a health Inspector Involve  ***Respondent:*** Involves community activities basically environmental health activities such as community and home health improvement campaigns, health education and other health issues like scrutiny of building plans for PH safety, etc.  ***Interviewer****:* How is your work related to schistosomiasis?  ***Respondent****:* I work with community at household level to scale up sanitation. I actually Interact with communities at house hold level and institutional level. Institutions include Schools, public offices, private institutions that are non-governmental, etc. I ensure the functionality of facilities for workers and adequacy of the facilities to environment. I look at their Structural design, how it relates to the usage or functionality of these facilities for the users.  Access or provisions that can be adequately used to enable the environment well kept.  ***Interviewer:*** What are the key predisposing factors to schistosomiasis?  **Generally:**  ***Respondent****:* There are a number of predisposing factors for people in my, Pakwach.  One, you realize that Pakwach is well endowed with water bodies, the river Nile and the lake. Hence the fisher folk in the area. Men and women are involved in fishing activities. There are those involved in direct fishing and those that involved in the buying and selling.  In this district, fishing is the main economic activity for community members. Fishing has no problem but behaviours make people vulnerable to schistosomiasis. People get into contact with water. I have never seen a fisher man go in to fish in boots, most fishermen go in shorts and bare chested hence contact with the water. Some community members don’t have adequate facilities for excreta or fecal matter, toilets and latrines. Access to fecal facilities matters. If one has schistosomiasis and uses facilities that allows contact with the outside environment, it’s bound to reach the water where our people go to fish. When it rains the waste is washed into the rain which leads to water bodies through raining etc. All rubbish is washed into the Nile.  **Interviewer:** What are the predisposing factors for men specifically?  Men are more vulnerable. By culture standards, the man must be looking after the family and do core activities. He is required to fish in night and during the day. It is a routine activity. Day no rest. Therefore they are exposed to the schistosomiasis day and night.  ***Interviewer:*** What are the predisposing factors for men specifically?  ***Respondent:*** Women play core roles in the households. They have to go process the fish, and selling it to the second buyer. Hence more contact than the man in the first place. Women by nature do housework e.g. fetching water, cooking, etc. This makes them being second in exposure.  ***Interviewer:*** What about the Pregnant women?  ***Respondent:*** Pregnant women are more vulnerable given their lowered immunity if their nutrition is not fine. All that mounts to vulnerability and contact with water.  ***Interviewer:*** And the Children?  ***Respondent****:* Children fetch water, males ones are taught fishing from an early stage. Boys begin as early as 10 years along the Nile. They go along with the older siblings or parents. They are taught how to do the fishing and others try to throw nets.  ***Interviewer:*** At this present time, how possible or realistic is it to prevent skin contact with high-risk schistosomiasis waters for each gender type? Give reasons for your answer?  ***Respondent:*** It is possible but a little hard for the males.  **For females** it is a little possible because they do not go into fishing activities. The Process has to be handled in two ways. One is dialogue with women to understand that being in contact with the water is exposing them to a certain risk. They have to understand that going into the river comes with risks. The mind has to be triggered to avoid exposure. Providing other sources of water may not bar women because there are other sources of water e.g. boreholes and tap water but there is a special attachment with the Nile. Some people have been saying that the Nile is very palatable water. Nile water is very tasty water. Although I should note Most of the boreholes are a little salty. So that alone is an issue that has to be dealt with. It is difficult to detach the person from the Nile.  **For the males,** it is possible but hard to achieve. Most are not in boots and are bare chested. Yet fishing is their way of life. Mass treatment of the men since each time they will be in touch with the water could help.  **The Pregnant females,** by being pregnant they are vulnerable. Contact with snails etc. is dangerous. She may also encounter accidents. Should have a special caption for the double risk it entails.  **Children:** Parents need to understand the risks of exposing the children to infection. Because as a child the parent takes full responsibility. They can teach them how to fish but if it is going to make them exposed then they should think twice.  Other activities e.g. mining of the snails – quick cash, quick money. Protection can be easily advised. They can be practiced by male and female in long sleeves, long heavy duty gum boots, especially since they are done on along the belt.  There has to be a little special caption for the pregnant women. Puts her on a double risk of having contact with the snails and other things during the process.  ***Interviewer:*** What are the nature of treatment seeking behavior with regard to Schistosomiasis  ***Respondent:*** It is not easy for all to seek attention when they get schistosomiasis. Many seek at an advanced stage when one gets pain. This is not good but the pregnant women, because of ANC gets a chance for deworming. There schistosomiasis may be one of the things to be checked for. They are exposed to messages to like you need to check for warms.  ***Interviewer:*** When women come for antenatal care, do they check for schistosomiasis?  ***Respondent:*** Not so but worms and deworming are part of the things asked for**.** Most men do not have this opportunity. Worms and deworming one of the packages.  Children’s HSB highly depends on parents. Most are fisher folk and hence are busy all the time. Parents and adults will determine for children.  ***Interviewer:*** What gender issues affect treatment seeking behavior of schisto patients?  ***Respondent:*** Women spend most times at home doing housework. She will have time to move to the facility more often compared to men. Most child rearing is for the women. Women need men’s backing for finance and support e.g. transport to the facility where far.  Men are mostly taken up in activities like fishing and digging. They tend to postpone until when it is a real problem when there is pain or when something is so crucial. For other health issues e.g. HIV men rely on the women’s test yet his status is different from that of the woman. This depends on the attitude that men have and chores involved in (digging, fishing and trade petty) until when it is a problem pronounced the man will tend not to care.  ***Interviewer:*** How does being of female or male gender or others (that’s is man; woman, mother/ father, pregnant mothers) influence behavior change and praziquantel uptake towards better control of schistosomiasis in your district.  *Behavior change:*  ***Respondent:*** Women more vulnerable but great at networking. Also easily take a U-turn change whenever advised. Men are always a little hard core. Masculinity makes them not take behavioral change easily.  *PZQ:*  Women are readily available at home, easily found and easily administered to. Men are away most of the time, already affected. PZQ is given directly observed. Will definitely not take it at the right time. Not home because he has gone to find food and money. Distribution DOS. Door to door, DOTS short course.  Parameters supposed to be assessed e.g. height are done by guesswork. Men are not always there to listen to the benefits and possible side effects. Hence get information from second party and bound to get information which is not up to date info.  Children uptake not affected because children fear medications especially when they are hungry. Children tend to fear taking medicines. Parents have to value PZQ, Children have to be readily available at home or at schools.  ***Interviewer:*** Can you please tell us about your experience in implementing interventions to control schistosomiasis in your community?  I have been doing it for 10 years. Working on environmental health.  **Good experiences:**  The drugs are for free of charge, at community level. They taken from door to door. If admin at schools easy to access them at that time. Effects of mass drug administration has led to a reduction of cases of distended abdomens, etc.  **Bad experiences:**  Other observations include e.g. side effects and serious ones because of high infestation of the warm. Local population are probably poor and hence may not have timely meals for the drug to work well. They shun taking drugs because they have not eaten anything. This deters one from taking the drugs in a timely manner. Community attitude about the drug – some just think PZQ drugs bring about a lot of disturbance because of the side effects mentioned earlier. Vomiting, pain in the stomach, dizziness, nausea and vomiting, diarrhea, loose stool, etc.  ***Interviewer:*** Praziquantel mass drug administration is one of the key interventions for treatment, control and prevention of schistosomiasis. Please tell us about  **Its accessibility:**  ***Respondent:*** There is shortage of the drug which affect coverage. We also use community drug distributors who highly expect to be paid, which has affected uptake. Facilitation as very little and where there are competing activities they get busy.  **Prevention:**  This is a little complex because it depends on one’s attitude. The threat is that it is not readily visible and it’s lower than for the treatment.  **Treatment:**  The Size of the drug affects convenient swallowing of the drug. Also the smell is considered very bad. It’s not same with prevention. Many have seen others vomit blood and die, extended stomachs, etc. so when it comes to treatment they are eager to take up treatment.  ***Interviewer:*** What would you do better?  ***Respondent:*** More of sensitization and community dialogue is paramount. With time they will pick up these messages. Talking to them and packaging proper messages for them to take. They are busy and won’t have the time for you. So you need too package information appropriately.  District has weak and collapsible soil. Latrines collapse a lot and it becomes expensive and tasking to put up another latrine. The community needs appropriate technical options in terms of latrines to encourage community members to have them.  More sensitization of use and maintenance is also required. Proper use is different from having it. The Rich have lined pits but even poor can be advised though it is expensive. Even those with lined pits they are submerged due to the rising water levels.  Have adequate stock of PZQ. Don’t sensitize and fail to deliver. Modify the size or improve. Fewer tablets instead of 5. More advocacy by the leaders to promote the drug uptake. Also monitor distribution and administration. Need to evaluate the nature of side effects, magnitude, prevalence, etc. Spell out all misconceptions of the drug.  **Interviewer:** Focusing on different gender (men vs. women vs. pregnant women, fathers, mothers, aunties, uncles, grandfathers, grandmothers, girls or boys)  (At work/ by occupation/ economy, in the family, in the health facility, or in political administration) help improve access and utilization of PZQ?  **Within the Family**  Design more appropriate ways to target the males. Women should be reached at the household. Implore the parents to support. Aspects of nutrition and uptake of the drug. Distribute in the afternoon, with lunch as a buffer. This varies with households and communities as people move to fish and to dig. Lunchtime not common for all.  **In your work place:**  Refreshers training. Gender issues relate to transportation, riding the motorbike to supervise. Females either learn driving or hire male rider. This affects monitoring or drug admin.  **In the health facility:**  Dependent on the individual community clients. Some prefer a specific HW, confides in that one. There are also others. Both gender should be empowered since it may not be possible to have enough. Empowered with proper means to distribute PZQ. Benefits should come first. Other products easily market. Identify HWs that are able to provide health services in the facilities. Refreshers should be taken seriously as the environmental health distributors. Routine HC services, not just mass distribution.  **In your community:**  Female VHT. Each village has two VHTs. One male and one female. So it gives them support for drug distribution Having one gender e.g. females is a problem as they all have domestic work.  **In government:**  Government should provide the medication in the facilities. Government and people have neglected the diseases. VHTs can be taught how best to administer the drugs and households  **At school:**  Some children absent themselves. We need to advocate and talk to them, Counsel telling them the drug is for their own good. No specific gender issues. Interventions there a little easy to achieve with staff trained on the program.  ***Interviewer:*** What changes in gender (roles, responsibilities, behaviors, expectations, or individual characteristics linked to a perceived sex identity) do you think can improve preventive chemotherapy or WASH in Pakwach?  ***Respondent****:* Proper identification of sub county supervisors to improve deployment to areas where they are most effective.  Pair the two genders to support each other’s when it comes to chemotherapy. One supervisor per SC is not efficient enough  WASH – dialogue with community members. Gender roles in wash can be played by both males and females e.g. putting water in hand washing facility can be done or refilled by males or children. Man can clean the container. This provides the next user a clean facility  WASH is limited to construction of the toilet, which is seen as the man’s role. Some FHHs have constructed these facilities.  Safe water shed are supposed to be well protected and maintained. They need to ensure they are well protected. Roles can be shared. Men can clean the water source.  Culture makes the man the HHH. So they need to be educated and sensitized to understand these are not being done to break their authority. Integrate both gender in roles thought to be for specific gender e.g. Cleaning the baby’s bottom, taking away the child’s feaces, etc. does not have to be a woman’s job.  ***Interviewer:*** Do you have any comments or suggestions?  ***Respondent:*** No  ***Interviewer:*** Alright Thank so much for sparing time for this interview |
