# Supplementary material for: Gendered lives, gendered Vulnerabilities: An intersectional gender analysis of exposure to and treatment of schistosomiasis in Pakwach district, Uganda
Source: PLoS Negl Trop Dis. 2023 Nov 10;17(11):e0010639. doi: 10.1371/journal.pntd.0010639 (PMC10684070; doi:10.1371/journal.pntd.0010639)
Supplement: S1 Data — (ZIP) [file pntd.0010639.s001.zip › KII Schisto Interviews/KII Ms. Hadijja Aliku.docx]

***Study title:*** Gender intersectionality

and

Schistosomiasis in rural Uganda

***Interviewer:*** *Nakiranda Salama*

***Respondent:*** *Ms. Aliku Hadijja* ***Position/Designation:*** *Clinical nurse, Pakwach Health Centre III*

***Proceedings;***

- *Interviewer welcomes Respondent*
- *Interviewer introduces herself*
- *Introduces the Project and Project Leads*
- *Introduces Funders*
- *Reminds Respondent of some crucial ethical considerations (Note: The Respondent had signed the consent form)*

***Grand Tour Question:***

*How does gender intersect with other factors towards influencing preventive chemotherapy and WASH interventions in Pakwach?*

**Interviewer:** Can you please tell us about yourself

***Respondent:*** My name is Aliku Hadijja. I am female aged 31 years. I am a clinical officer and I am the incharge of Panivero Health Center III in Pakwach District.

***Interviewer:*** What is your role in Schistosomiasis control and prevention?

***Respondent:*** Basically we do health promotion through sensitization, health education. We also do home visits to ensure that there sanitation, we also mentor the VHTs to ensure sanitation in their communities and also do health promotion in their villages.

***Interviewer:*** What policies guide his/her work?

***Respondent:*** We are under the allied Health promotions basically the public health and all that under the public health act.

***Interviewer:*** What policies guide his/her work in relation to Schisto?

***Respondent:*** Okay, am not so good with policies but all I know, it is all under the public health act.

***Interviewer:*** What are the key predisposing factors to schistosomiasis? Those that predispose the entire population in Pakwach.

***Respondent:*** Basically it is poor sanitation habits like open defecation that exposes people to schistosomiasis and also the people are a fishing community, they have access to water. They get into the water and that is the breeding ground. There are also those who are affected but not yet treated so they end up affecting everyone else in the community. Therefore the root cause is sanitation.

***Interviewer:*** What are the key predisposing factors to schistosomiasis for men specifically?

***Respondent:*** Usually, the men have access to water bodies, like lakes, river and all that, so that predisposes them even more. Also, this is a fishing community with men that are the ones who do the fishing, they urinate there, defecate and they still use the same water for drinking and bathing. So men are more exposed because they are more in the water than women.

***Interviewer:*** What exposes women to schistosomiasis?

***Respondent:*** For the women, they are always doing domestic shores, they also have access to water but also in the homestead they are responsible for sanitation in our rural setting so in the process of cleaning up going to the gardens, they get in contact with that open defecation. Usually they are doing farming work.

***Interviewer:*** Is everything the same with regard to pregnant woman?

***Respondent:*** No, for the pregnant women the special thing about them is that even when they have schistosomiasis, they cannot take praziquantel until they deliver. Therefore they stay with it and even continue spreading it. I think for them that is the problem.

***Interviewer:*** At this present time, how possible or realistic is it to prevent skin contact with high-risk schistosoma waters for each gender type? Give reasons for your answer

***Respondent:*** For the women it is access to clean water like those areas around landing sites if they give them piped water or if they can drill for them boreholes because most of them go to the rivers and the lakes not because they really want to but because they have no access to clean water. So by providing them with clean and safe water they would reduce or cut off going to water bodies.

Then for the men who go fishing and all that. I think the best thing would be treat the general population. If you treat everyone that means there will be no one to spread it. So even though people get access to the water it will not be invested because no one will be depositing in it.

Secondly, is also to promote proper sanitation and provision of toilet facilities each community should ensure that every community or landing site has latrines so that people do not urinate or defecate in the lakes and rivers.

**For men;**

For men since they mainly go fishing, it is a bit difficult to prevent them from getting into contact with the water since that is their source of livelihood they have been doing this for the long time so it is a process unless emphasis is put on farming and other sources of livelihood to ensure that they do not spend a lot of time in the water. They should make those other sources of livelihoods more productive and profitable that people make more profits than they are making at the landing site. This will encourage them to shift. The only problem is that the farming they are doing is not so productive. They are not educated on how to do the farming and all that. Whatever they produce is what they get they don’t get anything out of it. If the government can support them and they realize that can gain a lot from farming then they will leave the fishing.

***Interviewer:*** What about the pregnant women?

***Respondent:*** The pregnant women it is still accessibility to clean and safe water so that they don’t have to go to the river.

Then also sensitization of their male partners so that they can help them with fetching water and other activities during pregnancy

Here women are the ones in charge of homes most times and it might be tricky and difficult to make men help but me I would think that if they could make a drug that could be able to treat the pregnant women and the kids under five, it would be great. We are having a big challenge with that, we cannot treat young children under 5 and pregnant women yet we diagnose them with schistosomiasis.

***Interviewer:*** what is the nature of treatment seeking behavior with regard to Schistosomiasis?

***Respondent:*** About the behavior, I think the men usually come to the facility in the late stages. When they are sick unless when they come when the disease has worsened in the late stages of the disease and it becomes very difficult to treat them in our setting so when it comes to access to treatment men come at the stage which comes difficult to treat them in our setting.

For the women accessibility to health care is easy because they are responsible for the children and the children fall sick more often than that adults. So whenever they bring the children to the health facility they also end up getting access to the services, testing and being treated.

***Interviewer:*** What gender issues affect treatment seeking behavior of schisto patients?

***Respondent:*** With men, usually they base a lot in their economic activity for example he will see the time he will come into the facility to get tested treated as money lost. So he will only come to spend time in the facility when he is really sick and get do anything. If he just feeling a little sick, he will just buy pain killers from a pharmacy then keep doing what he is doing. They look at money as a priority to health.

**For Women**

Women usually come with children to the facility or for antenatal care when they are pregnant and in that way they also get chance to be tested for bilharzia.

**Interviewer:** Do they only come while bringing children or when pregnant? Don’t they come for their own seek?

***Respondent:*** It is the children that enable them to access health care or unless they are pregnant. It is rear to see them coming when they are not pregnant or when not bringing children. But at least when she brings the child she also talks of her complain, that’s why for women we diagnose them early than the men because they come more frequently to the facility than the men.

***Interviewer:*** How does being female or male gender or others (that’s is man; woman, mother/ father, pregnant mothers) influence behavior change and praziquantel uptake towards better control of schistosomiasis in your district.

***Respondent*** I think that when it comes to uptake in terms of treatment the community has seen what bilharzia can do. They have seen that it can take lives. If drugs were not expensive the parents would ensure that the children and everyone takes treatment and also prevention.

First of all a mother is always responsible for the children or even the father when he is admitted to the facility, she is the care taker so when it comes to the treatment of bilharzia they play a very big role. I think they are always the ones to alert the health facility.

For the fathers or men usually they come in to the picture in the severe form of the disease, they usually do not take charge.

With behavior change, when you look at things like sanitation, constructing latrines is a responsibility of the fathers in the homes, he is the one who digs the latrine and then the mothers do the cleaning and such sanitation habits children pick them from their mothers and the children are always with the mothers therefore they can influence them.

***Interviewer:*** Can you please tell us about your experience in implementing interventions to control schistosomiasis in your community?

***Respondent:*** Usually the things we do are sensitization because sensitization is key, the VHTs do home visits, home inspections and all that. Now when it come bilharzia you know that it starts from poor sanitation and sanitation starts from home so we go to home to emphasize that people do set up latrines and all that. But sometimes it’s taken up negatively. I think some community members are just in the process to understand that poor sanitation is the cause of bilharzia while others think that health practioners are just imposing their things on them. But controlling bilharzia is a process.

When it comes to awareness of health issues though sometimes people do not understand the importance of these things. We still have a challenge, we still have a lot of sensitization to do. Some villages have come up

Women are more aware of health issues that the men since they have more access. If you are calling for a community dialogue, 70% will be women and the men will be like 30% or even less. Men take their time. Actually women even play a very big role in behavior change in their homes.

***Interviewer:*** Praziquantel mass drug administration is one of the key interventions for treatment, control and prevention of schistosomiasis. Please comment about its access and how it has helped with prevention **and** treatment:

***Respondent:*** For me what I would say is that it is a good venture but the challenge is that the drugs are not always enough now like the last drug administration they were targeting only adolescents and school going children yet if we want to control bilharzia in the community, we have to treat everyone. Every age group is affected and every gender. The problem we have now is that we are treating a small group of people that makes it difficult to control the disease. So accessibility is limited and that is the biggest challenge we have.

Interviewer: How many times in a year is mass drug administration done?

We used to do mass administration twice but this year I think we did it once. And that one time was only for adolescents and school going children. In the facilities we do not have the drug so even when you diagnose someone you have to tell them to go buy and it is very expensive on the open market.

**Prevention and Control**

It has been able to help in prevention in the years where we used to do mass drug administration frequently, the cases would kinder drop. But if people are not treated, they will continue spreading it in the community and hence the numbers going up.

**Treatment**

For treatment, we can barely treat since we do not always have the drug in our facilities. Therefore when we diagnosis people we always tell them to go buy the medicine which normally they don’t because it is expensive. Last time we were suggesting that NMS puts it among the drugs that they supply to us but they were like it is not in their budget. So when someone is diagnosed today they have to wait until we do mass drug administration possibly the coming year.

***Interviewer:*** What would you do better (generally)?

I think we should lobby for more drugs so that we are able to treat everyone in the community because the more people we treat, the more we control the disease in the community and it will lower the number of new cases. Then we should also lobby for supply of these drugs in the health care facilities. We normally diagnose the same people.

***Interviewer:*** What would you do better focusing on different gender (men vs. women vs. pregnant women, fathers, mothers, aunties, uncles, grandfathers, grandmothers, girls or boys)

*(*At work/ by occupation/ economy, in the family, in the health facility, or in political administration) help improve access to and utilization of PZQ?

- **Within the Family**

In the family, I would put emphasis on the involvement of men because it is women who are always on these issues like sanitation, health care and all that. Sensitization should be actually focusing on the men most of the time. They should also participate in sanitation and health care.

- **In the health facility:**

Services provided at the health care facility should be able to attract the men. Like STD/STI services such as testing and counselling and then provision of contraception and all that.

**Interviewer:** Do STD services attract men?

**Respondent:** Yes

**Interviewer:** So, what would you do better focusing on women?

**Respondent:** Women are always at our facilities even for sensitization campaigns but we can improve by including schistosomiasis among the disease we sensitize them about.

**Interviewer:** What would you do better focusing on the community?

**Respondent:** In the community still we need to still emphasize on sensitization. For men we should not wait until they come to the facility but go to them.

From experience women respond faster to this issues but what they lack is clean and safe water and their partners are not supporting them.

**Interviewer:** What can government do better?

**Respondent:** Government should ensure accessibility to the drug and safe clean water

**Interviewer:** What changes in gender (roles, responsibilities, behaviors, expectations, or individual characteristics linked to a perceived sex identity) do you think can improve preventive chemotherapy or WASH in Pakwach?

**Respondent:** This might be very difficult to change because of the setting but I think the gender roles should not even be there. The roles should all be shared. If the males are involved they would see the importance of these.

Women if they were exposed to do other activities they could be able to put up like latrines.

**Interviewer:** Do you have any comments/recommendations/suggestions?

**Respondent:** I just want to emphasize that the focus should be on provision of safe water and drugs to the entire population and policy to promote partner involvement.

***Interviewer:*** Thank you very much for your time.
