# Supplementary material for: Gendered lives, gendered Vulnerabilities: An intersectional gender analysis of exposure to and treatment of schistosomiasis in Pakwach district, Uganda
Source: PLoS Negl Trop Dis. 2023 Nov 10;17(11):e0010639. doi: 10.1371/journal.pntd.0010639 (PMC10684070; doi:10.1371/journal.pntd.0010639)
Supplement: S1 Data — (ZIP) [file pntd.0010639.s001.zip › FGD Schisto Interviews/FGD MALE (18-45) YRS PANYIMUR..doc]

**GENDER INTERSECTIONALITY**

**AND**

**SCHISTOSOMIASIS IN RURAL UGANDA**

**TRANSCRIPTION FOR FOCUSED GROUP DISCUSSION.**

**Abbreviations and acronyms**

FGD – Focus Group Discussion

GP4 –Group Four.

F1-Facilitator 1

F2-Facilitator 2

Mod-Moderator

P1-Participant 1

P2-Participant 2

P3-Participant 3

P4-Participant 4

P5-Participant 5

LC-Local Council

VHTs-Village Health Teams

**GP4. MALE (18-45) FGD**

**F1:** Hmmm

**F1:** Thank you for coming. I think we can continue very well… we are going to give…..

**P1:** we are going to give....

**F1:** we are in group 4, age group (18-45 years). In front of us is a consent which shows that we have agreed to participate in this study; study with the main objective of finding out use of praziquantel (have you ever heard of praziquantel) “baya” (some people called it bitrooside), so we are looking at the use of quasi at bilharzia or at schistosomiasis hotspot of which Panyimur is one of them in relation to intersection of gender and other social stratifies. We are looking at gender. We earlier talked to the women, now, this age group we are talking to men only.

**F1:** Objective 2; to find out about bilharzia, schistosomiasis high risk water contact at schistosomiasis hotspot community in relation to gender and other social stratifies. So, that’s why you are here.

We are requesting Before we continue as group 4, we are going to introduce our self, including your village, make sure you pull down your mask so that you be louder, we are not going to remove this; it’s a recorder, which is recording some of the views you are giving which is confidential.

**F1;** you can tell us your name…..

**P1:** Am called Warom Isaac, from singular central, General Secretary LC1.

**P2:** Am called Jakisa Ronny from singular central south.

**P3:** Osaga Edison from singular b

**F1;** this one is still looking for his village.

**P4**: Katugo Rogers from mukindwa west

**P5:** Masendi Calvin Pola from mukindwa Main

**F1:** Main…… which main, they divided the village into two.

**F1**: We are all welcome. Before we continue, there is some small information about bilharzia that we haven’t talked about, we think we should talk. Bilharzia is real within us. Of which some people think it should be sorcery disturbing kids, I don’t know whether it’s with us…... Food poisoning, but, its within us of which in the whole Uganda 3 out of 10 people have bilharzia .Especially Pakwach, here in Panyimur they say more than 5 out of 10, 10 out of 10 have bilharzia. Relating to that, of recent they tested people. Pupils from Kivuje…. Actually children in community of Kivuje when checked for bilharzia 104, kids of 10-15 years, Out of 104 tested, 102 had bilharzia, and only 2 had no bilharzia. That’s in Kivuje.

**F1;** Secondly, community from Nyakagei, others from down Angumu, Villages surrounding Nyakagei. 108 Children went and all the 108 who went for checkup had bilharzia, none had no bilharzia. Meaning the problem is within us here in Panyimur. So, you find out of 10 people given tablets, at least 3 do not get healed, but 7 can get healed, so it means there is something about this disease and they want to find out what role men and women can play.

So, in our sitting here, we are going to ask around 12 questions among us and we are going to give our responses; the feedback will help health assistants and secondly the local community on how to live.

Before we go far, I am F1; Noah Okumu, there are some two people who will come in…

**F2:** Philip Oryema

**Mod:** Ocama Peter

The language we are going to use; which one is better. Can I ask you in English then when it becomes hard you can change to Alur? It is good like that….right! We are just translating for others.

When those other people are looking somewhere… we can begin.

So, questions… they are 12 questions, and we are going to go on each one of them one by one and kindly you will be responding one person at a time. Kindly extend the masks so that you can speak and be audible, secondly according to the ethics, speak the truth, per your experience, we shall have it confidential for the purpose of the studies.

**F1: So…First question; what activity do you or your family perform that might lead to the bilharzia infection?**

**P4:** Fishing

**P1:** Fetching water

**P3:** Defecating around the landing site

**P2:** Urinating in the source of the water

**P5:** Bathing

**F1:** what activity can you think of…?

**P4:** Digging along the riverbank; like “jogoo”

**F1:** “Jogoo”... Like wetlands… is it here also….

**P4:** yes…

**P4:** Rice plantation

**P5:** tomatoes farming along the Nile, they go there to fetch water from the lake.

**P1:** open defecation along the river bank…

**P4:** there is one…. snail mining

**P2:** constructing pit latrine along the Nile….

**F1:** how?

**P2:** when there is running water, the pit latrine can easily break, hence all the waste get into the water. Like now, there is rise in water level and where there was a latrine is now full of water, and that same water people come and fetch for home use, definitely it will cause bilharzia.

**F1**: serious……. Hihihihi...

**F1: all right, you guys are men so we are asking, why are men more likely to be infected than women in some communities with bilharzia?**

**P5:** when we look at men being actually at the epicenter of all these I mean, I can say that we are the bread winner at home. You as a man, you are married, you wake up very early in the morning and the woman asks for food, you must go and fish. That is one thing. So, we look for sources of living and how to earn living in our homes. That why I say that we are at the epicenter of all these. At the peak of getting infected.

**F1:** Why are men more likely to be infected than women in some communities with bilharzia?

**P1:** on my side is ee why we men get infected more easily than women is because most of the activities we do in the river are much more compared to women; like mining needs men. Digging needs men, fishing men, that’s why we are at high risk. Even bathing mostly men.

**F1:** on the other hand, why do you thing women and children are more likely to be infected more than men?

**P4:** on the side of children play so much along the river bank?

**P3:** like along the river bank, you find water logged areas are many, so children play in those dirty water.

**P3:** women do enter deeper in to the river looking for cleaner water, hence getting infected.

**F1:** so, children play in water and women like entering the dirty water as they look for clean water.

**P5:** ignorance among the children, they don’t know that the water is very dirty, So, they are ignorant of what they do.

**F1:** keep reminding us your name….

**P2:** Jakisa Ronny.

**F1:** ok, Ronny. I remember...

**P2:** women do their businesses like buying fish, washing the fish, preserving fish, taking their time along the river bank, removing the scales. That one also leads to high risk.

**P1:** to add on what he said, you know here along the river bank we depend on fish business, whereby fish business depends on struggle where you have to enter in water to get fish

**P3:** another thing is that to add on what he has said, we have a fishing mode called beach scening “korokota” which requires them to stand in water as they pull out the net because it’s the easiest fishing mode they can do, hence getting infected.

**F2:** that is the type of fishing which is much easier for them….

**P4:** yes…

**F2:** eehhhh

**F1: So…what changes in life style do you think can help you and your family prevent you from getting bilharzia?**

**P2:** provision of other sources of clean water

**P2:** repeat that question….

**F1:** I mean you or your family…. The lifestyle you or your family can make to prevent you from getting bilharzia.

**P4:** like for me, to prevent bilharzia, I can use clean water like borehole

**F1:** so, you should always go to safe water sources…

**P1:** we should avoid entering stagnant water and the children too. Purified water should be put under the sunlight for full one day to kill germs

**P5:** for us here, we don’t trust any source of water till it is boiled

**F2:** they don’t any water not until it is boiled….

**Mod**: that is at family level….

**P5:** for us we do deworming

**F1:** what other lifestyle...Isaac

**P1:** again in our village, the LC1 used to advice people to change the lifestyle to construct latrine 20-30 meters away from the water source to protect the community; like at our landing site there, most of the people were there. So, we appreciate the government, they brought the community latrine which we shifted it from down to up there so that the community should go there to avoid that because when they dig down there is a sandy soil when you dig the latrine 2 -3 months it will fall and all the waste there will enter water to avoid that we begged the government to bring us the Latrine so that all the community around there to go there and we employ some guard when they get you defecating outside they arrest you……

**F2:** What if they go there at night…

**P1**: we apply them at night, not day time. Day time we can see them hahahaha

**F1:** so, at night they are guarding the place ….

**P1:** yes….

**F1: so, what changes in your community or our health system or local government would help to control …Aah or eradicate bilharzia schistosomiasis in this community?**

**P3:** Edison… one of it is sensitizing…. Sensitization about the dangers of the bilharzia....

**F1:** so who should sensitize?

**P3:** the government.

**F1:** only…

**P3:** and us, it’s the community policy now

**F1:** ok .So the community and also the government should take sensitization as key.

**F1:** yes…

**P1:** Isaac. Under that if there is any sign or if you see any sign should rush to the health center for more treatment or checkup…

**F1:** ok……

**F1:** you think people should improve on their health seeking, anything small they should go to the health Centre to be examined?

**P1:** yes… that examination

**P5:** we have to make sure in the villages those people without the latrine, dustbin, and wire rail should be arrested or they forced them to dig pit latrine….

**F1:** so, the law enforcement should be through home visit and those who don’t have Latrines should be forced to dig one at least.

**F1:** our health system you are talking of government and the community, what about the health system. What do you say about…

**P4:** Am Rogers…. Like for the government and the medics, like ………they should bring medicine for bilharzia….

**F1;** ok… Ronny wants to say a word….

**P2:** Ronny…government should help us providing other sources like borehole, tap water…….. That one will help us.

**F1:** how?

**P2:** because people will no longer be going there to fetch water or wash their clothes from the river, they will just go fetch water from the toilet. Because there, there is no high risk of getting bilharzia…they don’t defecate there where there tap water and mostly those boreholes and tap they are within their homes.

**F1:** yes….

**P5:** on top of what he has said, for me am also trying to argue it out that …the government has tried their level best to bring this tap water, but there are certain homes and families that cannot connect to the water. So, it is also our negligence and a thing that can lead to infection. So what Iam saying the government has tried their level best to bring tap water but we cannot also afford because of poverty……. If there is a way that the government can make that water free for all the homes…..

**P4**: for supply….

**P5:** yes. It will be a little better. There by doing that we shall be trying or we shall try to curb down bilharzia…

**F1:** affording………

**P5**: yes we cannot afford…….

**F1:** Hmmm

**F1:** any other changes you think your community can have… if not Aah. This is more of practical. Has your family ever discussed the use of praziquantel drug……..or bitrocide.

**P4:** Ambila…

**F1:** Ambila

**F1: have they ever discussed used of that drug or any way to prevent schistosomiasis?**

**P1:** my family has ever discussed because I remember one time when I suffered from diarrhea for almost one week, but it was not a bloody type that you may suspect it to be bilharzia. I tried all the medicine in vain; I could not go for stool checkup because all the medicines were not working. The best thing that they put me on was this “baya” thing you are talking about. Bit when I tried “baya” from that time up to now. I have never suffered from diarrhea again… probably it was bilharzia that......

**P3:** (laughs…..hahahahahahaha

**Admin:** or may be “baya”……

**P1:** or may be “baya” chased the entire diarrhea…

**F1:** hmmm

**P1:** yes.

**F1:** so when u discussed as a family… what was their opinion?

**P1:** the opinion is that whenever the government is giving out this…. we try all our best that we should at least get and take…

**F1:** it seems people are positive about it….

**P1:** yes…people are also positive about it because they have seen the benefit with me… and there are some other people that have seen the benefit because as you said our main economic activity here is just fishing, so, people have seen it positive and they have taken it on the positive note.

**F1:** who again….

**P3:** aah… Edison. You see “baya”, me… I took bias… among children when at primary school they could follow name in alphabetical order…. But my name is Osaga Edison, and I was the first. After they could follow other pupils. So, and by that time bilharzia was disturbing me, my stomach had swollen, even my skin was not looking good, and me myself was not looking good. My stool had some blood, when passing out stool, I could feel pain. But, after I used “baya”. “Baya” helped me a lot. As I speak now at home “baya” doesn’t get finished.

**F1:** even now you have….

**P3:** yes….

**F1:** where did you get it from?

**P3:** even for sale, they can buy….

**F1:** ok…. Hahahaa

**F1:** so, if you are talking of bias at your home. Do people take it positively?

**P3:** yes…they take it positively.

**F1:** so u talk about it every time whether the drug is there or not and about the prevention.

**P3**: yes….

**F1:** and you, you have raised your hand.

**P2:** we have discussed with my friends, community, am talking about the effect of the drug. Wen you swallow this drug it has much effect, it cause itches on your body, stomach aching and many other things….sometimes it can even

Make you lose your appetite. We discuss it a lot. If there could be some changes on the, those are what we discussed with….

**F1:** so, some people think… what do you think...what do you people think when you discussed?

**P2:** we think if u can bring this “baya” which cannot have much effect on our body. It will be better.

**F1:** you may not accept the “baya” easily….

**P2:** of course… (Laughs hahahahaha, hahahah

**P4:** that is the effect

**F1:** so you take it with reservation… on condition….

**P2:** condition… Hihihihi… you see.

**Mod:** you swallow after meal….or….

**P2:** eehhhh….. It still disturbs….

**P1:** I have also experienced it.

**Mod:** yes… Isaac.

**P1:** to add on to that at first before we entered into the LC system to discuss. my Mzee was like the group which was for “saitel”… when we were still young, he could discuss the same experience Ronny put across was the same experience we could put before him… that “baya” of these days are not like for other days.. And he could say yes. Not like those days… so, he started putting it before us to discuss and he could make sure that we are the first to take the “baya”. But, as we swallow, we could have divided minds…But when we started getting used to it. He said they just get used to it. So, the encouragement he was giving us made us to agree that when unswallow the “baya” and the diseases get killed, next time when you swallow it doesn’t disturb you, and its true because at first it disturb us, second still but, the third time it did not. And he started encouraging us that now we are no longer have bilharzia in our stomach. That’s why as we speak now, our family have accepted the “baya”… because of that. But, it disturbs people only that it gives encouragement that if u swallow today , tomorrow u swallow and when the diseases are killed, it will not disturb again…… when it kill the diseases in you. Hahaha

**F1:** ok… aahh

**F1: the next issue is the… some people are still a key among us at home... who is most important in deciding if the family member come in contact with bilharzia affected water or receive praziquantel for treatment… who decides on the treatment at home?**

**F1:** …Rogers

**P4**: the mother.

**F1:** you are saying the mother…. Why?

**P4:** because she is in charge of water and other things.

**F1:** eehhhh…. Minister of water and evaluation…..

**P4, P1:** hahhhahaha

**P5:** for me am saying generally parents. When you talk about parents, I mean the mother and the father .They decide everything. For example…when they are giving out baya. They are the one to decide that…. let us all go because this thing is according to VHT giving to per village. So, it is him now to decide that we are all taking. Then, it is also the mother can decide that may be lets go and fetch this kind of water and we go there. The mother can say that tap water is very hard, let’s go fetch water from the lake for washing our clothes or utensils. Generally, it is the parents who decide…

**F1:** yes…aahh... Isaac

**P1:** Isaac… on my side, mostly am supporting sometime the parents and some parents they don’t care. So, it’s someone who supports the issue of the family. Like our home is me myself. It follows the knowledge someone has in the family.

**F1:** who are you in the family?

**P1:** ….. In the family… am the first born.

**F1:** ok…

**P1:** and I tried that, sometimes when someone is sick and you go to the parents and they keep on saying we shall see, we shall see… when the sickness is continuing. So, I take the role as the first born because am knowledgeable. So, for me I think whoever has the knowledge in the family can take the role for that.

**F1:** but, now let’s say u have the knowledge but before the father decides whether to be given treatment, can you decide?

**P1:** aahh… what u are saying happened. I said they take the kid to the hospital and he said that was his kid. I said, when the kid dies… that will be our kid who has died. Then we forced and we took the kid to the hospital, I took the kid myself. After when the kid got better and we came back from the hospital… he said to me…Good work. He resisted because of lack of knowledge he had by that time... because he was a drunkard and ignorance was too much with him. So, we re-enforced. So, I think whoever has the knowledge and power to help in the issue concerning the bilharzia can help the family… not only the parents…

**F1:** don’t you think your position in the local council executive influenced that decision?

**P1:** LC… it cannot affect... Hahahahah hahahahah

**F1:** Edison….

**P3**: for me am just adding because I have seen in our home about the “baya” the drugs for bilharzia. Parents, ok. My parents not parents could collect everything, then when all of us are around they give us to swallow. Coz, they know some children like “baya” can throw the medicine away. Like am giving easy example like my elder sister Sunday could fear the drugs and throw, so when the time for medicine he could give by himself. Like swallow then go, swallow then go. As he sees. So, issue to do with water around home is the mother because, when the water is not there, the mother goes around looking for it.

**F1:** ok….

**F1: who should be given these drugs (praziquantel)?**

**F1:** Edison…

**P3**: those who have bilharzia

**F1:** eehhhh. Those who have bilharzia should take…

**F1:** then …Rogers.

**P4:** those who have been tested and found to have bilharzia

**F1:** those who have bilharzia and then specifically those who have been tested…

**P1, P5;** Hahaha hahahah

**F1:** Pola…

**P5:** for me I feel everybody should take, because I have never seen bilharzia test being carried. I have never seen, because bilharzia can be manifested when someone is defecating, actually diarrhea, someone is having flamboyant stomach; the stomach is swollen…..

**P2**: flamboyant……Hahaha

**P5:** that is the thing I feel…because.

**P4:** the signs……

**P5:** that is the sign I think. So, generally I feel everyone should take baya.

**F1:** ok… Ronny

**P2:** for me I feel everyone should take but it should not be in a forcing way because some people may not wish to take the drug because they are not always in access to the landing site.it should be freely. They should be free, they should not be forced.

**F1:** do they force….

**P4, P2:** Sometimes… it is there, Hahaha Hahaha

**F1**: that is in the family….

**P2:** even at school, they can also force you.

**F1:** Isaac…

**P1:** and me also I am supporting that. Everyone. Like in the morning you told us that you can take “baya” now and you will know that you are free not knowing that you have got it again.. That is a risk. When time comes for “baya”. Everyone should take whether it is there or not, it will get the thing there.

**F1: who do you think should not take “baya” according to you?**

**P2:** only babies…

**F1:** babies…. How old…

**P2:** that is from 5 years and below…

**P4:** Hahaha…. 17 years below

**P4, P5:** hahahahahahaha

**F1:** ok…… so what are the sizes

**F1:** Ronny said 5 years and below… according to his opinion…but why?

**P2:** this ones can be stopped from going there… but, these ones from 7 years and above… eehhhh. They can even skip around with friends and they go there…

**F1:** but, are u aware even children at 2 years even have bilharzia….

**P2:** 2 years………

**P4:** even a year…….

**P2:** at first child who start crawling… the crawling kid is at a risk of getting bilharzia…. This “baya” is so powerful, can you imagine adults are even aah…….

**P2, P4:** hahahahaha

**P5:** for me am saying someone who is not supposed to take bilharzia is a pregnant woman, a woman who is pregnant should not take bilharzia because we have seen the effect because it can cause what…..

**P4:** not bilharzia… but “baya”…

**P5:** no mean am sorry…..

**P5**: they should not take “baya” because I have seen there was a case of a woman forced herself to take and it almost caused what…..

**P4**: abortion

**P5:** no… not abortion

P4: miscarriage…..

**P5:** miscarriage……….almost caused miscarriage… so, to me a woman who is pregnant should not take but the baby can take. Except. Yes because we said that water we fetched from the lake can cause bilharzia, a baby can be bathed with that water which is fetched from there. so, in the process of bathing, the bay can contract bilharzia… so, it is only the woman who is pregnant should not take except for babies ,the dose can be specified…. Thank you.

**F1:** any other person u think should not take…

**F1:** Rogers.

**P4:** Eehhhh

**F1**: who do you think should not take?

**F1:** yes… Isaac

**P1:** The sick people are not eligible to take…..

**F1:** The sick people....Ok…. Wow!

**F1: ok….. Are there reasons or any reason why you or your family members or community should take... is there any reason you would start and say for me I have to take this drug…. My community has to take this drug….. ….is there any reason?**

**P4:** yeah….

**F1:** what is the reason?

**P4:** like for good health

**F1:** so. For good health… like which good health?

**P4:** like being free from bilharzia…

**F1:** Isaac………

**P1:** for me only one reason that all family should swallow baya or take baya so that it fight bilharzia … its one, but, the access is for the condition to reach like that kid we are seeing there will cost a lot of money… and the reason is that before it increases or cost a lot of money we should treat it first …..

**F1:** we should prevent it before

**P4:** yes… yes…

**F1**: that is his reasoning…..

**P4**: hahahahaha

**F1**: is there any other reason? Ronny

**P2:** eehhhh

**F1:** for the effect of the first one, if you can change another one which cannot bring that effect. Any reason that can make you swallow?

**P2:** reason…. The reason is there…

**F1:** which reason?

**P2:** I don’t know whether it is with me or not, because, testing is not being done. If they found that bilharzia is on the ground and the drugs are there I should take to be healthy…not to wait when my stomach is big like that one…. So that I swallow, I have to take it.

**P4, P1:** hahahaaha

**P2:** they say prevention is better than cure; the only principle is that…….

**P4, P5:** hahahahah…. So it is hard to know. Whether me or you.

**F1:** its good… ok.

**F1:** are there reasons the person should not take or not be given praziquantel?

**F1:** yes. Pola

**P5:** one of the reason we said when someone is pregnant because, it can cause miscarriage that is one reason. Then the second reason may be the side effect to me, because you know this medicine react different with different individual…like some people, especially me when I take Chloroquine it disturbs me a lot. So, it varies from one person to another… so, probably that could be one of the reasons when someone has that side effect which may not be a genuine reason anyway….. But, to me, I feel that should be the reason.

**F1:** so that side effect, could it be that of person’s former experience? Or…..

**P5:** just like... Let me say former experience can also cause that. Like for me the first time I took “baya”, I could not sleep…. for the one week, I don’t know whether it had got bilharzia with me or what... I could not sleep. The second time when I was coming to take… I took with a lot of fear but it did not give any side effect to me again… the third one I took , it had no effect as I talk right now so, it can also be because of the first experience that someone may fear to take it.

**P2:** usually, like other people usually may take it today, another day the same… so it will give a fear to take the thing again.

**F2:** someone with known history of reaction…. Hahaha

**F1:** with known history of reactions you will say aahh. This one…….

**F1;** ok….so far so good……

**F1: now…..access to these drugs like antimalarial drugs, like praziquantel Might be a problem… access…..**

**F2:** is that the case here…

**P4:** yes…. yeah….

**F1:** is access to these drugs a problem?

**P3:** no…. it is not a problem…..

**F1:** where do you get it from?

**P1:** no. It is not a problem because we always wait for them from the government to supply and then we go and take….

**P1:** it is the government to supply to us….

**P4:** hahahaha

**P2:** you cannot get the bilharzia at clinic. No, this... I mean we cannot get “baya” from clinic

**F1:** so, access to it is……

**P2:** It is there… not hard. The timing are there…. They give exception….……

**P1;** it is there.

**P4:** it is vice versa

**P2:** Because, it is expensive….

**P1:** it is not expensive… these things are given for free

**F1:** ok. Let’s discuss one at a time.

**P5:** the access of getting this is that you know government has its program to give the thing. A certain when government wants to bring “baya” you find another thing enters there. Like Ebola comes, all the….. Government will turn to Ebola leaving the access of this “baya” alone. That will give us the access that to get the “baya” for us is not easy… and if you get it in clinic, the price is high.

**P2:** hihihihihhi

**F1:** Pola, Pola first tell us…. Right now….right now….

**P5:** No.Am saying this… it is not easy for us to get it but very easy for the government to give us… are we together. When the government give it out, we get it.

**F1:** so, this access is now for u…. if u need the medication now, for yourself or for your family…..can you get it…

**P5:** we can get it when I go to the health center…it is there.

**F1:** can you be given…

**P5:** yes…. you can be given

**P4:** Are you sure?

**P3:** in most cases, we go there and we don’t find them.

**P3, P1:** hahahahaha…..

**P5:** anyway, am talking to my opinion…..

**P1, P2:** hahahaha… may be you have your people there working.

**P2**: hahahahahaha

**P5:** you are talking about all the medicines, you are not talking about “baya”….

**P5:** hahahahahaha…………..

**F1:** we just gave an example like antimalarial, like coartem, at all time do u go there and buy it? Antimalarial.

**P5**: it is there…

**P4:** which sometimes…..

**P5:** this time it has been given out to the VHTs…

**F1:** that is for children…below…..

**P5:** not even children... Hahahahah

**P4:** it’s for children…

**P5:** they give us the one for children…. Hahahaha

**P4:** No. May be you have access.

**P5:** You people, I don’t stay here…. I don’t have access.

**P3:** hahahahah….. May be he is related to someone there at the health center…

**F1:** ok… we gave example like antimalarial and praziquantel. Are they accessible or is it a problem… specifically, let’s talk about praziquantel it accessible any time.

**F1:** yes….

**P3:** it is not easy at all, for me I have been looking for that medicine for long… even now like have two remaining, we need more, was it yesterday I was asking Jessica how are you and she told me it was not there.

**F1:** for you, how did you come to get those two?

**P3**: it was the one for…. Oba. It was given last year…

**Mod:** eehhhh…., Mzee is still there. Mzee is still continuing with his work for the community. So, he keeps some there…

**P3:** yes.

**F1;** for the family or for the whole community

**P3**: for the family.

**P1, P2:** (laughs …Hahaha

**Mod:** And now if may be you don’t have it in the shops, so do you buy them… do you easily get them

**P2:** no, not easy to access

**P2:** even if in the clinic, some people fail to reach there… and ask for “baya”. If they get it, it is expensive.You may find it at 2,000= of which some people are failing to buy mere Piriton of 100= is hard.

**P5, P4:** hihihihihi

**Mod:** ok,this is for us

**F1: Do you think being a man would make a difference in you or your family accessing praziquantel or using praziquantel?**

**P4, P5:** hihihhihihi

**Mod:** yes…. Isaac

**P1:** yes, it brings the difference in our family… like now I know many people like you.

**Mod:** social network.

**P1:** yes…

**F2:** so, men have more social network…

**P1:** if anything needed at home, Women don’t move, for me I move…. like now am in this meeting, I will know more information. I may know other places for “baya”

**Mod:** hehehehehehe…… this man is still advocating for more “baya”…

**Mod:** Ronny… what do you think... You being a man can make a difference?

**P2:** it can make a difference…am just advising them that by telling them what they can stop them from acquiring this bilharzia… like in our family if there is a fisherman I advise him to wear a gumboots if he is going along the landing sites. Accessing drugs within the community….

**P2, P5:** first repeat the question.

**F2:** do you think being a man would make a difference in you or your family in accessing the drugs or using the drugs.

**P2:** it is not hard to access the drugs, because I have no source to access the drugs….….

**P5:** is being a man or because you don’t have the money…

**P5:** hihihihihi

**Mod:** that is just general one, where everyone cannot access but, if may be it was there, you being a man would you have advantage over the rest?

**P2**: advantage over the rest, I would have

**Mod:** how?

**P2:** if there is need for “baya”… I would go and buy it.

**F2:** so, the difference is, for you, you have the ability to generate some revenue to go buy those drugs and then, what do you think of women…is it hard for them or it is easier?

**P2:** it is hard.

**F1**: Why?

**P2:** because. Anyway some women are also like me which is accessible, but some are also not accessible. Because others are scared to reach some of these clinics, they may also have money but, they may think “baya” come during government program. They don’t know where to acquire them from… those are the things.

**F2:** lack of awareness…

**P2:** yes. Hihihihi.

**F2:** so you think men have more information than women which can make them easily these drugs.

P2: yeah... Mostly men, because women are now being tied up there at homes…

F1: Pola… do you want to say something.

**F1: so, the question is do you think being a man or a woman would make a difference in you or your family in accessing praziquantel or using it?**

**P5:** for me, I would say yes. In a way that as a man there are some decisions which are final. For example, When these medicines are given to child, there are some children who tend not to fear their mother, but you as a father when you say, I want this done, these children tend to listen to what you say that is why am saying being a man would make using the medicine easier because the decision we take children tend to follow more than the one of the woman..

**F1:** yes… Edison.

**P3:** another thing is, as for me as a man, connecting to get the medicine is easier. In a way that there are VHTs who do not reach some families, because, they have changed now the bilharzia drugs first reach VHTs and they distribute of which they don’t reach some families….so as a man, as I move, women remain home.. I can get the information, I go to VHTs and talk to them. Because, women are tied up at homes.

**F2:** so, as a man u can easily, first of all easily access the information and secondly you can easily reach out were the medicine is and make sure the family is. Especially at the village setting.

**Admin:** and then what happen to the woman when she is trying to access the drugs.

**P3**: no. They are tied up at homes. So you find they cannot, the women.

**Mod:** speak in English so that she can get you well… hehehehe

**P3:** these women at the shores they don’t go the LCs, VHTs to go acquire the information going around. So, they wait till they reach their homestead so, like me, a man who moves around I keep on visiting them like “Kadoka” can come and tell me...eehhhh. Eddy they are giving out bilharzia drugs you go to your VHTs and get. So, those women are tied up at homes…

**Admin;** and you Rogers. What are you saying?

**P4:** I agree with him Hihihihi.

**F1:** yes…. Isaac.

**P1:** to add on to that, as a man, women are short tempered in this way…may be she will go to the VHTs, getting VHTs very busy little bit, she will say ohh…kumbe these people their things are for some people… they involve some… that one, short tempered of that woman there will make that woman fail to access the drugs.. As a man, I will go there and I will not get tired, because, when a woman comes home. Another work, tied up as another friend has said it may not make it easy to access. But, as a man, I will follow the procedures of a man…

**Admin:** let us first say. The VHTs are they women or…

**P1:** no. They are mixed up.

**F2:** the women……..

**P1:** our women here are short tempered, you know that VHTs have their own work. They may say u wait, or come in the evening or kumbe in the evening I have also my work, kumbe that drug is for some people. You know the language the women use. .. Hehehehe…. They start exchanging…. They start exchanging even though the VHTs say they may be the thing is like ours. Am here for the community, we have one down there, one is up there. When you go there I don’t have it, let me go and check up there… then she will start saying ok… ours when we come they say things are not there but, for others are being given. That one is short tempered… the woman will not come back again… for us we men are patient, we try to wait that make us easy to access the part of the drugs.

**Admin:** you are patient or u just having it…

**P1:** hehehe… no… we are patient.

**P3:** again also. There is also tribalism…

**F1:** tribalism…. How does it link to gender?

**P3:** You see these Families we are having in our town here. We are having very many different clans around. So, sometimes you find some families are crying that information are not reaching them, aahh. Sometimes when these VHTs are registering, they only register some few people around LC communities and leave others. So, these are some of the….

**F2:** as a man….

**F1:** like a general challenge…**F2:** that is another reason.

**F2**: for you as a man to be denied the service or a woman to be denied or have an advantage to get the service, is it related to you being a man or not….

**F2**: That one is clan. That’s ok. This question was referring to your position as a man…

**P3:** ok….

**F1:** otherwise, I think that was our last question, Aahh.I don’t know whether you have other questions apart from these ones, but, hehehehe, I say thank you for your time and responses you have given. In case you have any concern, you can raise it, it’s time.
