# Supplementary material for: Gendered lives, gendered Vulnerabilities: An intersectional gender analysis of exposure to and treatment of schistosomiasis in Pakwach district, Uganda
Source: PLoS Negl Trop Dis. 2023 Nov 10;17(11):e0010639. doi: 10.1371/journal.pntd.0010639 (PMC10684070; doi:10.1371/journal.pntd.0010639)
Supplement: Attachment — Submitted filename: Manuscript Response Sheet.docx [file pntd.0010639.s002.docx]

**Manuscript Response Sheet:**


**Reviewer #1: Line 107- missing text after education.**

**Response:** Education deleted.

Line 115-118- it would be good to articulate the provision of PZQ in relation to mass drug administration campaigns as well as considering its availability within the open market (although the points about the open market are valid).

**Response:** We have revised the phrase to indicate that prazaquantel was available on the essential drugs list of the WHO and Uganda Ministry of Health but still many did not access it for various reasons including not being offered and being absent.

**Line 148-150- can you clarify what you mean by the long responses….**

**Response:**

“Long” removed since the responses were qualitative to enable detailed discussion, which is stated at the beginning of the paragraph (Line 148).

Description of gender, with sex then used as the purposive selection description- either change to sex in terms of participant identification or move toward terms men and women throughout the manuscript.

**Response:**

Gender changed to sex in the methods section.

**Data analysis section- make a link here to the type of intersectional analysis applied and perhaps a few reflections on the process- as per comment above based on theoretical section.**

**Response:**

This has been addressed throughout the article but prominently in the discussion.

**Reviewer #2: Introduction
Line 53 – would be good to state the year of the prevalence of 25.6%.**

**Response:**

Year included, as well as the study that generated it. (See Line 53-54)

**Methods:
Line 113 – what do you mean by customary beliefs limited the use of the water source?**

**Response:**

**This has been removed, focusing on the actual data without interpretation till the discussion section.

Lines 113-115 – this is not clear to me about the fuel / wood. Can you please revise?**

**Response:**

Fuel/wood replaced with cooking fuel to boil the water

**Lines 116  PZQ is on the WHO Essential Drugs list.** See <https://cdn.who.int/media/docs/default-source/essential-medicines/2021-eml-expert-committee/expert-reviews/i1_alb-meb-pzq_rev1.pdf?sfvrsn=91190dbf_5>

Just because it is on the Essential Drugs list, doesn’t mean that it is available at the PHCs. Suggest rephrasing this sentence to state that although it is on the essential drugs list, it may not be available for routine treatment at the PHC outside the MDA. (If that is indeed what you are experiencing.)

**Response:**

This section has been re-written to remove any insinuation of PZQ not being on the essential drugs list. Instead the point has been made to emphasise challenges in access (See Line 136-145)

**In the study population section – you describe the study population as rural – but above authors say that the sites are urban and peri-urban. Please clarify.**

**Response:**

The population was urban and peri-urban. I have indicated as such. The rural was in reference to the whole district but I have removed this phrase to avoid confusing the reader. (See Line 150-151)

**Data analysis – did you use any qualitative software?
Response:**

We did not use any qualitative software. We analysed manually. I have clarified that in Line 194.
--------------------

**Results**
-Does the analysis presented match the analysis plan?</br>
-Are the results clearly and completely presented?</br>
-Are the figures (Tables, Images) of sufficient quality for clarity?</br></br>

**Reviewer #1: The results section** is very interesting and there are some new and novel findings that are currently absent within the broader NTD literature presented. However, the balance of quotes to text and length of quotes makes the readability of the results very difficult to follow- could the authors select key quotes to include in the text and add others to a summary table or supplementary file- this would also prevent the reader from completing there own analysis of the research as is currently the case.

**Response:**

This has been addressed by reducing the quotes to allow only the key ones. The results section has also been re-written to separate the results from the discussion.
 **Reviewer #2: Results:
Would be nice to provide a summary of all data collected at the beginning of the section**

**Response:**

The results section has also been re-written to separate the results from the discussion.


**Lines 230-231 –** I am not sure that you can say young men are more culpable for transmission based on one quote. Rather that young men may have more freedom to move around, including defecation openly at night as there would be less social and government controls on their behaviours.

**Response:**

This sentence has been expunged.


**Line 239-240 – do you need a reference for the definition of gender roles?**

**Response:**

The reference has been provided.

**Line 247 – consider adding some specificity to the comment – on men’s role e.g., to ‘financially’ provide as opposed to only ‘provide’**

**Response:**

‘Financially’ provide has been expunged.

**Lines 304-355 – is there anyway to reduce the length or number of these quotes?**

**Response:**

This has been undertaken.

**Lines 361 – lifting their clothes in the water will not necessarily expose them more to schistosomiasis infection. Once they have entered the water, they are exposed.**

**Response:**

The quote has been expunged

**Line 363 – authors have assumed that men go into the water 1x week, yet in the next section we hear from the men themselves that they are frequently in contact with the water. Please consider revising this statement.**

**Response:**

The reference has been provided.

**Line 453 – where is the evidence that men / boys are exposed earlier than girls?** Girls will be washed in the river, will fetch water at early ages as well. This statement does not match with the evidence presented in the paper. The community perception may be that men / boys are more exposed due to the length of time they are in the water, or the fact that they don’t have a shirt on. But this is not scientifically valid.

**Response:**

The assertion that boys were exposed earlier has been expunged. However it is referred to in the Discussion alongside that of girls, showing they were equally vulnerable through data on gendered pathways was missing.

**Lines 583-5 – I am not sure that you can say that men are more likely to be infected than women. Both sexes have high exposure to contaminated water.**

**Response:**

You are right. The quote has been expunged since we did not collect actual data on infection rates.

**Lines 619 – Table 1 - did the interviewers ask if men and women were equally at risk?**

**Response:**

The interviewers asked who they considered was more at risk. Even then, the responses are opinions and cannot be relied upon to determine for sure who was most at risk. We only discuss the opinions and why they arose.

**Line 633 – is a reference needed for the definition of treatment seeking behaviour?**

**Response:**

The reference has been provided.

**Line 663 – PZQ is on the essential drugs list as noted above. The government however may choose not to purchase it for use in the PHCs.**

**Response:**

This has been addressed. PZQ was on the list but suffered insufficient and unpredictable supply, which has been pointed out severally where this issue arose.

**Lines 658-669 – are these comments directly related to the qualitative research?**

**Response:**

Yes they refer to the research but are synthesized with findings from elsewhere.

**Lines 713-725 – how is PZQ distributed? To the community or only to school aged children?**

**Response:**

Explanation provided that PZQ mass drug administration was provided to school going children of targeted schools and households within the community.

**Lines 842. Should be neglected tropical diseases which is more commonly used than neglected communicable diseases**

**Response:**

This is noted and addressed.

**Conclusions**
-Are the conclusions supported by the data presented?
-Are the limitations of analysis clearly described?
-Do the authors discuss how these data can be helpful to advance our understanding of the topic under study?
-Is public health relevance addressed?

**Reviewer #1: The limitations of the analysis are presented, however the situating of the findings within the broader NTD literature is absent- there is only one reference within the discussion and conclusions section.** Would it be feasible for the authors to restructure the latter sections of the manuscript to streamline the results as described above and then have a separate discussion, recommendations and conclusion section. The discussion should link the findings to the broader NTD literature, in particular that which focuses on NTDs, gender, and intersectional analysis. For example see the special issue on gender and NTDs within this journal.

**Response:**

This has been addressed.

**Reviewer #2: In general the policy implications reflect the data presented, but they could be written in a more succinct manner. Some specific comments below:

Policy implication:**
a)      Is not clear and needs to be written more succinctly. Is the focus on pregnant women? Fear of adverse events?
b)      Schistosomiasis is not slated for eradication as per WHO recommendations. But for elimination.
h)  this may be an unrealistic recommendation
i) this shift was demonstrated in a paper on LF MDA in Indonesia (Krentel and Wellings)

The authors do not situate their findings within the broader literature on gender and infectious diseases or gender and NTDs. This is needed before moving into the policy implications and would further bolster these policy recommendations.

**Response:**

Addressed to situate the research in wider NTD data.

**Editorial and Data Presentation Modifications?**

**Reviewer #1: (No Response)

Reviewer #2: Editing notes:**

Please have consistency on the capitalisation of schistosomiasis.

**Response:**

Except at the beginning of sentences schistosomiasis is written with a small ‘s’.

Line 102 – spelling – should be carved I think?

**Response:**

Curved corrected to carved

Line 107 – rest of sentence seems to be missing?
Line 137 – suggest another word – not ‘subjected’ – it sounds like a punishment
Line 271-277 – reads awkwardly – please consider revising
Line 292 – health care worker (needs an -er)
LLine 740 – swam not swarm

**Response:**

All editorials handled.

**Summary and General Comments**
Use  this section to provide overall comments, discuss strengths/weaknesses of the study, novelty, significance, general execution and scholarship. You may also include additional comments for the author, including concerns about dual publication, research ethics, or publication ethics. If requesting major revision, please articulate the new experiments that are needed.

**Reviewer #1: This is a really interesting paper and the analysis and application of gender theory is new and insightful.** However, the limited link back to the NTD literature presents a slightly wasted opportunity in being able to make recommendations for action that could shape improvements in NTD care from a gendered perspective. I would encourage the authors to make these links more strongly as outlined in my comments above to maximise the impact of this work.

**Further comments on the introductory section as follows:**

Line 40- the before world health organisation
Line 56-58- the list of NTDs affecting Uganda doesn’t seem exhaustive- perhaps the authors need to check against the full list of NTDs. For example, things like snakebite should be included.
Line 60-63- the description of schistosomiasis control initiatives could be more streamlined- could the points about mass drug administration all sit together in the sentence prior to the alternative intervention approaches.
Line 71-74- good text – perhaps better suited to methods section.
The section on intersectional gender analysis is clear, however it would be good to include a couple of sentences on intersectional theory as well as the extensive description of gender analysis. For example, many would expect gender to be part of intersectional analysis implicitly- perhaps be firmer in articulating that gender is a key entry point and that you acknowledge the inherent need to consider gender in an intersectional analysis. Perhaps also describe more about your intersectional standpoint e.g. non-additive, intercategorical etc. See Christensen and Jensen 2012.

**Response:**

All the above edits had been addressed. Christensen and Jensen 2012 referenced and explanation improved.

**Reviewer #2: General comments -**
An interesting paper with useful results and exploration of how gender roles predispose men / women differently to infection and to treatment seeking.

In some parts, authors should note sex not gender.

**Response:**

Addressed.

**Reduce the length of the paper – the quotes are long and repetitive. Is it possible to include some in an appendix or supplementary table?**
**Response:**

Addressed. Several quotes removed.


**Figure Files:**
While revising your submission, please upload your figure files to the Preflight Analysis and Conversion Engine (PACE) digital diagnostic tool, [https://pacev2.apexcovantage.com](https://pacev2.apexcovantage.com/). PACE helps ensure that figures meet PLOS requirements. To use PACE, you must first register as a user. Then, login and navigate to the UPLOAD tab, where you will find detailed instructions on how to use the tool. If you encounter any issues or have any questions when using PACE, please email us at [figures@plos.org](mailto:figures@plos.org).
 
**Data Requirements:**
Please note that, as a condition of publication, PLOS' data policy requires that you make available all data used to draw the conclusions outlined in your manuscript. Data must be deposited in an appropriate repository, included within the body of the manuscript, or uploaded as supporting information. This includes all numerical values that were used to generate graphs, histograms etc. For an example see here: <http://www.plosbiology.org/article/info%3Adoi%2F10.1371%2Fjournal.pbio.1001908#s5>.
 
Reproducibility:
To enhance the reproducibility of your results, we recommend that you deposit your laboratory protocols in [protocols.io](http://protocols.io/), where a protocol can be assigned its own identifier (DOI) such that it can be cited independently in the future. Additionally, PLOS ONE offers an option to publish peer-reviewed clinical study protocols. Read more information on sharing protocols at <https://plos.org/protocols?utm_medium=editorial-email&utm_source=authorletters&utm_campaign=protocols>
